# Metabolic crosstalk between the heart and liver impacts familial hypertrophic cardiomyopathy

**DOI:** 10.1002/emmm.201302852

**Published:** 2014-02-24

**Authors:** Jason A Magida, Leslie A Leinwand

**Affiliations:** Department of Molecular, Cellular and Developmental Biology, BioFrontiers Institute, University of Colorado at BoulderBoulder, CO, USA

**Keywords:** cardiomyopathy, glucose, lipoproteins, liver

## Abstract

Familial hypertrophic cardiomyopathy (HCM) is largely caused by dominant mutations in genes encoding cardiac sarcomeric proteins, and it is etiologically distinct from secondary cardiomyopathies resulting from pressure/volume overload and neurohormonal or inflammatory stimuli. Here, we demonstrate that decreased left ventricular contractile function in male, but not female, HCM mice is associated with reduced fatty acid translocase (CD36) and AMP-activated protein kinase (AMPK) activity. As a result, the levels of myocardial ATP and triglyceride (TG) content are reduced, while the levels of oleic acid and TG in circulating very low density lipoproteins (VLDLs) and liver are increased. With time, these metabolic changes culminate in enhanced glucose production in male HCM mice. Remarkably, restoration of ventricular TG and ATP deficits via AMPK agonism as well as inhibition of gluconeogenesis improves ventricular architecture and function. These data underscore the importance of the systemic effects of a primary genetic heart disease to other organs and provide insight into potentially novel therapeutic interventions for HCM.

## Introduction

Familial hypertrophic cardiomyopathy (HCM) is a disorder affecting approximately 0.2% of the population and is the leading cause of sudden death in young people in the United States (Redwood *et al*, 1999). The R403Q mutation, located in the actin-binding domain of cardiac myosin heavy chain, is linked to a severe clinical phenotype (Seidman & Seidman, 2001), and transgenic mice with this mutation exhibit a number of pathological characteristics of the human disease, including cardiac hypertrophy followed by a sexually dimorphic end-stage phenotype characterized by progressive ventricular wall thinning, chamber dilation, contractile dysfunction, and heart failure (Hecht *et al*, 1993; Freeman *et al*, 2001; Harris *et al*, 2006).

Cardiomyopathies generally present an energy-starved state due to impaired cardiac fatty acid utilization and ATP synthesis, resulting in reduced ATP content (Beer *et al*, 2002; Neubauer, 2007; Lopaschuk *et al*, 2010). This energy-deprived condition leads to elevations in the activity of AMPK, a cellular sensor of the energetic state and a regulator of metabolic processes (Dolinsky & Dyck, 2006). AMPK activity, which is regulated by estrogen, phosphocreatine, adenine nucleotides, and long-chain fatty acids, facilitates catabolic processes to rectify energy starvation (Dolinsky & Dyck, 2006; D'Eon *et al*, 2008; Rogers *et al*, 2009). This process primarily occurs via (i) stimulation of fatty acid influx through the trafficking of fatty acid translocase (CD36) to the plasma membrane and (ii) de-repression of fatty acid oxidation (Dolinsky & Dyck, 2006). Cardiac-specific CD36-null mice have elevated very low density lipoprotein (VLDL), as clearance of these triglyceride (TG)-rich lipoproteins by the heart is substantially reduced (Bharadwaj *et al*, 2010). While HCM due to sarcomeric protein mutations is also known to be associated with an energy-starved state (Crilley *et al*, 2003), little is known about CD36 and AMPK activities in mouse models of sarcomeric mutations. However, depressed cardiac CD36 activity, reduced lipid clearance, and reduced cardiac TG content have been described in idiopathic HCM patients (Tanaka *et al*, 1997; Nakae *et al*, 2010).

Increased circulating lipid levels can result in TG accumulation in liver in addition to enhancing protein kinase C (PKC) and mitogen-activated protein kinase (MAPK) activities (Shinomura *et al*, 1991; Augustus *et al*, 2003; Collins *et al*, 2006). Such pathogenic signaling events activate the transcription of gluconeogenic genes, including phosphoenolpyruvate carboxykinase (PEPCK), leading to increased glucose production and hyperglycemia (Collins *et al*, 2006; Liu *et al*, 2007).

In this study, we show that HCM mice expressing a mutant α-myosin heavy chain (Vikstrom *et al*, 1996; Freeman *et al*, 2001) display impaired AMPK and CD36 activities, as well as a depletion of high-energy adenine nucleotide and TG. This cardiopathology, which is distinct from, for example, pressure overload, leads to elevated VLDL TG, as well as excessive hepatic lipid accumulation and PEPCK activity, culminating in increased glucose production. We demonstrate that ventricular contractile function can be restored either by activating AMPK or by inhibiting gluconeogenesis. These results provide insight into metabolic crosstalk between a primary genetic heart disease and the liver as well as novel mediators of HCM pathogenesis, offering potential therapeutic targets for improving cardiac function.

## Results

### Cardiac lipid uptake and content are reduced in male HCM mice

**Figure 1:**
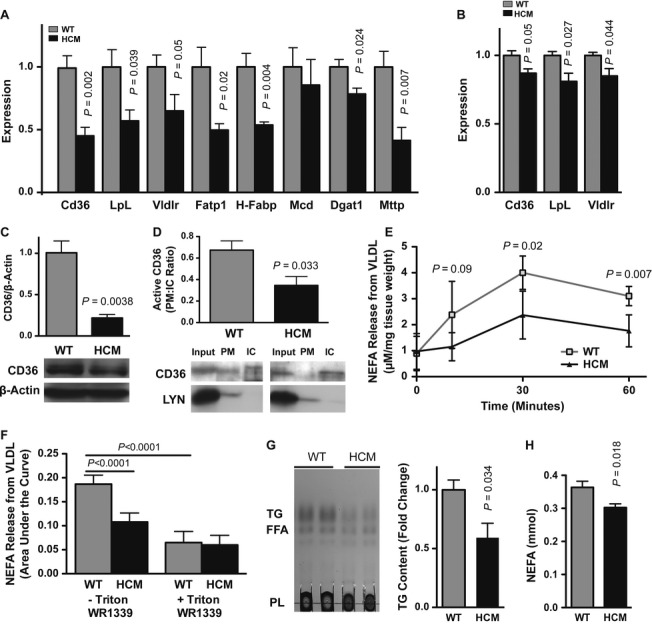
Reduced capacity for lipid clearance in the hypertrophic cardiomyopathy (HCM) heart at 12 months. A Fed mRNA levels of genes (determined by qRT-PCR) responsible for lipid clearance and handling; Cd36, lipoprotein lipase (LpL), very low density lipoprotein receptor (Vldlr), fatty acid transport protein (Fatp1), heart-type fatty acid-binding protein (H-Fabp), malonyl CoA decarboxylase (Mcd), diacylglycerol acyltransferase-1 (Dgat-1), microsomal triglyceride transfer protein (Mttp). Mean ± s.e.m.; *t*-test; *n *=* *5–7. B Fasted mRNA levels of Cd36, LpL, and Vldlr. Mean ± s.e.m.; *t*-test; *n *=* *3. C Cardiac CD36 protein immunoblot (normalized to β-actin). Mean ± s.e.m.; *t*-test; *n *=* *5–7. D Western blot analysis of plasma membrane (PM/LYN+) to intracellular vesicle membrane (IC/LYN-) ratio of CD36. Ratio of fractions normalized to input. Mean ± s.e.m.; *t*-test; *n *=* *4. E, F Non-esterified fatty acid (NEFA) release from VLDL by ventricular tissue. Expressed as tissue/VLDL incubation time (E), or area under the curve of a 60-min incubation in the presence or absence of the lipolytic inhibitor Triton WR1339 (F). Mean ± s.d.; *t*-test (E) or ANOVA (F); *n *=* *6. G Thin-layer chromatography of cardiac lipid extracts; TG, free fatty acids (FFA), phospholipids (PL). Mean ± s.e.m.; *t*-test; *n *=* *5. H Enzymatic determination of left ventricular NEFA content (normalized to protein). Mean ± s.e.m.; *t*-test; *n *=* *6.

To determine whether an inherited form of HCM results in impaired cardiac lipid utilization, we began by measuring the mRNA levels of key regulators of fatty acid clearance and transport in wild-type (WT) and HCM hearts. mRNAs encoding CD36, lipoprotein lipase, and VLDL receptor were significantly reduced in 12-month-old HCM hearts, an end-stage time-point at which HCM ventricular contractile dysfunction, mass, and chamber dilation are maximal (Fig 1A,B, Fig S1).

CD36 and lipoprotein lipase activities are integral to cellular lipoprotein recognition and fatty acid influx (Febbraio *et al*, 1999; Goudriaan *et al*, 2005). Both CD36 protein content and activity were decreased in the HCM heart (Fig 1C,D). This was accompanied by reduced VLDL TG hydrolysis by the HCM heart (Fig 1E,F, Fig S2). We next asked whether this reduced capacity for fatty acid release and uptake resulted in diminished myocardial lipid levels. We found decreases in cardiac TG and fatty acid content at 12 months (Fig 1G,H).

### Plasma lipid content reflects cardiac pathology in HCM mice

**Figure 2:**
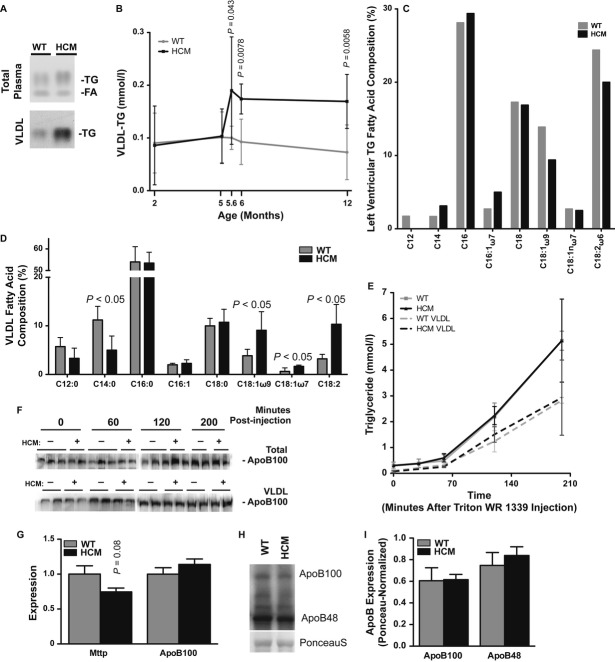
Hypertrophic cardiomyopathy (HCM) results in an accumulation of plasma triglycerides (TGs). A Thin-layer chromatography of whole plasma and very low density lipoprotein (VLDL) lipid extracts from fasted 12-month-old males. *n *=* *3–5. B Enzymatic determination of VLDL TG levels in 2-to 12-month-old males. Mean ± s.d.; *t*-test; *n *=* *5–8. C, D Gas chromatographic analysis of pooled left ventricular TG (C) and circulating VLDL fatty acid (D) composition. Mean (C) or Mean ± s.d. (D); *t*-test; *n *=* *5. E Measurement of TG secretion in plasma and VLDL following inhibition of peripheral lipolysis by Triton WR1339 administration to 12-to 15-month-old males. Mean ± s.d.; *t*-test; *n *=* *5–6. F Western blot of ApoB100 secretion in plasma and VLDL following Triton WR1339 administration. *n *=* *2–3. G Hepatic microsomal TG transfer protein (Mttp) and ApoB100 transcript levels. Mean ± s.e.m.; *t*-test; *n = *6. H, I Western blot analysis (H) and graphical representation (I) of hepatic ApoB protein levels (normalized to PonceauS) in 12-month-old males. Mean ± s.e.m.; *t*-test; *n = *5.

We hypothesized that reduced lipid clearance by the HCM heart, which occurs at least in part via a reduction in CD36 activity and VLDL TG hydrolysis, would result in unused TG accumulation in plasma (Febbraio *et al*, 1999; Goudriaan *et al*, 2005). There was, in fact, increased circulating VLDL TG in male HCM mice (Fig 2A,B). Furthermore, oleic acid that was reduced in the TG stores of the diseased heart accumulated in unutilized VLDL (Fig 2C,D). The loss of cardiac TG and increase in VLDL TG were not accompanied by changes in circulating LDL TG, catecholamines, and non-esterified fatty acids or body weight (Fig S3).

An alternative source of plasma lipid accumulation would be increased TG-rich particle secretion by the liver (Zhang *et al*, 2004). However, VLDL TG secretion rates and markers of hepatic VLDL production (e.g., apolipoprotein B) were unaltered in HCM males (Fig 2E–I, Fig S4).

### Unique metabolic defects of end-stage HCM

To gauge the importance of the loss of CD36 activity to disease progression, we examined the hearts of both younger HCM males and age-matched (12-month-old) HCM females that remain in a compensated hypertrophic state (Stauffer *et al*, 2006). In the absence of ventricular dilation and dysfunction, 12-month-old HCM females did not exhibit reduced CD36 expression or increased circulating TG (Fig S5A–D). HCM males first developed left ventricular dysfunction and dilation at 6 months of age, corresponding to the onset of CD36 downregulation and increased circulating TG, but preceding a loss of cardiac lipid content (Fig S5E–I). As an activator of CD36 expression and fatty acid uptake, we hypothesized that altered forkhead transcription factor FoxO1 activity could play a role in the lipid clearance deficiency associated with genetic heart failure (Bastie *et al*, 2005). In fact, FoxO1 was downregulated in the male HCM heart (Fig S6A).

To form a basis for comparison of this HCM model with other induced forms of cardiac pathology, we also assessed a pressure-overload model of heart failure induced by transverse aortic constriction. In contrast to end-stage HCM, cardiac CD36 protein and TG levels increased during pressure-overload-induced heart failure (Fig S6B–G). Furthermore, FoxO1 was upregulated by pressure overload (Fig S6H). Taken together, these data suggest that diminished CD36 activity in end-stage genetic heart disease contributes to reduced TG clearance by the failing myocardium.

### AMPK agonism improves cardiac function in HCM mice

**Figure 3:**
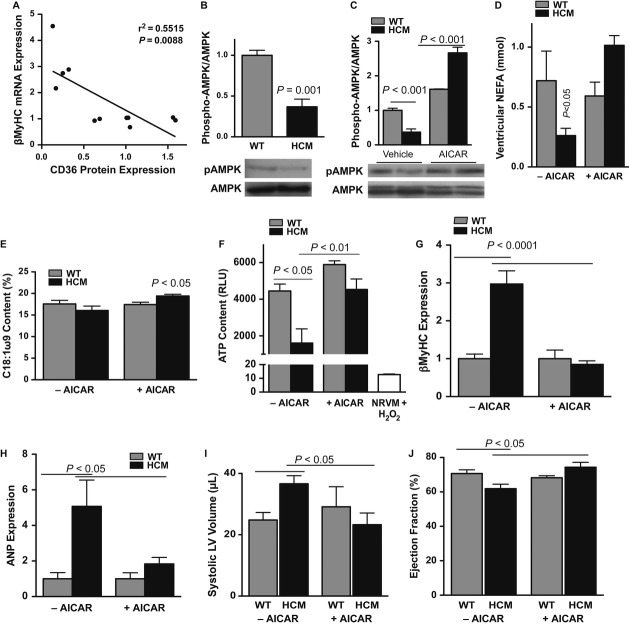
Ameliorating depressed AMPK activity in male hypertrophic cardiomyopathy (HCM) mice improves contractile function. A Regression analysis of ventricular CD36 protein and β-myosin mRNA at 12 months. B Western blot analysis of ventricular AMPK phosphorylation (normalized to total AMPK) in 12-month-old males. Mean ± s.e.m.; *t*-test; *n *=* *6–7. C Western blot analysis of total and phosphorylated AMPK in pooled ventricular lysates from mice treated with vehicle or 5-aminoimidazole-4-carboxamide 1-β-D-ribofuranoside (AICAR). Mean ± s.e.m.; ANOVA;*n *=* *2–8. D Enzymatic determination of ventricular non-esterified fatty acid (NEFA) content (normalized to protein). Mean ± s.e.m.; ANOVA; *n *=* *3. E Gas chromatographic determination of ventricular oleic acid (C18:1ω9) content. Mean ± s.e.m.; ANOVA; *n *=* *3. F Ventricular ATP content following vehicle or AICAR administration. Negative control; H_2_O_2_-treated NRVM (neonatal rat ventricular myocytes). Mean ± s.e.m.; ANOVA; *n *=* *4–8. G, H qPCR of ventricular β-Myhc (G) and ANP (H) expression in vehicle or AICAR-treated mice. Mean ± s.e.m.; ANOVA; *n *=* *3–5. I, J Echocardiographic determination of left ventricular volume in systole (I) and ejection fraction (J) before and after AICAR administration. Mean ± s.e.m.; ANOVA; *n *=* *4–14.

Ventricular CD36 downregulation correlates with increased pathological gene expression (as exemplified by β-myosin) at 12 months of age (Fig 3A). Because of an established role for AMPK in regulating CD36 expression and activity (Chabowski *et al*, 2006), we hypothesized that the loss of CD36 activity in the end-stage HCM heart was due to insufficient AMPK activity. The HCM heart did, in fact, display a progressive reduction in AMPK activity, paralleling CD36 downregulation (Fig 3B, Fig S7A–D). To demonstrate the significance of AMPK inactivity specifically to HCM males with contractile dysfunction, we assessed ventricular AMPK phosphorylation in 12-month-old HCM females or WT males subjected to pressure overload and found no decrease in AMPK activation (Fig S7E,F). Because contractility and fatty acids enhance AMPK activity (Dolinsky & Dyck, 2006), the decrease in AMPK activity in HCM males with contractile dysfunction may be due to the observed decreases in ventricular lipid load and contractile rate associated with genetic heart failure, but not pressure overload (Fig S7G). We then examined whether the loss of CD36 and AMPK activities was accompanied by corresponding pathological insults, such as high-energy phosphate depletion. Like the dilated human heart (Beer *et al*, 2002), we observed progressive ATP exhaustion in the end-stage HCM male heart (Fig S7H).

Finally, we tested whether the AMPK agonist 5-aminoimidazole-4-carboxamide 1-β-D-ribofuranoside (AICAR) would neutralize the ventricular energy deficit and dysfunction in this normotensive model of heart failure (Fig S8A,B). AICAR administration restored AMPK activity, upregulated CD36, and increased TG and oleic acid content in the HCM heart (Fig 3C,E, Fig S8C–E). AICAR also increased cardiac ATP content, reduced pro-apoptotic cleaved caspases-3/9 levels, normalized the expression of fetal genes that are considered a hallmark of cardiac pathology (e.g., β-myosin, ANP), and reduced ventricular chamber dilation without impacting cardiac glycogen content or circulating insulin, glucose, non-esterified fatty acid, and TG levels (Fig 3F–J, Fig S8F–J). Most importantly, AICAR restored contractile function in HCM mice, as demonstrated by increased ejection fraction (Fig 3J).

### Hepatic lipid accumulation in HCM

**Figure 4:**
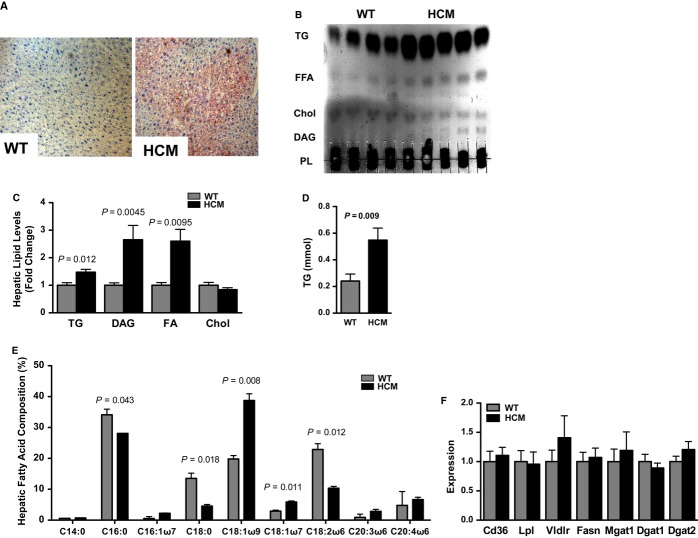
Hepatic lipid accumulation in 12-month-old male hypertrophic cardiomyopathy (HCM) mice. A Representative oil-red-O-stained WT and HCM liver sections. B, C Thin-layer chromatography (B) and graphical presentation of TG, diacylglycerol (DAG), free fatty acids (FA) and cholesterol (C) of hepatic lipid extracts. Mean ± s.e.m.; *t*-test; *n *=* *4–5. D Enzymatic measurement of hepatic triglyceride (TG) content (normalized to protein). Mean ± s.e.m.; *t*-test; *n *=* *5. E Gas chromatographic assessment of hepatic fatty acid composition. Mean ± s.d.; *t*-test; *n *=* *4. F qPCR analysis of hepatic lipid clearance and lipogenic gene expression; Cd36, lipoprotein lipase (Lpl), very low density lipoprotein receptor (Vldlr), fatty acid synthase (Fasn), monoacylglycerol acyltransferase-1 (Mgat-1), diacylglycerol acyltransferases-1/2 (Dgat-1/2). Mean ± s.e.m.; *t*-test; *n *=* *6–8.

Because the liver sequesters excess circulating lipoproteins (Augustus *et al*, 2003), we analyzed hepatic lipid load. We found that the loss of ventricular CD36 and lipoprotein lipase expression paralleled hepatic lipid accumulation as early as 6 months of age (Fig S9A–F). In contrast to the HCM heart, the HCM liver exhibited increased neutral lipid levels and oleic acid content at 12 months of age, independent of gene expression promoting lipid clearance or biosynthesis (Fig 4).

**Figure 5:**
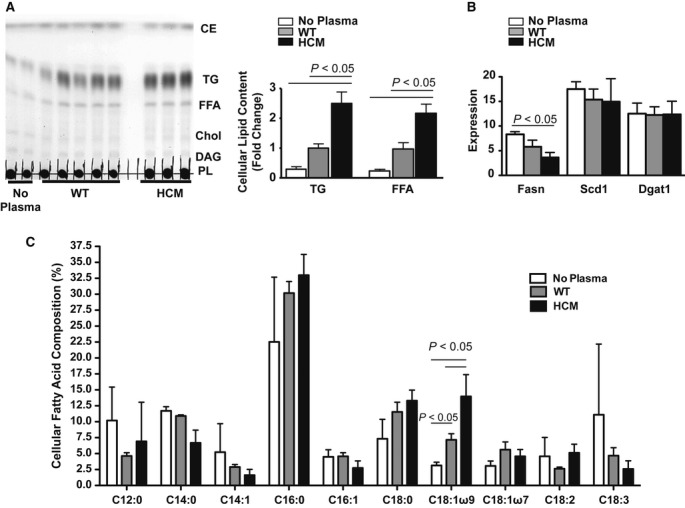
Lipid deposition in FaO hepatocytes cultured with hypertrophic cardiomyopathy (HCM) plasma.
Left panel: TLC of intracellular lipids in FaO cells following incubation in media alone or fasted plasma from 12-month-old male WT or HCM littermates. Right panel: graphical presentation of data. Mean ± s.e.m.; ANOVA; *n *=* *3–5.qPCR analysis of lipogenic gene transcript levels in FaO cells; fatty acid synthase (Fasn), stearoyl CoA desaturase-1 (Scd-1), diacylglycerol acyltransferase-1 (Dgat-1). Mean ± s.e.m.; ANOVA; *n *=* *3.Gas chromatographic analysis of fatty acid composition in triglyceride (TG) and free fatty acids extracted from plasma-cultured FaO cells. Mean ± s.d.; ANOVA; *n *=* *3.

To pursue the cellular mechanisms underlying these observations, we asked whether FaO hepatoma cells cultured with plasma from HCM mice would recapitulate the lipid profile observed in the HCM liver. Consistent with our observations *in vivo,* hepatocytes cultured with HCM plasma accumulated TG and fatty acids, in the absence of lipogenic gene activation and cholesterol buildup (Fig 5A,B, Fig S9G,H). In addition to TG accumulation, both the VLDL fraction and hepatocytes cultured with plasma from 12-month-old HCM mice displayed increased levels of oleic acid, representing an overlapping lipid signature between circulating lipoproteins and hepatocytes cultured with HCM plasma (Fig 5C, Fig S9I).

### Primary heart disease affects hepatic function

**Figure 6:**
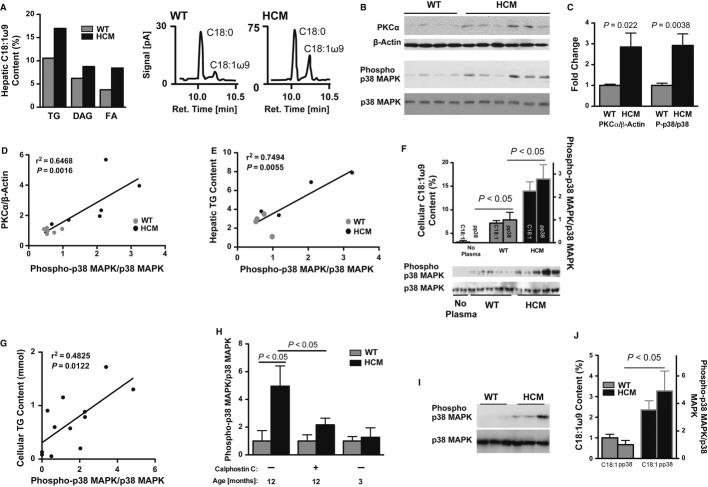
Altered hepatic signaling in 12-month-old hypertrophic cardiomyopathy (HCM) males. A Oleic acid (C18:1ω9) content in hepatic triglyceride (TG), diacylglycerol (DAG), and free fatty acid (FA) pools, as well as chromatographs of stearic acid (C18:0) and oleic acid in the hepatic fatty acid pool. *n *=* *5. B, C Western blot analysis (B) and corresponding quantified data (C) of PKCα (normalized to β-actin) and phosphorylated/total p38 mitogen-activated protein kinase (MAPK). Mean ± s.e.m.; *t*-test; *n *=* *6–7. D, E Regression analyses of hepatic phosphorylated p38 MAPK versus PKCα (D) or TG (E) content. F Western blots of phosphorylated and total p38 MAPK in hepatocytes cultured with media containing no, WT or HCM plasma. Mean ± s.e.m.; ANOVA; *n *=* *5. G Regression analysis of phosphorylated p38 MAPK and TG content in FaO cells. H ELISA of p38 MAPK phosphorylation when FaO cells were incubated with fasted plasma from 3-or 12-month-old male mice ± calphostin C, an inhibitor of PKC. The ratio of phosphorylated to total p38 MAPK was normalized to cell number (determined by crystal violet staining). Mean ± s.e.m.; ANOVA; *n *=* *4. I, J Western blot analysis (I) and corresponding quantified data (J) of phosphorylated and total p38 MAPK levels in FaO hepatocytes cultured with media containing very low density lipoprotein (VLDL) lipid extracts from fasted WT or HCM plasma. Graph depicts cellular p38 MAPK phosphorylation and oleic acid content of VLDL extracts supplemented to the culture media. Mean ± s.e.m.; *t*-test; *n *=* *3.

Comparable to the circulating VLDL fatty acid profile, oleic acid was enriched in the TG, diacylglycerol, and free fatty acid fractions of the end-stage HCM liver (Fig 6A, Fig S10A). Diacylglycerol and oleic acid are potent agonists of pathogenic intracellular signaling mediators, such as PKC and MAPK (Shinomura *et al*, 1991; Lo *et al*, 1994; Collins *et al*, 2006). Western blot analyses revealed increased PKCα protein levels and p38 MAPK phosphorylation, which were dependent upon the hepatic TG accumulation found associated with end-stage HCM at 12 months of age (Fig 6B–E, Fig S10B–G). Supporting a role for PKC in mediating p38 MAPK activity, was increased PKCα phosphorylation and co-immunoprecipitation with p38 MAPK in the end-stage HCM liver (Fig S10H,I).

FaO hepatocytes were cultured with plasma from healthy and diseased mice to assess the MAPK-activating capacity of circulating lipids. Incubating hepatocytes with the lipid-enriched end-stage HCM plasma, from male mice at 12 or 3 months of age, resulted in oleic acid and TG-dependent increases in p38 MAPK phosphorylation only in response to plasma from 12-month-old mice (Fig 6F–H, Fig S10J). This effect was largely abolished by the PKC inhibitor calphostin C (Fig 6H; Gopalakrishna *et al*, 1992). To further test the lipid dependence of PKC-mediated p38 MAPK activation, we cultured FaO hepatocytes with different lipoprotein fractions and the lipid extracts of those fractions. p38 MAPK was robustly activated by lipid extracts of VLDL from end-stage HCM plasma (Fig 6I–J).

**Figure 7:**
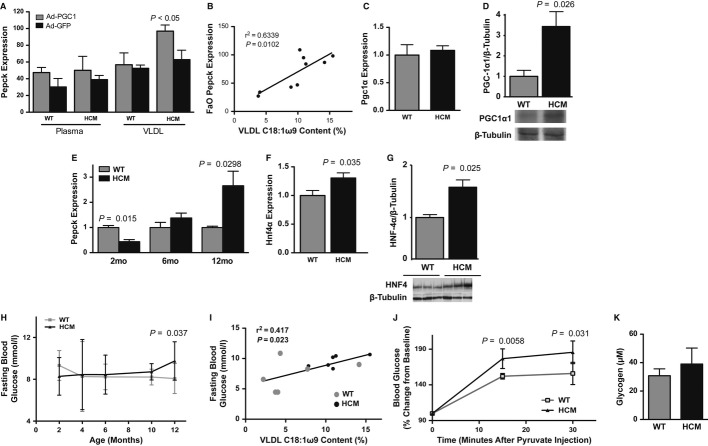
Hepatic gluconeogenic response to end-stage hypertrophic cardiomyopathy (HCM).
Pepck expression in FaO cells cultured with whole plasma or very low density lipoprotein (VLDL) and infected with adenovirus expressing PGC-1α or GFP. Mean ± s.e.m.; ANOVA; *n *=* *3–5.Regression analysis of VLDL oleic acid (C18:1ω9) content supplemented to culture medium and Pepck expression of FaO hepatocytes (infected with PGC1-expressing adenovirus).qPCR of hepatic Pgc1α. Mean ± s.e.m.; *t*-test; *n *=* *6–8.Western blot of hepatic PGC-1α (normalized to β-tubulin). Mean ± s.e.m.; *t*-test; *n *=* *7.qPCR of hepatic Pepck transcript levels in males. Mean ± s.e.m.; *t*-test; *n *=* *2–6 (2 m), *n *=* *6 (6 m), *n *=* *6 (12 m).qPCR of Hnf4α. Mean ± s.e.m.; *t*-test; *n *=* *6–8.Western blot of HNF-4α (normalized to β-tubulin). Mean ± s.e.m.; *t*-test; *n *=* *4.Timeline of fasting blood glucose levels in HCM males. Mean ± s.d.; *t*-test; *n *=* *7–9 (2 m), *n *=* *8–9 (4 m), *n *=* *6 (6 m), *n *=* *3–5 (10 m), *n *=* *10–11 (12 m).Regression analysis of circulating VLDL oleic acid (C18:1ω9) content and blood glucose. *n *=* *5–6.Blood glucose following pyruvate injection. Mean ± s.d.; *t*-test; *n *=* *4–6.Hepatic glycogen-derived glucose levels in 12-month-old males. Normalized to wet tissue weight. Mean ± s.e.m.; *t*-test; *n *=* *5.

HCM-induced oleic acid accumulation and MAPK activation in the liver may facilitate the phosphorylation and stabilization of peroxisome proliferator-activated receptor-γ coactivator-1α (PGC-1α) protein, and stimulation of a PGC1α-driven transcriptional program (Puigserver & Spiegelman, 2003; Collins *et al*, 2006). Therefore, we examined PGC1α-dependent activities for evidence of a myocardium-derived or excluded effector that would alter liver function. In support of this, the PGC-1α target phosphoenolpyruvate carboxykinase (PEPCK) was upregulated in FaO hepatocytes infected with PGC1α-expressing adenovirus and cultured with HCM VLDL isolates, in an oleic acid-dependent manner (Fig 7A,B, Fig S11A,B). Notably, increased p38 MAPK phosphorylation and PEPCK expression occurred independently of adrenergic, oxidative, hypoxic, or inflammatory stress (Fig S11C–I).

Corroborating the *in vitro* consequences of oleic acid accumulation (Puigserver & Spiegelman, 2003; Collins *et al*, 2006) was evidence suggestive of increased PGC-1α phosphorylation by phospho-p38 MAPK and increased levels of PGC-1α protein, but not transcripts, in the HCM liver (Fig 7C,D, Fig S12A–E). Consistent with PGC-1α phosphorylation was increased PGC-1 bound to the *Pepck* promoter, and the mRNA levels of the PGC-1α target, PEPCK, were elevated in the end-stage HCM male liver (Fig 7E, Fig S12F,G). The activation of a PGC-directed transcriptional program included hepatocyte nuclear factor-4 upregulation in the HCM liver (Fig 7F,G, Fig S12H,I). Notably, lipid accumulation and PEPCK upregulation appeared to be independent of hepatic adrenergic receptor, glucocorticoid receptor, or peroxisome proliferator-activated receptor-α activities and were not detected in the kidneys of HCM males (Figs S13, 14).

We hypothesized that the induction of hepatic PGC-1α activity and PEPCK-mediated gluconeogenesis would result in elevated levels of circulating glucose in HCM mice. We observed elevated fasting blood glucose levels and gluconeogenic activity in end-stage HCM males, independently of hepatic glycogen depletion (Fig 7H–K).

### Altered hepatic function is specific to end-stage HCM in males

Age-matched HCM females, which retain ventricular function and are devoid of gluconeogenic stimuli (e.g., increased plasma/hepatic lipids and p38 MAPK activity) or PGC-1α target activation, did not exhibit elevated glucose production or blood glucose levels (Fig S15). Although pressure overload yielded ventricular dysfunction, it did not result in increased plasma and VLDL TG, hepatic TG accumulation and p38 MAPK activation, PGC-1α target upregulation, or elevated glucose levels (Fig S16). Therefore, excessive glucose production appears to be specific to the systemic metabolic consequences of heart failure due to HCM.

### Inhibition of gluconeogenesis restores cardiac function in HCM mice

**Figure 8:**
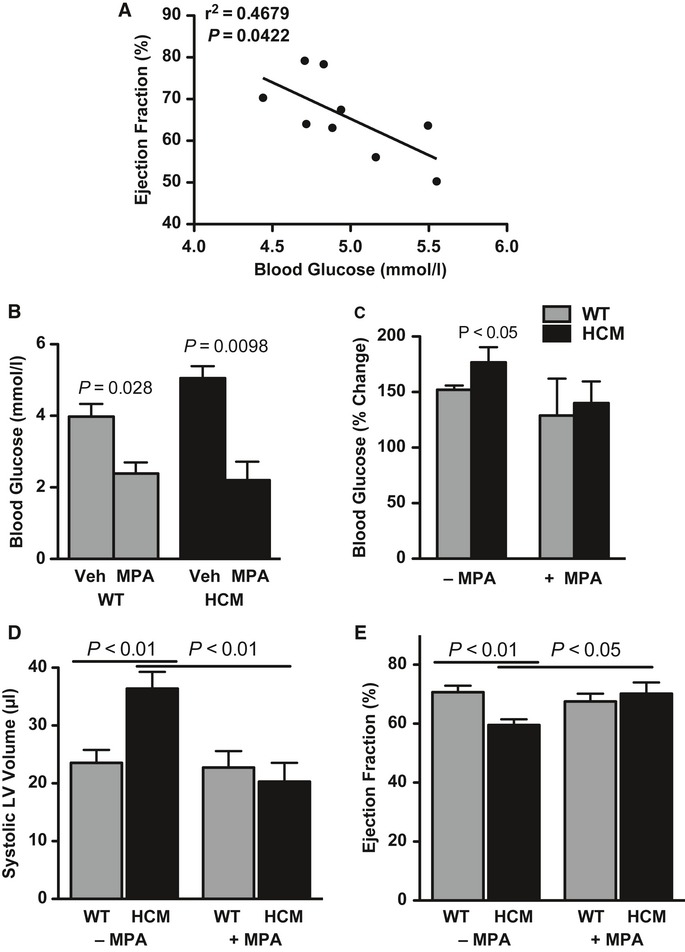
Inhibition of gluconeogenesis rescues cardiac function. A Regression analysis of left ventricular function (ejection fraction) and circulating glucose in 12-to 15-month-old male mice. B Potent inhibition of phosphoenolpyruvate carboxykinase (PEPCK) activity by 3-mercaptopicolinic acid (3-MPA) administration is reflected by decreased circulating glucose. Mean ± s.e.m.; *t-test*; *n *=* *3. C Pyruvate-induced glucose production following 3-MPA administration (normalized to pre-injection values). Mean ± s.e.m.; ANOVA; *n* = 5–7. D, E Echocardiographic determination of systolic left ventricular volume (D) and ejection fraction (E) in mice treated with vehicle or 3-MPA. Mean ± s.e.m.; ANOVA; *n *=* *8–12.

Increased fasting blood glucose levels correlated with reduced ventricular function in male mice (Fig 8A). Although end-stage HCM resulted in elevated monoacylglycerol acyltransferase-2 expression and diacylglycerol levels in the heart, all other indicators of damage induced by excessive glucose exposure were absent in the HCM heart (Fig S17; Mostafa *et al*, 1993; Depre *et al*, 2000; Chen *et al*, 2010; Jeong *et al*, 2011).

To test whether the observed activation of hepatic gluconeogenesis and increased blood glucose levels contribute to cardiac dysfunction in HCM males, and to avoid the systemic compensatory mechanisms of a liver-specific PEPCK null mouse, we administered 3-mercaptopicolinic acid (3-MPA), a specific inhibitor of PEPCK activity (DiTullio *et al*, 1974; Jomain-Baum *et al*, 1976; She *et al*, 2000; Yang *et al*, 2008). 3-MPA effectively reduced elevated glucose production and blood glucose without influencing heart rate or plasma TG, non-esterified fatty acid, or catecholamine levels (Fig 8B,C, Fig S18). Although 3-MPA normalized pro-apoptotic cleaved caspase-9, monoacylglycerol acyltransferase-2, and sarcoplasmic reticulum calcium ATPase levels, it failed to reduce the expression of fetal genes, including β-myosin and ANP (Fig S19A–E). Strikingly, the suppression of excessive PEPCK-mediated gluconeogenesis reversed ventricular chamber dilation and cardiac dysfunction in HCM mice (Fig 8D,E, Fig S19F).

## Discussion

Cardiac myosin mutations are a primary or cardiac-specific insult, distinguishing end-stage familial HCM from cardiomyopathies secondary to pressure or volume overload, coronary disease, infarction, ischemia, pacing, diabetes, or obesity. Unlike genetic HCM, these secondary conditions, as well as idiopathic cardiomyopathies, are often associated with insulin resistance, elevated catecholamines, cachexia, or inflammatory cytokines and may result in elevated plasma non-esterified fatty acids (Redwood *et al*, 1999; Seidman & Seidman, 2001; Cambronero *et al*, 2009; Lopaschuk *et al*, 2010). In this study, we demonstrate that genetic HCM results in impaired cardiac lipid clearance and storage, as well as an energy-deficient state. In contrast to the reductions in cardiac TG content observed in hypertensive or volume-overload models of heart failure, the lipid loss observed in the HCM heart is due to intrinsic alterations in cardiac metabolism, rather than a secondary response to elevated catecholamines or inflammatory cytokines and systemic cachexia (O'Donnell *et al*, 2008; Kato *et al*, 2011; Melenovsky *et al*, 2011a; Table S1A). Energy starvation and reduced fatty acid extraction from VLDL TG by the murine HCM heart (rather than increased VLDL particle number) are consistent with descriptions of diminished lipid clearance and TG storage in the hearts of idiopathic HCM patients (Tanaka *et al*, 1997; Tadamura *et al*, 1998; Beer *et al*, 2002; Nakae *et al*, 2010).

Since the heart is a principal lipolytic organ, the proposed defects in lipoprotein clearance caused by mutant myosin expression result in elevated circulating VLDL TG and oleic acid in HCM males. However, normal hepatic lipoprotein secretion rates indicate that a rise in plasma TG is not an artifact of adipose-derived non-esterified fatty acid release and subsequent re-esterification by the liver in HCM males. In contrast to the normotensive HCM mouse, many heart failure models are generated by inducing or mimicking a hypertensive or pressure-overloaded state. However, these surgical and dietary manipulations may result in inflammatory cytokine, catecholamine, and free fatty acid surges, as well as substantial diabetic complications, increased hepatic fatty acid synthase activity, and elevated plasma cholesterol levels (Shimizu *et al*, 2010; Kato *et al*, 2011; Melenovsky *et al*, 2011b). Similarly, idiopathic cardiomyopathies in human subjects are associated with corresponding complications, such as inflammatory or hyperadrenergic states, obscuring the primary etiology and systemic consequences (Omodani *et al*, 1998; Cambronero *et al*, 2009; Lopaschuk *et al*, 2010). These confounding elements are absent in our hereditary HCM model of cardiac dysfunction, allowing familial HCM to be distinguished from secondary cardiomyopathies and any extra-cardiac phenotypes to be traced directly to the failing heart (Table S1A).

The relationship between disrupted lipid use by the heart and the pathophysiology of HCM in males is underscored by three observations. First, the cardiac exclusion and buildup of plasma TG occurs concomitantly with the deterioration of cardiac structure and function in HCM mice. Second, HCM females and pressure-overloaded mice do not exhibit reductions in ventricular CD36 expression or AMPK activity, increased plasma TG, or the downstream hepatic repercussions observed in male HCM mice (Table S1A–C). Third, normalizing lipid uptake by the pharmacological re-activation of AMPK ameliorates cardiac CD36 and lipid depletion, energy starvation, dysfunction, dilation, and pathological gene expression in male HCM mice. While numerous studies of the infarcted, paced, or pressure/volume-overloaded heart have described the enhancement of cardiac function with the AMPK agonist metformin, these cardiac disease models display features of neurohormonal, diabetic, or inflammatory stress and maintained or increased AMPK activity, indicating an alternative mode of action for metformin and underscoring the unique features of HCM (Tian *et al*, 2001; Dolinsky & Dyck, 2006; Saeedi *et al*, 2008; Sasaki *et al*, 2009; Benes *et al*, 2011; Wang *et al*, 2011; Table S2).

An important aspect of our study is the demonstration of a pathological link between the metabolic functions of the heart and liver, both organs serving as essential VLDL TG clearance sites (Augustus *et al*, 2003). Increased TG and oleic acid in the VLDL fraction, which we posit is the result of a decreased capacity for cardiac lipid disposal, accumulates in the HCM liver. Accordingly, p38 MAPK interacts with a lipid-sensitive PKC isozyme in the HCM liver and is efficiently activated *in vitro* by HCM VLDL lipid extracts, and its activity is abrogated by PKC inhibition. Consistent with the accumulation of TG derivatives, particularly diacylglycerol and oleic acid, genetic heart disease results in a plasma lipid-initiated and PKC-dependent activation of hepatic MAPK.

We show that the VLDL-initiated and MAPK-mediated activation of PGC-1α results in excessive gluconeogenesis. The finding that HCM females and young HCM males, devoid of abnormal cardiac function and architecture, do not display elevated hepatic PEPCK expression or circulating glucose levels emphasizes the pathological implications of hepatic feedback.

In summary, we propose a model whereby pathological alterations in cardiac lipid utilization activate a distal PKC-dependent MAPK signaling cascade that promotes excessive hepatic PEPCK expression and gluconeogenesis in HCM mice, in a sexually dimorphic manner (Fig S20). We demonstrate that ventricular function can be restored by the pharmacological rectification of cardiac substrate metabolism or by inhibition of excessive hepatic gluconeogenesis (Tables S2, S3). These results identify (i) novel mechanisms for ameliorating genetic heart disease, (ii) metabolic and inflammatory discrepancies between a genetic cardiomyopathy and other drivers of heart failure, (iii) putative sources of the clinical heterogeneity associated with HCM, and (iv) the importance of crosstalk between the heart and liver during the progression of primary genetic heart disease.

## Materials and Methods

### Animal use and care

The HCM mouse model used in this study expresses a mutant rat α-MyHC with expression driven by an α-MyHC promoter on a C57Bl/6 background (Vikstrom *et al*, 1996). The transgene coding region contained two mutations, a point mutation, R403Q, and a deletion of 59 amino acids in the actin-binding site bridged by the addition of nine non-myosin amino acids. All mice were fed a standard rodent chow diet (Teklad 8640) *ad libitum*. Mice were anaesthetized with isoflurane and sacrificed by cervical dislocation between 13:00 and 17:00 h. Exsanguination by cold PBS perfusion was followed by removal of tissues and immediate freezing in liquid nitrogen.

### Quantitative PCR

Total RNA was extracted from ventricles and livers using TRI Reagent (Life Technologies, Grand Island, NY, USA). Two micrograms of RNA was reverse-transcribed into cDNA using the SuperScript III first-strand cDNA synthesis kit (Life Technologies). Real-time PCR was performed using the ABI7500 system. Gene expression was normalized to 18S ribosomal RNA and calculated as relative change. Typically, 20 ng (gene of interest) or 0.4 ng (18S) of cDNA, 0.25 μM of each primer, and SYBR Green Master Mix (Life Technologies) were used for qPCR. (See Supplementary Table S4 for primer sequences and Supplementary Materials and Methods description of chromatin immunoprecipitation and PCR.)

### ATP Content

Left ventricles were homogenized in cold PBS, sonicated, and centrifuged at 4°C. Supernatant ATP and ADP contents were measured by the EnzyLight™ ADP/ATP Ratio assay kit (BioAssay Systems, Hayward, CA, USA, ELDT-100) according to the manufacturer's instructions.

### SDS–PAGE and Western blotting

Thirty milligrams of left ventricles and livers was homogenized and sonicated in RIPA (50 mM Tris–HCl pH 8, 150 mM NaCl, 0.5% sodium deoxycholate, 1% NP-40, 0.1% SDS) supplemented with complete EDTA-free protease inhibitor (Roche, Indianapolis, IN, USA), 1 mM phenylmethylsulfonyl fluoride, 1 mM sodium pyrophosphate, 1 mM sodium molybdate, 1 mM sodium orthovanadate, 2 mM sodium fluoride, and centrifuged at 14,000 *g* for 20 min. Twenty-five micrograms of lysate (in β-mercaptoethanol-containing buffer) was resolved in a 9% (or 4–20% gradient for APOB) polyacrylamide gel and analyzed by Western blot. For APOB SDS–PAGE, 5 μl plasma or lipoprotein fractions were combined 1:2 with RIPA and loading buffer, boiled, and run 24 h. (See Supplementary Materials and Methods for immunoprecipitation protocol and antibodies.)

### Plasma membrane fraction

Plasma membrane and intracellular giant sarcolemmal vesicle fractions were isolated as previously described (Han *et al*, 2007). (See Supplementary Materials and Methods.)

### Enzyme-linked immunosorbent assays

Cytokines were determined by the mouse cytokine Milliplex 13, performed by Biomarker Services at Millipore Bioscience (St. Charles, MO, USA) on the Luminex xMAP platform. Catecholamine concentrations were determined by the 3-CAT or norepinephrine ELISA (Labor Diagnostika Nord #BAE-5600 or 5200; Nordhorm, Germany), glucocorticoids measured using the cortisol or corticosterone ELISA (Arbor Assays #K003-H1 or K014-H1; Ann Arbor, MI, USA), and insulin measured using a rat/mouse insulin ELISA (Millipore #EZRMI-13K; Billerica, MA, USA), per manufacturer's instructions.

### Glucose measurements

For glucose and pyruvate tolerance tests, mice were fasted 6 or 18 h and given an IP injection of (2 g/kg body weight) glucose or pyruvate, respectively, followed by glucose determinations at 0-to 120-min time-points. Blood glucose was determined using a handheld glucometer (BD Bioscience, Franklin Lakes, NJ, USA) at 16:00 h in the absence of anesthesia. Glycogen was measured as glucose released by amyloglucosidase as previously described (Passonneau & Lauderdale, 1974).

### Lipid analyses

Blood was removed after a 6-h fast by retro-orbital eye bleeding at 16:00 h with the use of isoflurane. Tissue, plasma, and lipoprotein fraction TG and non-esterified free fatty acid content and cholesterol were measured using thin-layer chromatography and commercial kits from Wako Diagnostics (Richmond, VA, USA, L-type TG H, NEFA-HR 2, and Chol-E) per manufacturer's instructions and normalized to protein content. Lipoprotein fractions were isolated as previously described (Teupser *et al*, 2004). VLDL TG hydrolysis was measured by homogenization of ventricular tissue in buffer (150 mM NaCl, 10 mM Tris, 2 mM EDTA, pH 7.4), then incubating lysates with VLDL and buffer or Triton WR1339 for 0–60 min at 37°C. Following incubation period, samples were immediately frozen for storage, and NEFA was measured by the aforementioned enzymatic assays. Blood and tissue lipid peroxide levels were determined by assaying for TBARS (Cayman Chemical #100009055; Ann Arbor, MI, USA) according to the manufacturer's instructions and normalized to protein content. Frozen livers were sectioned and Oil Red O stained by Premier Histology. (See Supplementary Materials and Methods for chromatography methods.)

### Hepatic triglyceride secretion

Overnight-fasted (12-to 15-month-old) mice were injected with 900 mg/kg body weight Triton WR1339 (Sigma #T0307; St. Louis, MO, USA ). Blood was drawn at 0, 30, 60, 120 and 200 min post-injection for enzymatic measurements of total plasma and VLDL TG, as well as Western blotting for APOB levels in circulation.

### Cell culture

FaO hepatoma cells were maintained as specified by ATCC. Cells were grown to 80% confluence in DMEM containing 20% horse and 5% FBS. Cultures were serum-starved before each experiment, whereby DMEM contained 10% fasting plasma, lipoprotein fraction, or reconstituted lipid extracts. qRT-PCR and immunoblot experiments were cultured in 12-well plates for 48 h before being washed with cold PBS and cells scraped into Trizol or RIPA, respectively. Cells were alternatively cultured in a 96-well plate when infected with adenovirus or p38 MAPK activity was measured by a cell-based enzyme-linked immunosorbent assay (Active Motif #48100; Carlsbad, CA, USA) according to the manufacturer's instructions. (See Supplementary Materials and Methods for cardiomyocyte culture protocol.)

### Pharmacological interventions

Baseline echocardiographic and glucose measurements of 12-to 15-month-old male mice were taken in the fasted state 2–5 days prior to 3-MPA or vehicle IP injections, followed by resumption of *ad libitum* feeding. Mice were then fasted 12 h before an IP injection of 3-MPA [100 mg/kg body weight] in 1% starch/saline (w/v) suspension or vehicle alone. Echocardiographic and glucose measurements were followed by a second 3-MPA injection [25 mg/kg] and sacrifice 2 h later. 12-to 15-month-old male baseline plasma lipid or blood glucose and echocardiographic measurements were performed 1 day before AICAR [500 mg/kg body weight] in saline was IP injected. Mice received 5 AICAR or saline injections over the course of a week, followed by echocardiography and sacrifice on the 8 days.

**The paper explained**

**Problem**

End-stage familial HCM is due to a cardiac-specific genetic defect. This pathology can be distinguished from other cardiomyopathies by the absence of pre-existing metabolic, neurohormonal, and inflammatory confounders. Therefore, the study of familial HCM may offer insight into metabolic and systemic adaptations stemming directly from the diseased cardiac myocyte.

**Results**

We demonstrate that a primary cardiac myocyte defect leads to aberrant lipid accumulation and signaling in the liver. These systemic effects depend upon sex and disease progression. The resulting hepatic phenotypes, particularly the increase in blood glucose levels, significantly impact cardiac function. Importantly, normalizing lipid delivery defects in the heart or inhibition of excessive glucose production in the liver improves ventricular contractile dysfunction in HCM mice.

**Impact**

Our study illustrates clear metabolic distinctions between heart failure resulting from the cardiac-specific expression of a sarcomeric mutant protein and pressure-overload models. Moreover, our findings uncover a novel crosstalk between a cardiac-specific defect and hepatic metabolism and provide a basis for a better understanding of the clinical heterogeneity of HCM. Finally, our data could be used in the development of better diagnostics and treatments for genetic heart disease progression.

### Echocardiography, banding, and blood pressure measurements

Measurements of cardiac function and dimensions were made blinded in M-mode, using a Philips Sonos 5500 with a 15–6 MHz linear array transducer, three times and then averaged. For blood pressure measurements, the right carotid artery was dissected and isolated. A PE50 fluid-filled catheter was placed in the carotid artery. Steady-state systemic hemodynamics were obtained using the I-Worx (model #BP-100) fluid-filled catheter system. (See Supplementary Materials and Methods for echocardiographic formulae and banding protocol.)

### Statistics

Error bars represent s.e.m. unless otherwise noted. Statistical analyses were performed using two-tailed Student's *t*-test, or one-way ANOVA (and Bonferroni correction) where appropriate, with a *P*-value of ≤ 0.05 considered to be statistically significant.

### Study approval

All animal experiments were approved by the Institutional Animal Care and Use Committee at the University of Colorado at Boulder.
