# Supplementary material for: Metabolic crosstalk between the heart and liver impacts familial hypertrophic cardiomyopathy
Source: EMBO Mol Med. 2014 Feb 24;6(4):482–95. doi: 10.1002/emmm.201302852 (PMC3992075; doi:10.1002/emmm.201302852)
Supplement: Supplementary file 1 [file emmm0006-0482-sd1.pdf]

Metabolic Crosstalk between the Heart and Liver Impacts Familial Hypertrophic Cardiomyopathy

Jason A. Magida and Leslie A. Leinwand

Corresponding author: Leslie A. Leinwand, University of Colorado at Boulder

Review timeline:

Submission date:	05 April 2013
Editorial Decision:	14 May 2013
Revision received:	03 September 2013
Editorial Decision:	19 September 2013
Revision received:	04 November 2013
Editorial Decision:	03 December 2013
Revision received:	20 December 2013
Accepted:	26 December 2013

Transaction Report:

(Note: With the exception of the correction of typographical or spelling errors that could be a source of ambiguity, letters and reports are not edited. The original formatting of letters and referee reports may not be reflected in this compilation.)

Editor: Roberto Buccione

1st Editorial Decision 14 May 2013

Thank you for the submission of your manuscript to EMBO Molecular Medicine. We have now heard back from the three Reviewers whom we asked to evaluate your manuscript.

You will see that all three Reviewers are generally supportive of you work although Reviewer 2, in particular, expresses significant concerns that prevent us from considering publication at this time.

Reviewer 1 lists a number of issues that require clarification on your part. In general, s/he feels, similarly to Reviewer 2, that the manuscript would benefit from an extensive overhaul to increase organisation, readability and impact.

Reviewer 2, while appreciating the potentially interesting contribution, is quite critical of the manuscript and feels that, as it stands, it appears over-reaching with respect to the provided experimental evidence and raises important caveats. I will not dwell into much detail, as the comments are self-explanatory. I would like, however, to highlight the main points. Firstly, Reviewer 2 is of the opinion that the main finding of the study is based on indirect and not univocal results. S/he suggests that proper metabolic tracing should be conducted to determine metabolic flux through the heart and phospho-NMR to investigate ATP concentrations. Another issue is that clear evidence for enhanced hepatic glucose output is lacking and would require hyperinsulinemic-euglycemic camp studies to reach a definite conclusion. Reviewer 2 also feels that tandem ChIP would be required to actually demonstrate that PGC1alpha co-activation of HNF4alpha occurs on the PEPCK promoter. S/he also cautions against conclusions drawn from samples collected after isofluorane euthanasia. Finally, s/he is concerned that the echo tracing data depicted in Fig. 15 does

not fully support the conclusion that 3-MP-mediated gluconeogenesis suppression reverses ventricular chamber dilation and cardiac dysfunction. This Reviewer also lists several other items of concern that require your action.

Reviewer 3 would like to see a detailed and convincing discussion on how elevated glucose levels actually lead to cardiac effects. S/he also lists a few other issues that require your intervention.

As mentioned above, although the Reviewers 1 and 2 are generally positive, the issues raised by Reviewer 2 are of a fundamental nature. Unfortunately, editorial decisions cannot be made on the basis of a majority vote but must take into consideration the specific merits and quality of each evaluation.

Considered all the above, while publication of the paper cannot be considered at this stage, we would be prepared to consider a suitably revised submission, with the understanding that the Reviewers' concerns must be fully addressed with additional experimental data where appropriate and that acceptance of the manuscript will entail a second round of review.

Please note that it is EMBO Molecular Medicine policy to allow a single round of revision only and that, therefore, acceptance or rejection of the manuscript will depend on the completeness of your responses included in the next, final version of the manuscript.

Since the required revision in this case appears to require a significant amount of time, additional work and experimentation and might be technically challenging, I would therefore understand if you chose to rather seek publication elsewhere at this stage. Should you do so, we would welcome a message to this effect.

As you know, EMBO Molecular Medicine has a "scooping protection" policy, whereby similar findings that are published by others during review or revision are not a criterion for rejection. However, I do ask you to get in touch with us after three months if you have not completed your revision, to update us on the status. Please also contact us as soon as possible if similar work is published elsewhere.

I look forward to seeing a revised form of your manuscript as soon as possible.

***** Reviewer's comments *****

Referee #1 (Comments on Novelty/Model System):

The study demonstrates a surprising and novel link between the genetic heart disease and the liver through lipid and exacerbation of cardiac dysfunction in HCM by the hepatic response. The conclusions of this study are generally well supported by abundant data and the data convincing. I have some suggestions for the authors. Most of them are to increase the clarity of experimental details since the manuscript is difficult to read at the present form possibly due to the fact that too many data are presented in a short paper format.

Referee #1 (Remarks):

This is an interesting study showing that AMPK and CD36 activity are decreased in male HCM hearts, which in turn induces accumulation of triglyceride, DAG and oleic acid in the liver and upregulates PEPCK, an enzyme to stimulate gluconeogenesis. Suppression of PEPCK by 3-MPA rescued cardiac dysfunction. The study demonstrates a surprising link between the genetic heart disease and the liver through lipid and exacerbation of cardiac dysfunction in HCM by the hepatic response. The conclusions of this study are generally well supported by abundant data and the data convincing.

General:

The authors show that the mice develop cardiac dysfunction between 5.6 and 6 months in supplemental Figure 4A and the EF remains depressed thereafter. In page 4, lines 20-22, the authors stated that cardiac dysfunction is less prominent at 6 months, which provides an impression that metabolic changes precede cardiac dysfunction. It is unclear to this reviewer exactly when the decrease in AMPK in the heart and accumulation of lipid in the liver began to be seen and what their cause-effect relation with cardiac dysfunction in male HCM mice. In Figure 6E, neither PKC nor p38 was affected at 6 months when the animals have already shown decreases in LVEF.
In supplemental Figure 16, the authors could propose hypothetical mechanisms regarding how AMPK and CD36 are specifically downregulated in the genetic model but not other models of heart failure.

The authors should clarify exactly when 3-MPA is given. Is it given after PEPCK is activated and heart failure has been developed? If so, did it reverse heart failure?

This paper has many abbreviations and the data is presented without rationales or details of experimental procedures. For example, explanations regarding GSV and WR1399 would be necessary in the text to explain Figure 1. What is "minutes post-injection" in Figure 2G? No explanation is found in the Text regarding cellular fatty acid composition in Figures 5 and 6.

Specific

Please clarify exactly when pAMPK was evaluated in Figure 3A. Inactivation of AMPK is unclear in supplemental Figure 4D. It would be nice if the authors show the time course of AMPK in activation in the HCM mice.

In Figure 5A, the difference between WT and HCM is unclear. No explanation is given in the Text regarding Figure 5B.

Referee #2 (Comments on Novelty/Model System):

This manuscript addresses an interesting and important issue that, to date, has not received much attention: the culmination of metabolic changes in familial hypertrophic cardiomyopathy compared to pressure-overload induced cardiac hypertrophy. Hence this study has the potential to impact cardiovascular and metabolism research. If considered for resubmission this manuscript requires major revisions and the use of more appropriate metabolic methods in order to correctly investigate cardiac and systemic metabolism in HCM. Moreover, stylistically, the overwhelming and often confusing presentation of such a large dataset necessitates better organization.

Referee #2 (Remarks):

The authors report that HCM, caused by mutant -MHC expression in the heart, leads to systemic metabolic perturbations, which ultimately negatively impacts cardiac function in males. They provide data that attempt to support the notion that the heart alters hepatic glucose output by modulation of VLDL-triglyceride clearance. The authors suggest that restoring systemic metabolism, specifically restoring gluconeogenesis, rescues cardiac dysfunction in this mouse model of HCM.

The cardiac metabolic differences between the HCM model investigated here and pressure-overload is an important distinction that warrants investigation. Relatively little is known about the culmination of systemic- and cardio-metabolic changes in familial HCM, [although a recent publication by Wu et.al. (Exp Phsiol 2012) has suggested that familial HCM caused by troponin I mutations results in metabolic consequences due to altered calcium sensitivity]. Hence this study has the potential to impact cardiovascular and metabolism research.

While the question posed is interesting and potentially informative, more direct experimental evidence is necessary to support the conclusions. The concept of a cardiac-hepatic axis of fuel

partitioning is compelling, but the studies as presented are correlative, and as such, the broad conclusions seem premature and a bit over-reaching. The authors need to perform standard metabolic experiments in order to directly investigate the metabolic cause and consequences of HCM. Questions regarding methods, data clarification, and experimental suggestions are listed below.

Major points (in order of occurrence):

1. One main finding of this study (that in HCM, decreased LV function is associated with decreased AMPK activity and consequently decreased ATP in the heart, and that this can be reversed by AMPK agonist or by improving lipid delivery in the heart) is concluded entirely on indirect experimental results. The reasoning for experiments and the interpretation of data sets is not sound based on the following points.
a. A key issue in metabolism in general is alterations in flux through metabolic pathways. Although technically difficult, metabolic tracers must be used to determine substrate uptake and oxidation. Unfortunately, heart perfusions are the only experiments that can determine metabolic flux through the heart.
b. While metabolic changes may occur under specific settings, built-in mechanisms exist to maintain metabolic homeostasis within the cell. Although cardiomyopathies present with impaired energy transfer, which can result in cardiac dysfunction, the creatine/phosphocreatine system helps to minimize a decrease in [ATP]. The intracellular concentration of ATP in the myocyte is relatively low, the turnover rate of ATP is high, and the stability of ATP is low, thus it is difficult to make energetic conclusions based on ATP measurements from heart tissue. If the authors wish to investigate [ATP], phospho-NMR should be used. (See Beer et al, 2002; Lopaschuk et al, 2010; Balaban et al Am J Physiol Cell Physiol 2011)
c. Because the heart has such a high energy demand, it will use whatever substrates are available to meet that demand. Thus, AMPK is important in this process. AMPK phosphorylation can be affected by many different stimuli, including brief ischemia and even contraction; it can only be a marker of what is happening in the cell. This is another reason cardiac metabolism needs to be assessed in real time by metabolic tracers.
d. Although beta-oxidation of fatty acids generates ATP more efficiently, enhanced glucose oxidation in the heart can generate ATP in a compensatory manner. Kuang et al (Circulation 2004) demonstrated that loss of CD36 in the heart does not lead to energetically compromised hearts, as these hearts displayed increased generation of glucose-derived ATP. Thus, decreased levels of CD36 in the HCM heart does not necessarily result in decreased ATP generation.

2. Figure 1: the supposition that altered cardiac lipid uptake occurs because mRNA levels were reduced in HCM hearts is weak. (See reasoning in point 1). Also, it is not clear how CD36 activity was determined in figure 1C.

3. Triton WR1339 is a lipoprotein lipase inhibitor and would be expected to inhibit lipolysis rather than secretion of lipoproteins.

4. Figure 3: It is well established that AICAR is an AMPK agonist; however AICAR is an AMP-analog (ZMP). Thus, it is difficult to specifically conclude that activation of AMPK is what drives the changes presented in this figure. Other kinases and enzymes are also responsive to alterations in the AMP:ATP ratio within the cell, therefore these results should be interpreted with caution. Also, although statistically significant, the magnitude of changes in EF does not appear to be physiologically significant.

5. Does AICAR rescue the cardiac gene expression or NEFA release defects presented in Fig1?

6. What is the effect of HCM on the heaptic SREBP1c pathway? Plasma parameters such as insulin, glucagon, corticosterone would be informative.

7. Oleic acid is generally considered protective due to activation of the nuclear receptor PPARa, which would result in improved systemic lipid parameters. Please address this.

8. Regarding figure 4 and supplemental figure 6: can the authors explain why hepatic FFA and DAG seem to be decreased at 6 months but increased at 12 months in HCM? Also, how do these in vivo data compare to experiments performed in hepatocytes in vitro? Hepatocytes seem to accumulate TG in a short period of time compared to the 6 and 12 month in vivo data.

9. Blood is complex and may contain other things that alter FaO cell responses to its application. Experiments should be done following extraction of the plasma to enrich the lipid species away from proteins, etc, and could be performed with individual fatty acids loaded onto BSA as controls.

10. Evidence for enhanced hepatic glucose output is lacking. Increased expression of hepatic PEPCK mRNA is not a sufficient measure to indicate that liver is the source of the subtle increase in plasma glucose. Indeed, this minor increase may be related to decreased insulin secretion from the pancreas, and/or decreased glucose clearance by other tissues such as skeletal muscle or fat, all of which are expected to occur with pathological elevation in circulating fatty acids. Hyperinsulinemic-euglycemic clamp studies would be the most rigorous approach to address the cause(s) of increased plasma glucose, whether due to enhanced hepatic glucose production or decreased glucose disposal.

11. Is the timecourse (x-axis) for the pyruvate tolerance test correct as presented in Fig7G?

12. The evidence for PGC1 coactivation of HNF4 on the PEPCK promoter is weak. The modest increases in mRNA and protein do not imply activation of this transcription factor's (TF) activity. Further, the lack of change in the expression level (mRNA or protein) of any TF does not sufficiently rule out a role in modulation of downstream target genes. Indeed, many TFs expressed at steady levels are known to cooperate with PGC1 on the PEPCK promoter. Tandem ChIP experiments would address this (PGC1 followed by HNF4a, FOXO1, CREB, GR, or PPAR , or vice-versa). ChIPs should be quantitated by QPCR, not an end-point gel. ChIP primers sequences should be reported in methods.

13. The method of euthanasia (isofluorane) is known to increase plasma glucose, catecholamines, and glucocorticoids. Therefore, interpretation of data from samples collected in such a way is inconclusive. Time of day of sampling will also impact interpretation. This is evident in the assessment of GR-responsive genes in the liver as well as catecholamine measures. Comparison of fed vs fasted state in the wildtype animals would serve as positive controls in these cases. The lack of observable effects limits the utility of these data for ruling out effects of GR or catecholamines.

14. The authors suggest that 3-MPA-mediated suppression of gluconeogenesis reverses ventricular chamber dilation and cardiac dysfunction. In supplemental figure 15 the representative picture of echo tracing data is not convincing. From what little tracing is shown, the HCM heart post-MPA treatment appears to have decreased heart rate, which may not give accurate diastolic LV volume calculations (figure 8C). As mentioned above, slight changes in EF may not be physiologically relevant. It is surprising that HCM hearts have such "normal" EF.

15. Please discuss systemic AMPK agonism or PEPCK inhibition as it relates to a heart-autonomous effect or pleiotropic effects throughout the body on the improved outcomes observed.

16. Supp15a,b... why no rescue compared to fig8

17. Are the TAC models the same age as HCM mice? Inappropriate comparison if not.

18. Overall, the data presentation was challenging to follow. The authors should more clearly describe the age of the animals for all experiments.

Minor points (in order of occurrence):

1. Please specify diet source and number. "Standard" rodent chow compositions vary dramatically and impact all parameters tested.

2. Specify times of day for blood and tissue collections as well as fed/fasted conditions since all plasma and gene expression parameters show circadian variation. Moreover, comparison between experiments is only valid when sampled at a similar time of day under similar feeding states.

3. Please clarify the method of blood and tissue collection. Why were the animals perfused with PBS? It seems this would impact accurate analyses.

4. Please clarify the age of mice in the following figures: supplemental figure 1E, figure 2F-G, figure 3A-C, supplemental figure 3D-E, supplemental figure 12 and 13.

5. Please show B-tubulin as a loading control for all western blots. Also, please specify if the quantitation is based upon densitometry from pooled samples run in a single lane, or are averaged from individual samples run in separate lanes.

6. The western blot image in Fig1c appears to show different band sizes for CD36. Is this expected or an artifact of cut-and-paste from different lanes of the blot? Please address.

7. It is unclear how the TLC assays are quantitated. Please specify in methods.

8. Figure 2: Fasting state increases VLDL release from liver so not necessarily measuring basal increased levels of VLDL TG due to decreased lipid uptake/clearance by the heart (again, this needs to be measured in isolated heart perfusions). Please specify what time of day the experiments were performed as TG levels are also rhythmically regulated.

9. Figure 5B: What is the expression of PEPCK in this experiment?

10. Figure 6f: Why do cultured hepatocytes require overexpression of PGC1a in order to see upregulated gene expression (PEPCK)? As mentioned above, is this due to the short-term in vitro time frame?

Referee #3 (Comments on Novelty/Model System):

What seems to be missing from the study is a clear explanation of why elevated glucose resulting from liver dysfunction results in damages to the heart in HCM mice.

Referee #3 (Remarks):

Overall, this is an interesting study that correlates a cardiac defect with aberrant lipid accumulation in the liver. The investigators conclude from their study that impaired triglyceride metabolism in the heart leads to aberrant lipid accumulation and signaling in the liver resulting in an increase in blood glucose levels which is assumed to result in damage to the heart. If true, this is a potentially important study. What seems to be missing from the study is an understanding of how elevated glucose levels lead to cardiac defects in the HCM mouse. For example, elevated plasma TG normally do not result in such a dramatic phenotype. The authors should address this in their Discussion.

In addition, several issues need to be addressed:

1. Figure 1 - LpL activity should be directly measured (or immunoblot results shown). The levels of the VLDLr should also be quantified by immunoblot analysis

2. How were the levels of TG quantified in Fig 5A. The bar graph seems to exaggerate the levels.

3. How specific is 3-MPA

1st Revision - authors' response 03 September 2013

Referee #1 (Remarks):

This is an interesting study showing that AMPK and CD36 activity are decreased in male HCM hearts, which in turn induces accumulation of triglyceride, DAG and oleic acid in the liver and upregulates PEPCK, an enzyme to stimulate gluconeogenesis. Suppression of PEPCK by 3-MPA rescued cardiac dysfunction. The study demonstrates a surprising link between the genetic heart disease and the liver through lipid and exacerbation of cardiac dysfunction in HCM by the hepatic response. The conclusions of this study are generally well supported by abundant data and the data convincing.
General:
The authors show that the mice develop cardiac dysfunction between 5.6 and 6 months in supplemental Figure 4A and the EF remains depressed thereafter. In page 4, lines 20-22, the authors stated that cardiac dysfunction is less prominent at 6 months, which provides an impression that metabolic changes precede cardiac dysfunction. It is unclear to this reviewer exactly when the decrease in AMPK in the heart and accumulation of lipid in the liver began to be seen and what their cause-effect relation with cardiac dysfunction in male HCM mice.

Although less prominent [than later time-points], cardiac dysfunction first appears at 6 months. 6 months proves to be a pivotal time-point in this model, as the appearance of dysfunction/dilation coincides with the earliest signs of metabolic abnormalities (e.g. reduced CD36 expression, diminishing AMPK activity and increase in plasma TG). We posit that the expression of a mutated alpha-myosin transgene initiates pathological ventricular hypertrophy and that sexually dimorphic disease modifiers (e.g. estrogenic effects on AMPK) direct the outcome; the maintenance of hypertrophy or progression to failure. Substantial accumulation of lipids in the liver is secondary to the metabolic changes observed in connection to dysfunction of the ventricle. Therefore, rectifying the primary metabolic aberrations of the heart (i.e. AMPK inactivity) or secondary hepatic response (i.e. excessive glucose production) pushes the heart back into a compensated hypertrophic state.

In Figure 6E, neither PKC nor p38 was affected at 6 months when the animals have already shown decreases in LVEF.

We make the point that PKC and MAPK are not yet activated because there is insufficient hepatic lipid accumulation at 6 months. We now have data that the level of PKC/MAPK activity is a function of lipid accumulation in the liver (Figure6E,G; Supp Fig 9A-C; Supp Fig 10G,J).

In supplemental Figure 16, the authors could propose hypothetical mechanisms

regarding how AMPK and CD36 are specifically downregulated in the genetic model but not other models of heart failure.

The loss of CD36 expression and fatty acid-uptake activity in the hearts of HCM subjects is well documented [Tanaka, JMCC, 1997; Okamoto, Jpn Circ J., 1998]. The reason for the change in CD36 expression remains unknown. However, we have found (1) decreases in FOXO1/AMPK levels was associated with a loss of CD36 expression in the HCM heart, (2) an increase in FOXO1/AMPK levels was associated with increased CD36 expression in the pressure-overloaded heart and (3) that CD36 downregulation preceded that of other PPAR targets. These observations suggest that the loss of FOXO1, a transcriptional regulator of CD36 [Bastie, JBC, 2005], contributes to the HCM-associated decrease in CD36, but not other etiologies of heart failure. The FOXO1 data for HCM and banded hearts has been added to the manuscript (Supp Figure6A, H). Furthermore, free fatty acids and estrogen activate AMPK, independently of changes in adenine nucleotides, pCr and creatine content [Clark, Eur. J. Biochem., 2004; Rogers, Biochem Biophy Res Comm, 2009; D'Eon, Obesity, 2008]. While free fatty acid and triglyceride levels decrease in the HCM heart, ventricular lipid levels increase in the hearts of banded mice. Such cumulative differences could account for the discrepancy between heart failure models. And the fact that females do not display a pathological loss of CD36 or AMPK activities, conforms to a large body of evidence that higher levels of circulating estrogen in females is cardioprotective.

The authors should clarify exactly when 3-MPA is given. Is it given after PEPCK is activated and heart failure has been developed? If so, did it reverse heart failure?
3-MPA was administered to male mice at 15-18 months of age. Our echocardiography data show that the HCM controls (administered vehicle) are indeed in heart failure (e.g. dilation and dysfunction). We also show that excessive PEPCK activity is present at this time and that 3-MPA reverse both elevated glucose and ventricular dysfunction. The above data are in figures 8B,D,E.

This paper has many abbreviations and the data is presented without rationales or details of experimental procedures. For example, explanations regarding GSV and WR1399 would be necessary in the text to explain Figure 1. What is "minutes post-injection" in Figure 2G?

Triton is described (in figure legends 1E and 2E) as an inhibitor of lipolysis (for the measurement of hepatic triglyceride secretion). "Minutes post-injection" refers to the triton WR1339 time-course. We have reduced the number of abbreviations, for instance "GSV" has now been simply listed as "intracellular" (or IC), and experiments prefaced with rationale.

No explanation is found in the Text regarding cellular fatty acid composition in Figures 5 and 6.

The figures are referenced in the text (below), but we have now highlighted the importance of oleate accumulation and have added numerous regression analyses for emphasis (Fig 7B).
The Results section currently states: "In addition to TG accumulation, both the VLDL fraction and hepatocytes cultured with plasma from 12 month old HCM mice displayed increased levels of oleic acid, representing an overlapping lipid signature between circulating lipoproteins and hepatocytes cultured with HCM plasma (Figure 5C-E)… Comparable to the circulating VLDL fatty acid profile, we found oleic acid enrichment in the TG, diacylglycerol and free fatty acid fractions of the end-stage HCM liver (Figure 6A, Supporting Information Figure 10A)."
And the Discussion section states: "Increased TG and oleic acid in the VLDL fraction, which we posit is the result of a decreased capacity for cardiac lipid disposal, accumulates in the HCM liver."

Specific
Please clarify exactly when pAMPK was evaluated in Figure 3A. Inactivation of AMPK is unclear in supplemental Figure 4D.

pAMPK in Figure 3a was evaluated at 12 months of age, and the legend has been amended to reflect this oversight. ACC (Supp Figure 7D) is a target of AMPK and the representative pACC band is 51% lighter in HCM (once normalized to total ACC), and was measured by densitometry.

It would be nice if the authors show the time course of AMPK inactivation in the HCM mice.

We have now examined time-points earlier than 6 months of age and found that AMPK phosphorylation is unchanged in the HCM ventricles before 6 months of age. The progressive reduction in AMPK phosphorylation from 66% ($p= 0.088$) to 78% ($p=0.0001$) between 6 and 12 months of age show that 6 months is the earliest decrease in AMPK activity. These data are now presented in Supp Figure 7B-C.

In Figure 5A (FaO TLC), the difference between WT and HCM is unclear. No explanation is given in the Text regarding Figure 5B.

The large file size required compression for ease of uploading, so differences in band density may be poorly represented. This will be remedied when the uncompressed file is uploaded. We have added quantitative data using an enzymatic/colorimetric assay, matching the data gleaned from TLC, now in Supp

figure 9F-G.
Figure 5B shows qPCR data of lipogenic gene expression in plasma-culture FaO cells. The text references Fig5B as: "Consistent with our observations *in vivo,* hepatocytes cultured with HCM plasma accumulated TG and fatty acids, **in the absence of lipogenic gene activation** and cholesterol buildup"

Referee #2 (Remarks):

Major points (in order of occurrence):
1. One main finding of this study (that in HCM, decreased LV function is associated with decreased AMPK activity and consequently decreased ATP in the heart, and that this can be reversed by AMPK agonist or by improving lipid delivery in the heart) is concluded entirely on indirect experimental results. The reasoning for experiments and the interpretation of data sets is not sound based on the following points.

a. A key issue in metabolism in general is alterations in flux through metabolic pathways. Although technically difficult, metabolic tracers must be used to determine substrate uptake and oxidation. Unfortunately, heart perfusions are the only experiments that can determine metabolic flux through the heart.

Showing reduced lipid oxidation by the HCM heart was not our aim, as this has been demonstrated repeatedly in the literature. Rather, we want to show that the ventricular capacity for triglyceride hydrolysis (via lipoprotein lipase) and fatty acid uptake (via CD36) are impaired. Our *in vitro* assay demonstrates quite well that lipase-dependent triglyceride hydrolysis is reduced in the HCM heart and avoids the ventricular perfusion deficiencies observed in this mouse model [Olsson, AJP-Hrt Circ Phys, 2001], familial HCM patients [Grover-McKay, JACC, 1989] and inferred by our unpublished finding that the expression of the endothelial cell marker, CD31, is significantly reduced in the HCM heart. Although the use of heparin (as an anti-coagulant) in heart perfusion experiments is important, it would result in the cleavage of heparan sulfate and release of lipoprotein lipase into the vascular lumen, further confounding any lipase activity gleaned from such an experiment.
It is well known that the HCM heart does not clear fatty acids efficiently [Tadamura, J Nucl Med, 1997; Takeishi, Eur J Nuc Med, 1992; Tanaka, JMCC, 1997; Watanabe, Ann Nuc Med, 1998], resulting in reduced myocardial triglyceride content [Nakae, J Cardiac Failure, 2010]. The loss of myocardial triglyceride content in HCM is important as it is correlated with contractile dysfunction and contrasts the increased triglycerides observed in the ischemic heart [Nakae, J Card Fail, 2010]. So, after demonstrating that the murine HCM heart also displays reduced lipolytic activity and lipid content, we tasked ourselves with uncovering the cause(s) of this pathological metabolic phenotype and the impact of correcting these perturbations.

b. While metabolic changes may occur under specific settings, built-in mechanisms exist to maintain metabolic homeostasis within the cell. Although cardiomyopathies present with impaired energy transfer, which can result in cardiac dysfunction, the creatine/phosphocreatine system helps to minimize a decrease in [ATP]. The intracellular concentration of ATP in the myocyte is relatively low, the turnover rate of ATP is high, and the stability of ATP is low, thus it is difficult to make energetic conclusions based on ATP measurements from heart tissue. If the authors wish to investigate [ATP], phospho-NMR should be used. (See Beer et al, 2002; Lopaschuk et al, 2010; Balaban et al Am J Physiol Cell Physiol 2011)

The luminescent assay used to measure ATP has been used in studies of the cardiomyocyte or cardiovascular cells [Sun, J. AHA, 2013; Bekeredjian, PLoS One, 2010; Kawano, Life Sci, 2012; Sharma, Free Rad Biol, 2012], as well as dozens of publications concentrating on other cells/organs, including Dvoriantchikova, Mol. Vis., 2010; Belleannée, AJP-Cell Phys, 2010; Ponnusamy, AJP-Ren Phys, 2011; Chandak, JBC, 2010; Chen, Biochem J, 2012; Suhr, PLoS One, 2010; Berman-Booty, Cancer Prev. Res., 2013; Sun, J. Am Heart Assoc., 2013; Ma, PLoS One, 2012.
Both phosphocreatine and ATP have been shown to be reduced in the human HCM heart [Beer, JACC, 2002; Starling, Mol. Cell. Biochem., 1998]. Therefore we wanted to determine whether there is a similar energetic deficit in the murine heart (with familial HCM) and if AICAR can rescue low ATP levels. We believe that our method is sufficient to demonstrate this well-characterized "energy starvation." While NMR would give us valuable inorganic phosphate and AMP measurements not provided by the employed assay, an endpoint measurement of ATP content by NMR will not change the turnover rate or stability of ATP in our frozen samples, a concern of this reviewer.

c. Because the heart has such a high energy demand, it will use whatever substrates are available to meet that demand. Thus, AMPK is important in this process. AMPK phosphorylation can be affected by many different stimuli, including brief ischemia and even contraction; it can only be a marker of what is happening in the cell. This is another reason cardiac metabolism needs to be assessed in real time by metabolic tracers.

AMPK is more than just a marker. AMPK is a well-established coordinator of cellular stimuli/response and regulator of cellular energy production/expenditure [Dolinsky and Dyck, AJP Hrt Circ Phys, 2006].
Our unpublished data showing myocardial insulin resistance indicates that the metabolic substrate flexibility of the HCM heart is substantially reduced, making insufficient AMPK activity during "energy starvation" that much more important. Metabolic tracers may tell us what is entering the myocardium, but nothing about the upstream components regulating lipid clearance (e.g. AMPK and CD36). As

deficiencies in fatty acid analog uptake by the hypertrophic cardiomyopathic heart have been repeatedly demonstrated [Tadamura, J Nucl Med, 1997; Takeishi, Eur J Nuc Med, 1992; Tanaka, JMCC, 1997; Watanabe, Ann Nuc Med, 1998], we aimed to identify, study and rectify upstream regulators of lipid clearance (e.g. AMPK-mediated CD36 activity).

d. Although beta-oxidation of fatty acids generates ATP more efficiently, enhanced glucose oxidation in the heart can generate ATP in a compensatory manner. Kuang et al (Circulation 2004) demonstrated that loss of CD36 in the heart does not lead to energetically compromised hearts, as these hearts displayed increased generation of glucose-derived ATP. Thus, decreased levels of CD36 in the HCM heart does not necessarily result in decreased ATP generation.

The earlier study by Irie et al. (PNAS 2003), showing decreased ATP content and cardiac output with CD36 deficiency, is far more informative than that of Kuang et al. Unlike the isolated measurement of a post-ischemic state (in the presence of fatty acids) by Kuang et al., Irie and colleagues measured ATP content both before and after ischemia in the presence and absence of fatty acids in the perfusion medium. Importantly, Irie and colleagues only observed the ATP deficit at baseline (before ischemia), in the absence of exogenous fatty acids in the perfusion medium. These points are very important because, 1) ATP depletion appears dependent upon a lack of free fatty acids in the perfusion medium (inferring compensation by other lipid uptake mechanisms in the Kuang study), 2) there is a shift to glucose utilization during ischemia, making the CD36-transported energetic substrates dispensable, 3) the Kuang and Irie studies looked at CD36 deficiency alone, rather than a combined CD36/LpL/FABP/VLDLR depletion, as we observed in HCM, 4) ischemia is not apparent in the HCM myocardium. Furthermore, HCM/DCM patient studies present reduced myocardial pCr and ATP [Beer, JACC, 2002; Starling, Mol. Cell. Biochem., 1998], a state thought to be conferred by mutations in sarcomeric proteins [Ashrafian, TRENDS Gen., 2003]. We do not suggest that ATP depletion is due to CD36 alone. It was our intention to show that reduced CD36 expression and activity merely contribute to the "energy starved" state of HCM (initiated by a mutant myosin), which is ameliorated by the AMPK agonist, AICAR. The importance of reactivating CD36 is underscored by our unpublished findings that the HCM heart is insulin resistant, displaying reduced insulin-induced AKT and glucose transporter-4 activity, and therefore less capable of taking up or metabolizing glucose.

2. Figure 1: the supposition that altered cardiac lipid uptake occurs because mRNA levels were reduced in HCM hearts is weak. (See reasoning in point 1).

The reviewer references only the first panel of Figure 1. We then go on to show

CD36 protein levels and activity (Figure 1B-C), as well as and LpL activity (Figure 1D-E, Supp Fig 2) and reduced myocardial lipid content (Figure 1F-G). We have added data showing that the administration of an AMPK agonist increases cardiac CD36 activity and oleic acid content, which are decreased in the HCM heart but elevated in plasma at baseline (Figures 2C, 3D-E,4E).

Also, it is not clear how CD36 activity was determined in figure 1C.

Measuring the ratio of CD36 protein at the plasma membrane versus intracellular vesicles is a well-established means of determining CD36 activity [Bonen, JBC, 2000; Luiken, Diabetes, 2003]. We employed a sucrose gradient-based fractionation method employed by the aforementioned authors (described in the Methods and supplemental methods sections).

3. Triton WR1339 is a lipoprotein lipase inhibitor and would be expected to inhibit lipolysis rather than secretion of lipoproteins.

We agree fully with the reviewer. The general use of Triton WR1339 is therefore to gauge the secretion rate of triglyceride-rich lipoproteins (from the liver) by inhibiting systemic lipolysis, which was how we employed it in the manuscript. The legend for figure 2E reads: "**(E)** Measurement of TG secretion in plasma and VLDL following inhibition of peripheral lipolysis by Triton WR1339 administration."

4. Figure 3: It is well established that AICAR is an AMPK agonist; however AICAR is an AMP-analog (ZMP). Thus, it is difficult to specifically conclude that activation of AMPK is what drives the changes presented in this figure. Other kinases and enzymes are also responsive to alterations in the AMP:ATP ratio within the cell, therefore these results should be interpreted with caution. Also, although statistically significant, the magnitude of changes in EF does not appear to be physiologically significant.

Most kinases/enzymes that respond to ZMP or AMP do so in an AMPK-dependent manner. With respect to the activation of glucokinase or phosphofructokinase, one would expect a change in intracellular glucose or glycogen content with AICAR administration; however no such changes were observed (Supp Figure 8I, and unreported data that we can add upon request). In fact, AICAR is a far more specific AMPK agonist, when compared to Metformin, which acts upon AMPK through its inhibition of the electron transport chain and ATP synthesis to decrease cellular ATP levels. If other enzymes that we are not aware of are ZMP-responsive, we would be happy to test their activities.
(Magnitude of EF change is also addressed for point #14);
A ~12% reduction in function was observed in (12-15 month old) HCM males. Similarly, human patients with HCM display a 6.1%/year decrease in ejection

fraction as they enter “end-stage” HCM [Harris, Circ., 2006]. Despite this small depression in function, it took 14 years for the “end-stage” phenotype in patients to be recognized (from the onset of HCM symptoms), and only 2.7 years from “end-stage” recognition to death or transplantation [Harris, Circ., 2006]. These findings underscore the importance of studying “end-stage” HCM at an early-to-middle stage. Most importantly, elevated VLDL-TG preceded a reduction in EF by almost 2 weeks, signifying a putative marker for the risk of displaying the “end-stage” phenotype resulting from hypertrophic cardiomyopathy (Figure 2B).

5. Does AICAR rescue the cardiac gene expression or NEFA release defects presented in Fig1?

AICAR increases CD36 levels and reduces elevated β-myosin and ANP expression (Figure 3D,G,H). We did not measure whether AICAR affects the gene expression profile in Figure1A, but would be happy to if requested. AICAR was not expected to rescue the NEFA release defect as triglyceride hydrolysis is a function of lipoprotein lipase activity.

6. What is the effect of HCM on the heaptic SREBP1c pathway? Plasma parameters such as insulin, glucagon, corticosterone would be informative.

Both plasma insulin and hepatic SREBP-1 expression were elevated, but not included in the manuscript. Although plasma insulin was elevated, we observed normal hepatic insulin sensitivity (with respect to AKT phosphorylation and regulation of gene expression), upon the administration of exogenous insulin (not in the manuscript). We decided not to pursue SREBP-1 activity since many of its target genes (e.g. fatty acid synthase, GPAT) were not activated in the liver. Glucagon/receptor activates PEPCK expression via direct binding of the *Pepck* promoter by CREB or the transcription activation of *Pgc1* by CREB. We observed significant reductions in CREB phosphorylation (Supp Fig 13A) and no in Pgc1 transcript levels (Fig 7C). We have added chromatin immunoprecipitation data showing reduced binding to the *Pepck* promoter by CREB in HCM livers (Supp Fig 13B). Therefore, we did not pursue plasma glucagon as an essential component of the HCM phenotype.

7. Oleic acid is generally considered protective due to activation of the nuclear receptor PPARa, which would result in improved systemic lipid parameters. Please address this.

Oleic acid is generally considered protective because it induces (highly bioactive) DAG and free fatty acid incorporation into (benign) triglyceride stores [Listenburger, PNAS, 2003]. Conversely, increased PPARa activity is a major driver of steatosis of the heart and liver, as well as insulin resistance, resulting in adverse cardiac remodeling and dysfunction [Park, Diabetes, 2005].

8. Regarding figure 4 and supplemental figure 6: can the authors explain why hepatic FFA and DAG seem to be decreased at 6 months but increased at 12 months in HCM? Also, how do these in vivo data compare to experiments performed in hepatocytes in vitro? Hepatocytes seem to accumulate TG in a short period of time compared to the 6 and 12 month in vivo data.

Only DAG was significantly reduced in the 6 month livers. The discrepancy between the 6 and 12 month livers is likely due to the amount of time required for lipids accumulating in the plasma to accumulate in the liver and the systemic complexity of *in vivo* vs. *in vitro* experiments. Elevated plasma VLDL-TG only appear less than 2 weeks before the 6 month time point and should take more time to amass to a measurable extent. This may be explained by the abilities of adipose to buffer changes in plasma TG levels and the liver to repackage and secrete increased lipids. Plasma-cultured cells accumulated lipids in a concentration-dependent manner, such that TG-rich 6 month plasma also resulted in increased cellular TG content (not in the manuscript).

9. Blood is complex and may contain other things that alter FaO cell responses to its application. Experiments should be done following extraction of the plasma to enrich the lipid species away from proteins, etc, and could be performed with individual fatty acids loaded onto BSA as controls.

We did such experiments and VLDL lipid extracts activate p38 MAPK (Fig 6I) and isolated VLDL fractions (containing only associated apolipoproteins) activate PEPCK expression in vitro, in an oleic acid-dependent manner (Fig 7A-B). We were concerned that the administration of BSA-complexed to free fatty acids would not be an appropriate representation of the circulating gluconeogenic stimulus since the *in vivo* and *in vitro* phenotypes appear to be a response to elevated (oleate in) VLDL-triglyceride, rather than a simple increase in plasma free fatty acids (Fig2A,B,D; Fig 6J, Fig 7C,I; Supp Fig. 3C).

10. Evidence for enhanced hepatic glucose output is lacking. Increased expression of hepatic PEPCK mRNA is not a sufficient measure to indicate that liver is the source of the subtle increase in plasma glucose. Indeed, this minor increase may be related to decreased insulin secretion from the pancreas, and/or decreased glucose clearance by other tissues such as skeletal muscle or fat, all of which are expected to occur with pathological elevation in circulating fatty acids. Hyperinsulinemic-euglycemic clamp studies would be the most rigorous approach to address the cause(s) of increased plasma glucose, whether due to enhanced hepatic glucose production or decreased glucose disposal.

In addition to Pepck expression coupling quite well with PEPCK activity, hepatic

glucose production and blood glucose levels throughout the manuscript and the literature [Rognstad, JBC, 1979; Gomez-Valades, Mol. Ther., 2006], there is a clear increase in pyruvate-derived glucose production (in the absence of anesthesia). We have ample evidence (not in the manuscript) of improved insulin sensitivity in the gastrocnemius muscle, elevated plasma insulin levels, normal glucose tolerance (in the absence of anesthesia) and hepatic insulin sensitivity, as well as increased systemic insulin-dependent glucose clearance in the HCM mouse. Importantly, we found that anesthetics (e.g. isoflurane) resulted in substantial glucose intolerance in the HCM, but not wildtype mice. These findings would confound the interpretation of hyperinsulinemic euglycemic clamp studies.
Furthermore, the manuscript shows that circulating fatty acids are NOT elevated in the HCM mouse.

11. Is the timecourse (x-axis) for the pyruvate tolerance test correct as presented in Fig7G?

Yes.

12. The evidence for PGC1; coactivation of HNF4; on the PEPCK promoter is weak. The modest increases in mRNA and protein do not imply activation of this transcription factor's (TF) activity. Further, the lack of change in the expression level (mRNA or protein) of any TF does not sufficiently rule out a role in modulation of downstream target genes. Indeed, many TFs expressed at steady levels are known to cooperate with PGC1; on the PEPCK promoter. Tandem ChIP experiments would address this (PGC1; followed by HNF4a, FOXO1, CREB, GR, or PPAR;, or vice-versa). ChIPs should be quantitated by QPCR, not an end-point gel. ChIP primers sequences should be reported in methods.

PGC1 is not a transcription factor, as it does not have a DNA binding domain or bind DNA directly. Therefore, ChIP of PGC1 at the HNF4 binding (AF-1) site of *Pepck* promoter tells us that PGC-1 is bound to the *Pepck* promoter via an intermediary, DNA-bound protein (i.e. HNF4) [Herzog, Mol. Endocrin, 2004].
The lack of change in the expression level of Pgc1a transcript levels indicates that the observed increase in PGC-1 protein, phosphorylation and target expression are NOT a function of the transcriptional activation of *Pgc1* by activated CREB. This is supported by the significant reduction in CREB phosphorylation (Supp Fig 13A). We also observed a decrease in the amount of *Pepck* promoter pulled down with CREB (now Supp Fig 13B).
Tandem ChIP is not only rare, but incredibly difficult with tissues that contain low levels of the endogenous protein-of-interest (e.g. PGC-1). Therefore, *in vivo* tagged PGC1 overexpression would be required and likely change the hepatic lipid profile and PGC1 phosphorylation status or activity [Rhee, JBC, 2006].
ChIPs were quantified with semi-quantitative PCR and validated with qRT-PCR.

The gels shown were from PCR run for 20 cycles, hardly an endpoint.
Not including the ChIP primers in the methods was an oversight and now remedied.

13. The method of euthanasia (isofluorane) is known to increase plasma glucose, catecholamines, and glucocorticoids. Therefore, interpretation of data from samples collected in such a way is inconclusive. Time of day of sampling will also impact interpretation. This is evident in the assessment of GR-responsive genes in the liver as well as catecholamine measures. Comparison of fed vs fasted state in the wildtype animals would serve as positive controls in these cases. The lack of observable effects limits the utility of these data for ruling out effects of GR or catecholamines.

Fasting blood glucose levels were measured the exact same time of day (4pm) WITHOUT anesthesia. There was no increase in the expression of GR-responsive genes in the fasted or fed states (Supp Fig 13C). We actually observed a *decrease* in the levels of hepatic 11bHSD, the enzyme that converts local glucocorticoids to the active form, in the fed state.
ChIP of the PGC1-bound *Pepck* promoter had controls showing increased binding in the fasted state, reduced in the fed state in the WT liver (Supp Fig 12E). In contrast to other PGC-1 targets (e.g. CPT1a, UCP2), direct targets of GR were not activated. Plasma catecholamines were not only unchanged (Supp Fig3D), but the decrease in CREB phosphorylation and CREB bound to the *Pepck* promoter supports the lack of a role for catecholamines in the PEPCK phenotype.

14. The authors suggest that 3-MPA-mediated suppression of gluconeogenesis reverses ventricular chamber dilation and cardiac dysfunction. In supplemental figure 15 the representative picture of echo tracing data is not convincing. From what little tracing is shown, the HCM heart post-MPA treatment appears to have decreased heart rate, which may not give accurate diastolic LV volume calculations (figure 8C).

The echo tracing data pictures were low quality and removed. We have added data to the manuscript showing that there is NO change in the post-MPA HCM heart rate (compared to pre-MPA) (Supp Fig 17E).

As mentioned above, slight changes in EF may not be physiologically relevant. It is surprising that HCM hearts have such "normal" EF.

A ~12% reduction in function was observed in (12-15 month old) HCM males. Similarly, human patients with HCM display a 6.1%/year decrease in ejection fraction as they enter “end-stage” HCM [Harris, Circ., 2006]. Despite this small depression in function, it took 14 years for the “end-stage” phenotype in patients

to be recognized (from the onset of HCM symptoms), and only 2.7 years from “end-stage” recognition to death or transplantation [Harris, Circ., 2006]. These findings underscore the importance of studying “end-stage” HCM at an early-to-middle stage. Most importantly, elevated VLDL-TG preceded a reduction in EF by almost 2 weeks, signifying a putative marker for the risk of displaying the “end-stage” phenotype resulting from hypertrophic cardiomyopathy (Figure 2B).

15. Please discuss systemic AMPK agonism or PEPCK inhibition as it relates to a heart-autonomous effect or pleiotropic effects throughout the body on the improved outcomes observed.

Neither AICAR nor MPA significantly changes WT function (Figures 3I-J, 8D-E). PEPCK is not expressed in the heart.

16. Supp15a,b... why no rescue compared to fig8

The MPA administration period may have been too short or this phenotypic difference reflects the indirect nature of PEPCK inhibition. While AICAR was shown to directly activate ventricular AMPK, 3-MPA must act through the liver, as PEPCK is not expressed in the heart. However, we have added data showing that MPA favorably increases SERCA expression and decreases elevated monoacylglycerol acyltransferase-2 expression in the heart (Supp Fig 18C).

17. Are the TAC models the same age as HCM mice? Inappropriate comparison if not.

The TAC models were 6 months of age. Nevertheless, cardiac function and CD36 are still reduced and VLDL-TG elevated in 6 month old HCM mice, but NOT in the TAC mice. Banding older animals (16-18 months old) results in a very different phenotype; less cardiac fetal gene (e.g. beta myosin) expression, diminished myocardial hypertrophy, adverse coronary adaptation, aberrant proto-oncogene (Fos, myc) expression, and altered resistance to ischemia [Li, J. Gerontology, 2003; Isoyama, HF Rev., 2002]. Therefore, we thought it prudent to employ the conventional age for aortic constriction experiments.

18. Overall, the data presentation was challenging to follow. The authors should more clearly describe the age of the animals for all experiments.

We have restructured the manuscript Results section so that discussion of mice that were not 12 month old HCM males (e.g. young HCM males, females, and banded mice) are now isolated to their own sections and their respective ages better addressed throughout.

Minor points (in order of occurrence):

1. Please specify diet source and number. "Standard" rodent chow compositions vary dramatically and impact all parameters tested.
Teklad 8640 was used for every mouse in the study and has been added to the methods.

2. Specify times of day for blood and tissue collections as well as fed/fasted conditions since all plasma and gene expression parameters show circadian variation. Moreover, comparison between experiments is only valid when sampled at a similar time of day under similar feeding states.

Blood glucose and plasma were always sampled at 4pm, following a 6 hour fast and this information has been added to the Methods section. Exceptions include the Triton WR1339 and Pyruvate administration, which occurred after extended fasts (18hours).

3. Please clarify the method of blood and tissue collection. Why were the animals perfused with PBS? It seems this would impact accurate analyses.

The heart and liver hold a large volume of the blood supply at any given time. For instance, the liver contains approximately 15% of the body's blood content. Therefore, ice-cold PBS perfusion was used to exsanguinate the mice and initiate the preservation process, as unwanted blood cells and lipoproteins in the tissue would be worse for accurate tissue lipid and expression analyses.

4. Please clarify the age of mice in the following figures: supplemental figure 1E (TAC, 6 months), figure 2F-G (12-15 months)*, figure 3A-C* (12-15 months*), supplemental figure 3D-E* (12-15 months), *supplemental figure 12 and 13* (TAC, 6 months).

These ages have been added to the Methods and Figure Legends.

5. Please show B-tubulin as a loading control for all western blots. Also, please specify if the quantitation is based upon densitometry from pooled samples run in a single lane, or are averaged from individual samples run in separate lanes.

Tubulin could not always be used because of how the SDS-PAGE was run or the blot was cut. We will amend the manuscript to address sample state (i.e. pooled vs. individual).

6. The western blot image in Fig1c appears to show different sizes for CD36. Is this expected or an artifact of cut-and-paste from different lanes of the blot? Please address.

Insulin activity is known to attenuate, while fatty acids induce CD36 ubiquitination [Smith, JBC, 2008]. Therefore, a slight difference in the intracellular CD36 size in the WT heart is probably a function of the normal fatty acid-induced post-translational modifications of CD36. CD36 ubiquitination is expected to be reduced in the HCM heart, where there is reduced non-esterified fatty acid content (Figure 1G) and impaired insulin signaling (unpublished data).

7. It is unclear how the TLC assays are quantitated. Please specify in methods.

Densitometry and normalization to protein. We have amended the manuscript to explain the methodology.

8. Figure 2: Fasting state increases VLDL release from liver so not necessarily measuring basal increased levels of VLDL TG due to decreased lipid uptake/clearance by the heart (again, this needs to be measured in isolated heart perfusions). Please specify what time of day the experiments were performed as TG levels are also rhythmically regulated.

The fasting state was used for consistency with the conventions found in the literature and to reduce variability between different ages (2-18 months) and sexes. The fasted state increases VLDL secretion, in part, because non-esterified fatty acids are released by adipose tissue and cleared by the liver. Therefore, differences in VLDL secretion (between genotypes) would be expected if there was increased fatty acid release associated with HCM. However, we show that fasting non-esterified fatty acid levels were not different in the HCM mice. We measured all plasma parameters (VLDL TG and APOB as well as secretion by Triton WR1339) at the same time of day (4pm), under fasting conditions. This information has been added to the Methods.

9. Figure 5B (FaO TLC): What is the expression of PEPCK in this experiment?
10. Figure 6f: Why do cultured hepatocytes require overexpression of PGC1a in order to see upregulated gene expression (PEPCK)? As mentioned above, is this due to the short-term in vitro time frame?

FaO cells (also known as the H4IIE cells) were chosen because of their widespread use in the literature and their very low endogenous PGC1 expression [Herzog, Mol. Endocr., 2004]. Culturing FaO cells with HCM plasma in the absence of PGC1-overexpression did not yield an increase in PEPCK expression (Figure7A).

Referee #3 (Remarks):

Overall, this is an interesting study that correlates a cardiac defect with aberrant lipid accumulation in the liver. The investigators conclude from their study that impaired triglyceride metabolism in the heart leads to aberrant lipid accumulation and signaling in the liver resulting in an increase in blood glucose levels which is assumed to result in damage to the heart. If true, this is a potentially important study. What seems to be missing from the study is an understanding of how elevated glucose levels lead to cardiac defects in the HCM mouse. For example, elevated plasma TG normally do not result in such a dramatic phenotype. The authors should address this in their Discussion.

There are a number of studies that have demonstrated associations between elevated plasma triglycerides, plasma glucose and heart failure in the context of obesity in rodent models and human subjects [Zhou, PNAS, 2000; Hammer, JACC, 2008]. Others have shown that the correlation between increased circulating triglyceride and glucose in human subjects is independent of obesity or plasma free fatty acid levels and propose that increased VLDL triglyceride is a function of reduced peripheral lipase activity and clearance [Baldeweg, Eur. JCI, 2000]. Most importantly, triglyceride infusion has been shown to enhance hepatic glucose output in patients [Boden, JCI, 1994].
We have added data showing that there is an inverse relationship between ventricular function and fasting blood glucose levels (Fig 8A). However, the end-stage HCM phenotype appears to be unique and not a simple reflection of the "glucotoxic" components (e.g. oxidative stress, glycation events and glucose-sensitive gene expression) central to diabetic cardiomyopathy [Giacco and Brownlee, Circ Res, 2010]. We have added a considerable amount of data that shows reactive oxygen species, peroxidized lipids, glycogen storage, advanced glycated end-products, and most transcriptional targets upregulated by glucotoxicity are not elevated in the HCM heart (Supp Fig 16A-I). We have also added data regarding the effects of MPA-mediated lowering of blood glucose levels on glucose-regulated gene expression (Supp Fig 18C). Therefore, we concentrated on the hepatic components mediating and role of increased gluconeogenesis, and attempted to refrain from unsubstantiated speculation regarding a glucotoxic phenotype associated with HCM.

In addition, several issues need to be addressed:

1. Figure 1 - LpL activity should be directly measured (or immunoblot results shown). The levels of the VLDLr should also be quantified by immunoblot analysis

Western blots will not show active LpL that has appropriately migrated to the luminal surface of endothelial cells. Instead, we show reduced VLDL triglyceride hydrolysis, and that this was blunted with an inhibitor of LpL activity (Figure

1D,E). We did not stress a role for VLDLR as the data support impairment of VLDL triglyceride hydrolysis and fatty acid uptake, rather than uptake of whole VLDL particles.

2. How were the levels of TG quantified in Fig 5A. The bar graph seems to exaggerate the levels.

TG was quantified by densitometry of samples run on 2 TLC plates, and normalized to cell number. We have added quantitative data, matching the data gleaned from TLC (Supp figure9F-G), using an enzymatic/colorimetric assay.

3. How specific is 3-MPA

3-MPA is highly specific and results in the accumulation of the PEPCK substrate (oxaloacetate) and decrease in PEPCK product (phosphoenolpyruvate), resulting in the inhibition of hepatic glucose production and has been in use as an *in vitro* and *in vivo* PEPCK inhibitor for almost 40 years [Gomez-Valades, Mol. Therapy., 2005; Gstraunthaler and Handler, AJP-Cell Phys., 1987; Jomain-Baum, JBC, 1975; Yang, JBC, 2008].

2nd Editorial Decision 19 September 2013

Thank you for the submission of your manuscript to EMBO Molecular Medicine. We have now heard back from the two Reviewers, whom we asked to re-evaluate your manuscript.

You will see that while Reviewer 1 is now supportive, Reviewer 2 is still not satisfied that the issues raised were adequately addressed. Specifically, s/he notes that the sample collection methods do not allow for univocal interpretation of the data and maintains that other lipid-mediated pathways have been unduly ignored.

We have now re-discussed your manuscript in the light of these comments and in addition we have sought and obtained independent external advice from a leading expert. We agreed that the points raised by Reviewer 2 do have merits.

Although it is EMBO Molecular Medicine policy to allow a single round of revision only, I am prepared in this case, to allow you to submit a re-revised version that should at least provide sham controls for the methods of sample collection (e.g. anaesthesia, fasting) and an analysis of hepatic PPAR alpha activity. This should not prove overly demanding and would strengthen and consolidate your findings.

Acceptance or rejection of the manuscript will depend on the completeness of your responses included in the next, final version of the manuscript.

As you know, EMBO Molecular Medicine has a "scooping protection" policy, whereby similar findings that are published by others during review or revision are not a criterion for rejection. However, I do ask you to get in touch with us after three months if you have not completed your revision, to update us on the status. Please also contact us as soon as possible if similar work is published elsewhere.

I look forward to seeing a revised form of your manuscript as soon as possible.

***** Reviewer's comments *****

Referee #1 (Comments on Novelty/Model System):

The revised manuscript is improved and the authors clarified the issues I raised. This paper shows an unexpected link between HCM and the liver. Lipid accumulation of upregulation of gluconeogenesis in liver exacerbates cardiac dysfunction. Its translational value is very high.

Referee #1 (Remarks):

The authors addressed my concerns. The manuscript is improved signficantly.

Referee #2 (Remarks):

As stated in the first review of this manuscript, the authors present a compelling and important study describing the culmination of metabolic changes in a mouse model of familial hypertrophic cardiomyopathy. The resubmitted manuscript is improved in its organization, but still contains a somewhat overwhelming amount of data. While certain aspects of the first critique have been satisfactorily addressed, the conclusions regarding the mechanism of the poor systemic metabolic profile in the HCM model (ie, the hearts' reduced oleic acid uptake leading to hepatic accumulation and enhanced gluconeogenesis) continue to be premature. Namely, the methods of sample collection prevent the interpretation of data presented to rule out the roles of common stress pathways such as increased adrenergic tone and glucocorticoids (eg, use of anesthesia prior to euthanasia, fasting prior to catecholamine measures), and the involvement of other important lipid-mediated pathways is ignored (eg, PPAR alpha activity in the liver). Addressing these concerns would strengthen the merit of the authors' conclusions regarding a cardiac-hepatic axis. Therefore, it is difficult for this reviewer to advocate publication of this manuscript in its current form.

2nd Revision - authors' response 04 November 2013

Enclosed please find our revised manuscript (EMM-2013-02852) entitled "Metabolic Crosstalk between the Heart and Liver Impacts Familial Hypertrophic Cardiomyopathy." We are very pleased that Reviewers 1 and 3 were satisfied with our first revision. In our second revision, we have attempted to address every concern expressed by Reviewer 2. In doing so, we carried out many new experiments and placed some of these new data into in the manuscript but the rest of the data into a section following our responses, labeled "Response Figure X". {Removed upon Author request} We did this to mitigate a major expansion of the manuscript, which Reviewer 2 articulated as containing an "*overwhelming amount of data*". We have also appended a summary of all the experiments done in response to the two reviews.

1. Comments of Reviewer 2:
"…the methods of sample collection prevent the interpretation of data presented to rule out the roles of common stress pathways such as increased adrenergic tone and glucocorticoids (eg, use of anesthesia prior to euthanasia, fasting prior to catecholamine measures … provide sham controls for the methods of sample collection (e.g. anaesthesia, fasting)…"

A. Concerning euthanasia without anesthesia: This practice raises serious ethical issues pertaining to the conventions of the humane treatment of laboratory animals. In the literature, an un-anesthetized sampling state has been largely restricted to the non-terminal measurement of blood analytes (e.g. glucose), the effects of electrical stress, blood pressure, and magnetic resonance imaging. Furthermore, we are convinced that alternatives to un-anesthetized euthanasia would elicit a considerable stress response due to increased handling and restraint (for cervical dislocation, decapitation) or confounding hypoxic response (with asphyxiation).

B. To address the concern about the effects of anesthesia on circulating factors: Although every evaluation of the HCM phenotype in this manuscript has included age-matched non-transgenic (wildtype) littermate controls exposed to identical and concomitant handling procedures, we measured circulating metabolic factors in the presence and absence of anesthesia using male mice. The use of isoflurane anesthesia did not result in significant differences in circulating glucose, non-esterified fatty acids, immunoreactive insulin or glucose tolerance in these mice (Response Figure 1A-F) {Removed upon Author request}. Most importantly, the crux of the manuscript's thesis, that familial hypertrophic cardiomyopathy results in elevated glucose production and circulating glucose levels (Figure 7H, J), was demonstrated with sampling in the absence of anesthesia.

C. To address the concern about sampling after fasting: Fasted blood glucose and triglyceride measurements were used in order to reduce the variability

conferred by the natural circadian rhythms affected by feeding. Fasting and testing at approximately 16:00 hours, as employed by our study, avoids accentuating and sampling during the daily minima/maxima of phosphoenolpyruvate carboxykinase (PEPCK) activity in mice, respectively [Phillips and Berry, *AJP*, 1970]. Similarly, fasting minimizes the daily variations in hepatic VLDL triglyceride secretion and excludes the confounding contribution of intestinal chylomicron secretion [Cohn, *JCI*, 1990]. Nevertheless, in response to this concern, we measured plasma and liver norepinephrine content, total plasma and VLDL triglyceride levels, and plasma non-esterified fatty acid levels in fasted and *ad libitum* fed wildtype mice (Response Figure 2A-D) {Removed upon Author request}. As previous studies have shown, fasting reduced plasma norepinephrine levels and reduced both total and VLDL triglycerides, due to decreased intestinal contribution of chylomicra [Cohn, *JCI*, 1990; Iankova, *Endocrin*, 2008; Tanner, *Exp Bio Med*, 2010].

We also determined the impact of the fasting or postprandial states on the phenotypes described in the manuscript. We found that left ventricles from HCM mice in both the fasted and fed states exhibit depressed Cd36, LpL, and Vldlr transcript levels (now Figure 1A-B). In addition to the increased plasma triglyceride levels observed in the fasted 12 month-old HCM males (Figure 2A), we found increased plasma triglyceride levels in *ad libitum* fed 12 month-old HCM males (Response Figure 3A) {Removed upon Author request}. Furthermore, we supplemented the finding of hepatic PEPCK upregulation in the fed state (Figure 7E) with quantitative RT-PCR data from livers of age-matched fasted HCM male mice showing a significant increase in PEPCK mRNA levels (Response Figure 3B) {Removed upon Author request

The manuscript previously showed that plasma catecholamine levels were the same between HCM and wildtype mice (Supporting Information 3D). This agrees with clinical data showing that there are no differences in circulating catecholamines in HCM patients [Haneda, *Cath CV Diag*, 1978; Lefroy, *JACC*, 1993; Omodani, *Clin Exp Pharm Phys*, 1998]. We demonstrate that HCM does not result in elevated plasma non-esterified fatty acid levels (Supporting Information Figure 3C, Response Figure 3C) {Removed upon Author request}, which would be a consequence of increased adrenergic signaling [Galster, *JCI*, 1981]. Catecholamines result in PEPCK activation via protein kinase A-mediated phosphorylation of cAMP-responsive element binding protein (CREB) and subsequent binding of CREB to the cAMP-responsive elements in the *Pgc1* and *Pepck* promoters. The manuscript previously showed that Pgc1 transcript levels were unchanged and that CREB phosphorylation and co-immunoprecipitation with the *Pepck* promoter were substantially reduced (Figure 7C, Supporting Information Figure 13B-C). We have added data bolstering the adrenergic-independent activation of hepatic mitogen-activated protein kinase (MAPK) signaling and PEPCK expression with the finding that norepinephrine levels in the liver are unaffected by HCM (now Supporting Information Figure 13A).

In response to the second review, we have also made direct measurements of glucocorticoid levels to assuage concerns that the observed

phenotype may have been caused by sampling/euthanasia methods or a non-specific stress response due to HCM. We found that neither circulating nor hepatic cortisol and corticosterone levels were elevated in HCM mice (now Supporting Information Figure 13D-G, Response Figure 4A-B) {Removed upon Author request} . Combined with the lack of depressed insulin signaling activity and ketone levels, increased VLDL triglyceride secretion or plasma non-esterified fatty acid levels, and upregulation of the hepatic glucocorticoid receptor targets fatty acid synthase, PPARα, 11β-hydroxysteroid dehydrogenase-1, angiopoietin-related protein-4 or maltase-glucoamylase expression (Figures 3E-F, Supporting Information Figure 3C and 13H, Response Figure 4C-D) {Removed upon Author request}, the observed phenotype in the HCM liver is glucocorticoid-independent [Cole, *JLR*, 1982; Paterson, *PNAS*, 2004; Sai, *Mol Endocrin*, 2008; Staab and Maser, *J Ster Biochem Mol Bio*, 2009].

2. "The re-revised version should at least provide an analysis of hepatic PPAR alpha activity."

We shared the reviewer's concerns regarding PPARα. From our earliest assessments of a hepatic response to HCM (e.g. lipid accumulation and PEPCK activity), we hypothesized that PPARα activity might be responsible. However, it became increasingly clear that elevated PEPCK activity was the result of a PPAR-independent pathway (Supporting Information Figure 12 G-H). In fact, numerous studies fail to show any change in PEPCK expression with PPARα agonists (e.g. WY16,643 or ciprofibrate) or in PPARα null mice [Bandsma, *JBC*, 2004; Davies, *Biochem Pharm*, 2001; Kersten, *JCI*, 1999; Xu, *JBC*, 2002; Yadetie, *Physiol Genomics*, 2003]. However, we have carried out additional analysis to further address the reviewer's concerns.

An early experiment assessing PGC-1α activity and binding partners (Supporting Information Figure 12B) showed us that HCM did not result in an increase in PGC-1α/PPARα interactions (Response Figure 5A-C){Removed upon Author request}. Since an increase in PPARα activity is accompanied by an increase in PPARα levels [Pineda Torra, *Mol Endocrin*, 2002], we measured PPARα expression. We found that both PPARα transcript and protein levels were unchanged in the HCM liver (Response Figure 5D-F){Removed upon Author request}. Figures in the manuscript previously showed that the expression of numerous well-characterized PPARα targets including Mttp, Dgat1, Fasn, Pgc1, Cd36, Vldlr, Angptl4, Lpl, Mgat1 [Rakhshandehroo, *PPAR Res.*, 2007] was unchanged in the HCM liver (Figures 4F and 7C, Supporting Information Figures 13H). In response to the review, we measured the expression of an additional 16 PPARα targets in the fed liver (for a total of 25 target genes), including 10 *canonical PPARα targets,* none of which were elevated. In fact, 2 of the genes deemed *canonical PPARα targets* (long-chain acyl-CoA synthase and acyl-CoA oxidase), were significantly downregulated (Response Figure 6A) {Removed upon Author request}. We also found that 7 of these *canonical PPARα targets* were not upregulated in fasted HCM livers either (Response Figure 6B) {Removed upon Author request}. These data strongly support

the lack of a role for PPARα in the hepatic phenotype described in the manuscript.

We believe that all of the additions have reconciled the remaining reviewer's concerns and improved the manuscript in the hope that it will now be acceptable to EMBO Molecular Medicine.

New Experiments for Oct. 2013 and *Sept. 2013* submissions

1. Test the effects of anaesthesia in wildtype control mice
    a. Blood glucose (Response Fig 1A) {Removed upon Author request}
    b. Plasma non-esterified fatty acids (Response Fig 1B) {Removed upon Author request}
    c. Plasma immunoreactive insulin and homeostatic model assessment of insulin resistance (Response Fig 1C-D) {Removed upon Author request}
    d. Glucose tolerance (Response Fig 1E-F) {Removed upon Author request}

2. Test the effects of fasting
    a. Fasted vs. fed wildtype controls
        i. Plasma and VLDL triglyceride levels (Response Fig 2A) {Removed upon Author request}
        ii. Plasma and liver norepinephrine content (Response Fig 2B) {Removed upon Author request}
        iii. Feeding-induced triglyceride and catecholamine variability (Response Fig 2C) {Removed upon Author request}
        iv. Plasma non-esterified fatty acid levels (Response Fig 2D) {Removed upon Author request}
    b. Fasted and fed wildtype and HCM males
        i. Plasma triglycerides in fed (Response Fig 3A) {Removed upon Author request}
        ii. PEPCK expression in fasted mice (Response Fig 3B) {Removed upon Author request}

3. Adrenergic activity
    a. Hepatic norepinephrine content in wildtype and HCM males (Supp Fig 13A)
    b. Plasma non-esterified fatty acid levels (Response Figure 3C) {Removed upon Author request}

4. Altered glucocorticoid levels
    a. Hepatic glucocorticoid levels
        i. Regression analysis of hepatic PEPCK expression and cortisol content (Supp Fig 13D)
        ii. Hepatic cortisol content in the fasted and fed states (Supp Fig 13E)
        iii. Hepatic corticosterone content in the fasted and fed states (Response Fig 4A) {Removed upon Author request}
        iv. Regression analysis of hepatic PEPCK expression and corticosterone content (Response Fig 4B) {Removed upon Author request}
    b. Circulating glucocorticoids
        i. Plasma cortisol levels (Supp Fig 13F)
        ii. Plasma corticosterone levels (Supp Fig 13G)
    c. Markers of elevated hepatic glucocorticoid activity
        i. Plasma ketone levels (Response Fig 4C) {Removed upon Author request}
        ii. Hepatic insulin-dependent AKT phosphorylation (Response Fig 4D) {Removed upon Author request}

5. Analysis of hepatic PPARα activity
    a. PEPCK expression mediated by PPARα/PGC1α

        i. Co-immunoprecipitation of hepatic PGC1α and PPARα (Response Fig 5A-B) {Removed upon Author request}
        ii. Regression analysis of PEPCK expression versus PGC1α-bound PPARα (Response Fig 5C) {Removed upon Author request}
        iii. Regression analysis of PEPCK expression versus PPARα expression (Response Fig 5D) {Removed upon Author request}
        iv. Regression analysis of PEPCK expression versus HNF4α expression (Supp Fig 12H)
    b. PPARα Expression
        i. PPARα transcript levels (Response Fig 5E) {Removed upon Author request}
        ii. PPARα protein expression (Response Fig 5F) {Removed upon Author request
    c. PPARα target gene expression
        i. In fed livers (Response Fig 6A) {Removed upon Author request}
        ii. In fasted livers (Response Fig 6B) {Removed upon Author request}

6. *Heart*
    a. *Cardiac lipids*
        i. *Left ventricular fatty acid composition*
            1. *In the triglyceride pool (Fig2C)*
            2. *Total lipid, After vehicle or AICAR (Fig3E)*
        ii. *Left ventricular fatty acid content*
            1. *After vehicle or AICAR (Fig3D)*

    b. *Earlier time-points of ventricular metabolic changes*
        i. *CD36 expression and AMPK activity (Supp Fig5F-G)*
        ii. *Lipid content (Supp Fig5I)*
        iii. *AMPK phosphorylation (Supp 7B-C)*

    c. *Left ventricular regression analyses*
        i. *Beta Myosin mRNA vs. CD36 protein expression (Fig 3A)*
        ii. *AMPK phosphorylation vs. CD36 protein expression (Supp Fig 7A)*

7. *Liver and Hepatocytes*
    a. *Hepatic fatty acid composition*
        i. *In the isolated triglyceride, diacylglycerol and free fatty acid pools (Fig6A)*

    b. *Hepatic signaling and transcriptional changes*
        i. *ChIP for CREB-bound Pepck promoter (Supp Fig 13B)*

    c. *Liver regression analyses*
        i. *PKCa vs. p-p38 MAPK (Fig 6D)*
        ii. *PKCβII/γ/δ/θ vs. p-p38 MAPK (Supp Fig 10C-F)*

iii. Triglyceride content vs. p-p38 MAPK (Fig 6E)
iv. VLDL Oleic acid content of plasma vs. fasting blood glucose levels (Fig 7I)
v. Lipid content vs. cardiac CD36 expression (Supp Fig 9)
vi. Circulating VLDL triglyceride vs. ApoB (Supp Fig 4C-D)

d. Hepatocellular regression analysis
i. Triglyceride content vs. p-p38 MAPK (Fig 6G)
ii. VLDL Oleic acid content added to culture vs. PEPCK expression (Fig 7B)
iii. VLDL triglyceride content added to culture vs. PEPCK expression (Supp Fig 10J)
iv. Quantitative enzymatic determination of lipid content (Supp Fig 9F-G)
v. Fatty acid content vs. PGC-1 expression (Supp Fig 11A)

8. *TAC Comparison: HCM vs. pressure-overload model*
    a. *Ventricular FoxO1 expression (Supp Fig 6A,H)*
    b. *Ventricular CD36 expression (Supp Fig 6D-E)*
    c. *Ventricular lipid content (Supp Fig 6F-G)*
    d. *R-R interval by echocardiography (Supp Fig7G)*
    e. *Quantitative enzymatic determination of plasma and VLDL triglycerides (Supp Fig 15A)*

9. *3-MPA Mechanism*
   Determination of "glucotoxicity" in the HCM ventricle
    a. *Monoacylglycerol acyltransferase-2 expression (Supp Fig 16A)*
    b. *Diacylglycerol content (Supp Fig 16B)*
    c. *Advanced glycated end-product (AGE) content (Supp Fig 16C)*
    d. *Expression of genes regulated by AGE content (Supp Fig 16D, E, H, I)*
    e. *Reactive oxygen species content (Supp Fig 16F)*
    f. *Lipid peroxide content (Supp Fig 16G)*

   Effects of gluconeogenesis inhibition (via MPA)
    a. *Heart rate, determined by echocardiography (Supp Fig 17E)*
    b. *Caspase9 activity (Supp Fig 18A-B)*
    c. *Transcriptional changes of glucose-regulated genes (Supp Fig 18C)*

3rd Editorial Decision 03 December 2013

Thank you again for the submission of your revised manuscript to EMBO Molecular Medicine. We have now received the enclosed report from the Reviewer who was asked to re-assess it. As you will see the Reviewer is now supportive pending a number of final amendments to the text and presentation of data.

Please address the Reviewer's concerns and indicate the changes applied in your cover letter. Please note that I will not be asking you to perform any additional experiments and we are prepared to make an editorial decision on your next, final version.

I would like to take the opportunity, at this stage, to ask the following:

We would need a short list (up to 5) of bullet points that summarize the key NEW findings. The bullet points should be designed to be complementary to the abstract and will be used online in our new platform (coming January 2014).

Finally, we are now encouraging the publication of source data, particularly for electrophoretic gels and blots, with the aim of making primary data more accessible and transparent to the reader. Would you be willing to provide a PDF file per figure that contains the original, uncropped and unprocessed scans of all or at least the key gels used in the manuscript? The PDF files should be labeled with the appropriate figure/panel number, and should have molecular weight markers; further annotation may be useful but is not essential. The PDF files will be published online with the article as supplementary "Source Data" files. If you have any questions regarding this just contact me.

Please submit your revised manuscript within two weeks. I look forward to reading the next, final version of your manuscript as soon as possible.

***** Reviewer's comments *****

Referee #2 (Remarks):

Reiterating previous reviews, the authors present a compelling and important study describing the culmination of metabolic changes in a mouse model of familial hypertrophic cardiomyopathy, clearly demonstrating an association between cardiac and hepatic lipid crosstalk. The manuscript still falls short of going the distance in terms of solid demonstration of the mechanism proposed. However, with minor modifications to the text and ways in which specific points of data are plotted, acceptance of this manuscript will be enthusiastically supported.

Suggestions and critiques to enhance the validity of the authors' conclusions are outlined as follows:

1. Rapid decapitation is standard protocol for assessment of the sympathoadrenal axis and is well documented throughout the literature. Additionally well-documented are the effects of the use of anesthesia on corticosterone and catecholamine measures. In order to observe differences, it is routine practice to sample animals at a time of day during which basal levels are low, ie, at the start of the light cycle in mice. The authors' own data hint at a trend toward increased plasma corticosterone levels in the HCM model (response Fig. 4A). However, given that the authors' local animal welfare regulatory committee will not approve using the technique and that it would be unreasonable to request repeating the experiments at the appropriate time of day, the gene expression data would have to suffice to rule out the possibility of GR or PPAR involvement. Therefore, the newly generated PPAR pathway assessment presented in response Fig.6 should be included. Comparison of fed vs fasted states in the wildtype animals would serve as positive controls for hepatic gene expression changes measured in response Fig6 and in sup Fig13H. The lack of observable effects in fed vs fasted wildtype animals limits the utility of these data for ruling out effects of GR or PPARa pathways. Perhaps it is simply a matter of changing the way in which

the data are normalized and plotted, ie, for each gene plot data normalized to the wildtype ad lib fed group.

2. Corticosterone is the major and primary glucocorticoid in mice, the level of which is typically around 100ng/mL in plasma from mice harvested by decapitation at the same time of day used by the authors. Hepatic and plasma corticosterone measures should be included. In sup Fig13G, the y-axis is labeled as pg/mL. Is this correct?

3. In its current form, the proposed mechanism (PGC1a+HNF4a on the PEPCK promoter to the exclusion of GR or PPARa) is highly correlative. Indeed there are conflicting reports of a role for direct PPARa action on the PEPCK promoter, though some studies have shown induction by synthetic agonists and multiple PPREs on the PEPCK promoter have been experimentally validated. PPARa-responsiveness is dependent upon the time of day at which tissues are harvested, thus shedding light on the apparent discrepancies in the literature. Given that PPARa and HNF4a share the same DR1-type response element, and that PPARa synergizes with GR, ChIP of PGC1a alone is not sufficient to warrant the conclusion that HNF4a occupies the PEPCK promoter to the exclusion of PPARa or GR. Demonstrating direct binding of HNF4a on the PEPCK promoter in livers of the HCM model would be the most direct form of evidence.

4. In Fig 2c, is the decreased oleic acid significant? In Fig6A is the TG, DAG, FA significant?

5. Supp Fig12F contrasts response Fig6B... why the discrepancy in CPT1a?

6. Time of tissue harvest should be included in the methods. Currently, only time of glucose sampling is given.

3nd Revision - authors' response 20 December 2013

In our latest revision and response, we have attempted to address every concern expressed by Referee #2.

Referee #2 (Remarks): Reiterating previous reviews, the authors present a compelling and important study describing the culmination of metabolic changes in a mouse model of familial hypertrophic cardiomyopathy, clearly demonstrating an association between cardiac and hepatic lipid crosstalk. The manuscript still falls short of going the distance in terms of solid demonstration of the mechanism proposed. However, with minor modifications to the text and ways in which specific points of data are plotted, acceptance of this manuscript will be enthusiastically supported. Suggestions and critiques to enhance the validity of the authors' conclusions are outlined as follows:

1. Rapid decapitation is standard protocol for assessment of the sympathoadrenal axis and is well documented throughout the literature. Additionally well-documented are the effects of the use of anaesthesia on corticosterone and catecholamine measures. In order to observe differences, it is routine practice to sample animals at a time of day during which basal levels are low, ie, at the start of the light cycle in mice. The authors' own data hint at a trend toward increased plasma corticosterone levels in the HCM model (response Fig. 4A). However, given that the authors' local animal welfare regulatory committee will not approve using the technique and that it would be unreasonable to request repeating the experiments at the appropriate time of day, the gene expression data would have to suffice to rule out the possibility of GR or PPAR involvement. Therefore, the newly generated PPAR pathway assessment presented in response Fig.6 should be included. Comparison of fed vs fasted states in the wild type animals would serve as positive controls for hepatic gene expression changes measured in response Fig6 and in sup Fig13H. The lack of observable effects in fed vs fasted wild type animals limits the utility of these data for ruling out effects of GR or PPARa pathways. Perhaps it is simply a matter of changing the way in which the data are normalized and plotted, i.e., for each gene plot data normalized to the wild type ad lib fed group.

Response: We appreciate that the Reviewer recognizes the limitations of our local animal welfare

committee.

Murine plasma corticosterone levels are lowest and consistent throughout the light cycle, and peak (with an approximate 3-fold increase) at the start of the dark cycle [Oster, *Cell Metab*, 2006]. Therefore, sampling at 16:00 hours, as indicated in the manuscript, ensures measurement during a basal, stable and consistent period of circulating glucocorticoid levels. The differences between wild type and HCM hepatic corticosterone did not approach statistical significance in either the fed or fasted states, regardless of the statistical test employed (i.e. ANOVA or student's t-test).

As requested by Reviewer 2, we have added some of the data found in Response Figure 6 {Removed upon Author request} with a "comparison of fed vs fasted states in the wild type animals" to the manuscript (Supp Figure 14A-F). Also, please see response to comment #6

2. *Corticosterone is the major and primary glucocorticoid in mice, the level of which is typically around 100ng/mL in plasma from mice harvested by decapitation at the same time of day used by the authors. Hepatic and plasma corticosterone measures should be included. In sup Fig13G, the y-axis is labelled as pg/mL. Is this correct?*

Response: We agree fully with the reviewer. Unfortunately, our y-axis for Supp Fig 13G was inappropriately labelled, and our results now fall within that range. We apologize and have corrected this error.

Although corticosterone, as our data shows, is the prevailing circulating glucocorticoid (~3x greater concentration than cortisol), cortisol appears to be the primary glucocorticoid in the liver (~15-40x greater concentration than corticosterone). In fact, corticosterone levels were not detected in all the liver samples tested. Therefore, in order to respond directly to Reviewer 2's previous concerns regarding a glucocorticoid surge and limit the manuscript's expansion, we included data regarding both circulating glucocorticoids, but only hepatic cortisol levels (all of which were well within the range of the assay's standard curve).

3. In its current form, the proposed mechanism (PGC1a+HNF4a on the PEPCK promoter to the exclusion of GR or PPARa) is highly correlative. Indeed there are conflicting reports of a role for direct PPARa action on the PEPCK promoter, though some studies have shown induction by synthetic agonists and multiple PPREs on the PEPCK promoter have been experimentally validated. PPARa-responsiveness is dependent upon the time of day at which tissues are harvested, thus shedding light on the apparent discrepancies in the literature. Given that PPARa and HNF4a share the same DR1-type response element, and that PPARa synergizes with GR, ChIP of PGC1a alone is not sufficient to warrant the conclusion that HNF4a occupies the PEPCK promoter to the exclusion of PPARa or GR. Demonstrating direct binding of HNF4a on the PEPCK promoter in livers of the HCM model would be the most direct form of evidence.

Response: While we agree with Reviewer 2 that both PPARα and HNF4 bind DR1-type response elements, which have a degenerate sequence, the available literature demonstrates that PPAR heterodimers and HNF4 homodimers bind separate DR-1 motifs in the *Pepck* promoter. Any binding to the *Pepck* promoter by PPAR isoforms is thought to occur at a distal AF1 site (almost 500bp upstream from the 5' end of the ChIP amplicon), and is isolated to adipocytes [Devine, JBC, 1999]. In fact, mutating the proximal DR1-containing AF1 element of the *Pepck* promoter (amplified in our ChIP experiment) retains the ability to be activated by a PPARa agonist in adipocytes [Franckhauser-Vogel, *Mol Cell Endocrinology*, 1997]. Interestingly, the second half of the direct repeat PPAR response element in the proximal AF1 domain of the *Pepck* promoter is present in humans, but absent in rodents [O'Brien, *BBA*, 1995].

The AF1 segment of the murine *Pepck* promoter that was amplified contained a single DR1 motif, loosely conforming to a direct repeat of the hexameric half-site RG(G/T)TCA sequence, with a 1 nucleotide spacer; 5'-TGACCTTTGGCC**G**^{1}TG**G**^{2}G-3' (DR1 in grey) [Nakshatri and Bhat-Nakshatri, *Nuc Acids Res*, 1998]. The 5' DR1 arm (TGACCT) represents a fully functional PPAR recognition sequence, originally found in the canonical PPARα targets, acyl CoA oxidase and fatty acid binding protein [Green and Wahli, *Mol Cell Endocrinology*, 1994; Issemann, *Biochem Soc Trans*, 1992]. However, the DR1 3' arm and downstream flanking sequences in the AF1 domain (pulled down and amplified by ChIP in our manuscript) are not found in any PPAR binding sites [Nakshatri and Bhat-Nakshatri, *Nuc Acids Res*, 1998]. This is likely due to the presence of the

guanosines (bolded and numbered above) in the last and third positions of the 3' DR1 arm and downstream flanking sequences, respectively. The presence of guanosines in these positions results in a loss of *in vitro* PPAR heterodimer interaction with the DR1 motif and transcriptional activity [Osada, *Genes to Cells*, 1997]. It is difficult to entertain a direct role for PPARα in regulating hepatic PEPCK activity when so many studies fail to show any change in PEPCK expression with PPARα agonists (e.g. WY16,643 or ciprofibrate) or in PPARα null mice [Bandsma, *JBC*, 2004; Davies, *Biochem Pharm*, 2001; Kersten, *JCI*, 1999; Xu, *JBC*, 2002; Yadetie, *Physiol Genomics*, 2003].

Although some groups have reported that HNF4 occupancy of the *Pepck* promoter does not change with hormonal stimuli [Duong, JBC, 2002], we have ChIP data indicating increased pull-down of the *Pepck* AF1 domain with HNF4 (Response Figure 1A) {Removed upon Author request}. This may simply be the product of increased HNF4 expression in the HCM liver. Therefore, we relied on the lack of direct DNA binding activity of PGC-1α and the requirement of an interaction with DNA-bound transcription factors to impact gene expression [Rhee, *PNAS*, 2003] to implicate PGC-1α in the HNF4-mediated association with the AF1 domain of *Pepck* and activation of PEPCK expression.

4. In Fig 2c, is the decreased oleic acid significant?

5. In Fig6A is the TG, DAG, FA significant?

Response: The neutral lipid fractions separated by thin-layer chromatography were of low enough abundance to warrant pooling in order to generate sufficient signal intensity by gas chromatography to confidently identify and quantify individual fatty acid species. Therefore, statistical analyses were not possible. However, the fraction-specific changes in oleic acid conform to changes in the total lipid pool for those respective tissues, where statistical analyses were possible (Figures 3E, 4E).

6. Supp Fig12F contrasts response Fig6B... why the discrepancy in CPT1a?

Response: Supp Fig 12F represents the fed state, while Response Fig 6B represents the fasted state. Cpt1α is a canonical PPARαtarget in the liver. Fasting, due to the increase in circulating free fatty acids, is a well-characterized stimulus of PPARα activity in the liver [Leone, *PNAS*, 1999]. This is also apparent in our studies when we combine hepatic CPT1α expression data for fed and fasted mice (Supp Figure 14A). However, in striking contrast to PEPCK up regulation in both states, any increase in Cpt1α (and other canonical PPARα targets) is not maintained as PPARα is similarly activated with fasting in both wild type and HCM livers (Supp Figure 14A-E, Response Figure 1B) {Removed upon Author request}. Interestingly, fasting failed to increase the hepatic expression of Pdk4, another canonical PPARα target, in HCM males (Supp Figure 14F). This expression profile of PPARα targets suggests that PPARα activity is not elevated in the HCM liver in either the *ad libitum* fed or fasted states and that the described phenotype (i.e. PEPCK up regulation) is independent of PPARα activity.

7. Time of tissue harvest should be included in the methods. Currently, only time of glucose sampling is given.

Response: We have amended the manuscript to include the time of sacrifice.

We thank Referee #2 for his/her persistence, and believe their insight has helped to improve this manuscript. We believe that the above responses have reconciled the remaining reviewer's concerns in the hope that it will now be acceptable to EMBO Molecular Medicine.
