# Supplementary material for: Metabolic crosstalk between the heart and liver impacts familial hypertrophic cardiomyopathy
Source: EMBO Mol Med. 2014 Feb 24;6(4):482–95. doi: 10.1002/emmm.201302852 (PMC3992075; doi:10.1002/emmm.201302852)
Supplement: Supplementary file 2 [file emmm0006-0482-sd2.pdf]

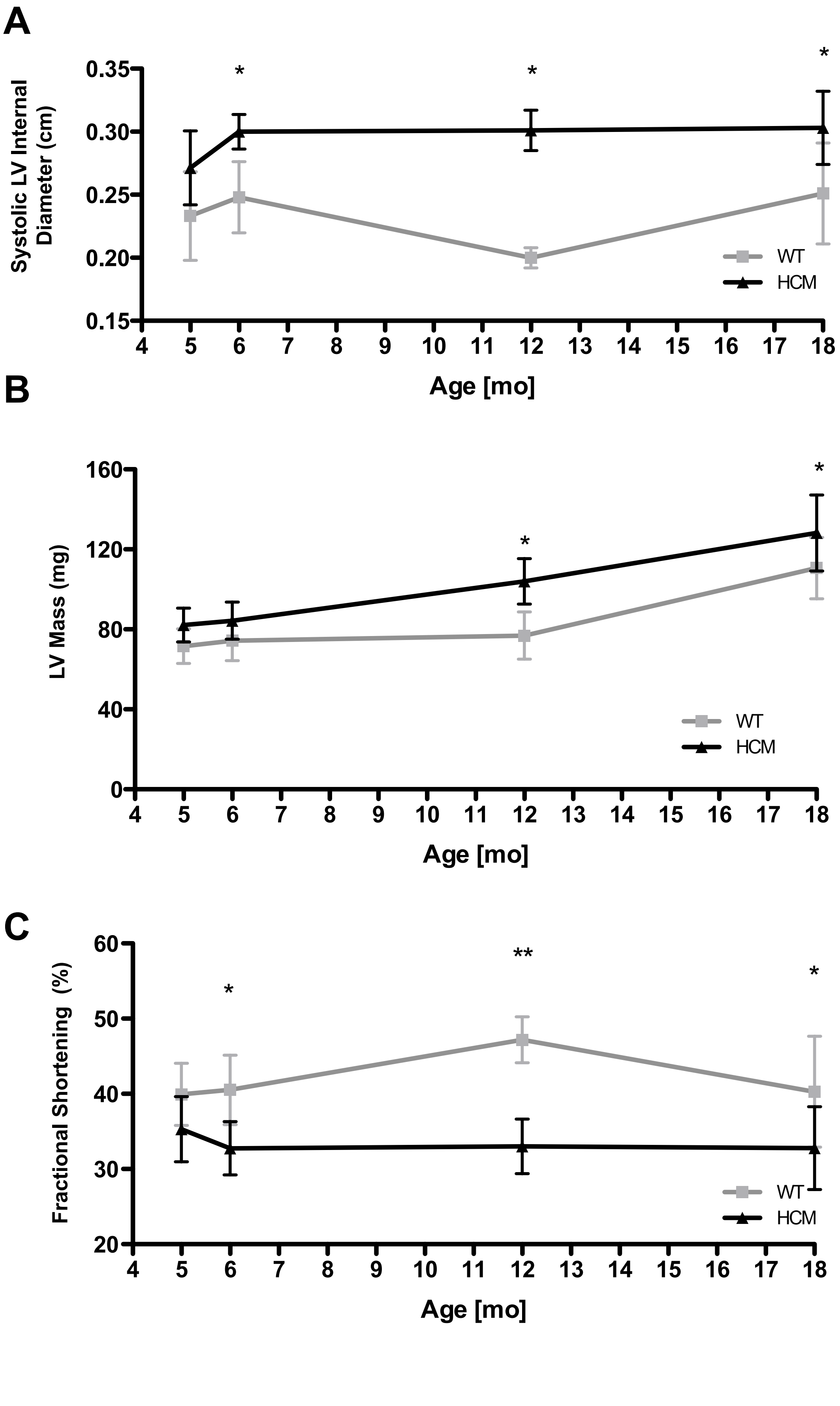

Supplemental Figure 1: Age-dependent progression of heart failure in the male HCM mouse.
(A-C) Echocardiographic timeline of cardiac morphology and function (A, left ventricular internal diameter in systole; B, mass; C, fractional shortening) in WT and HCM males. Mean±SD; *t*-test; n = 4-10. *Significantly different ($P \leq 0.05$); ** ($P \leq 0.01$) from WT control.
