# Supplementary material for: Metabolic crosstalk between the heart and liver impacts familial hypertrophic cardiomyopathy
Source: EMBO Mol Med. 2014 Feb 24;6(4):482–95. doi: 10.1002/emmm.201302852 (PMC3992075; doi:10.1002/emmm.201302852)
Supplement: Supplementary file 3 [file emmm0006-0482-sd3.pdf]

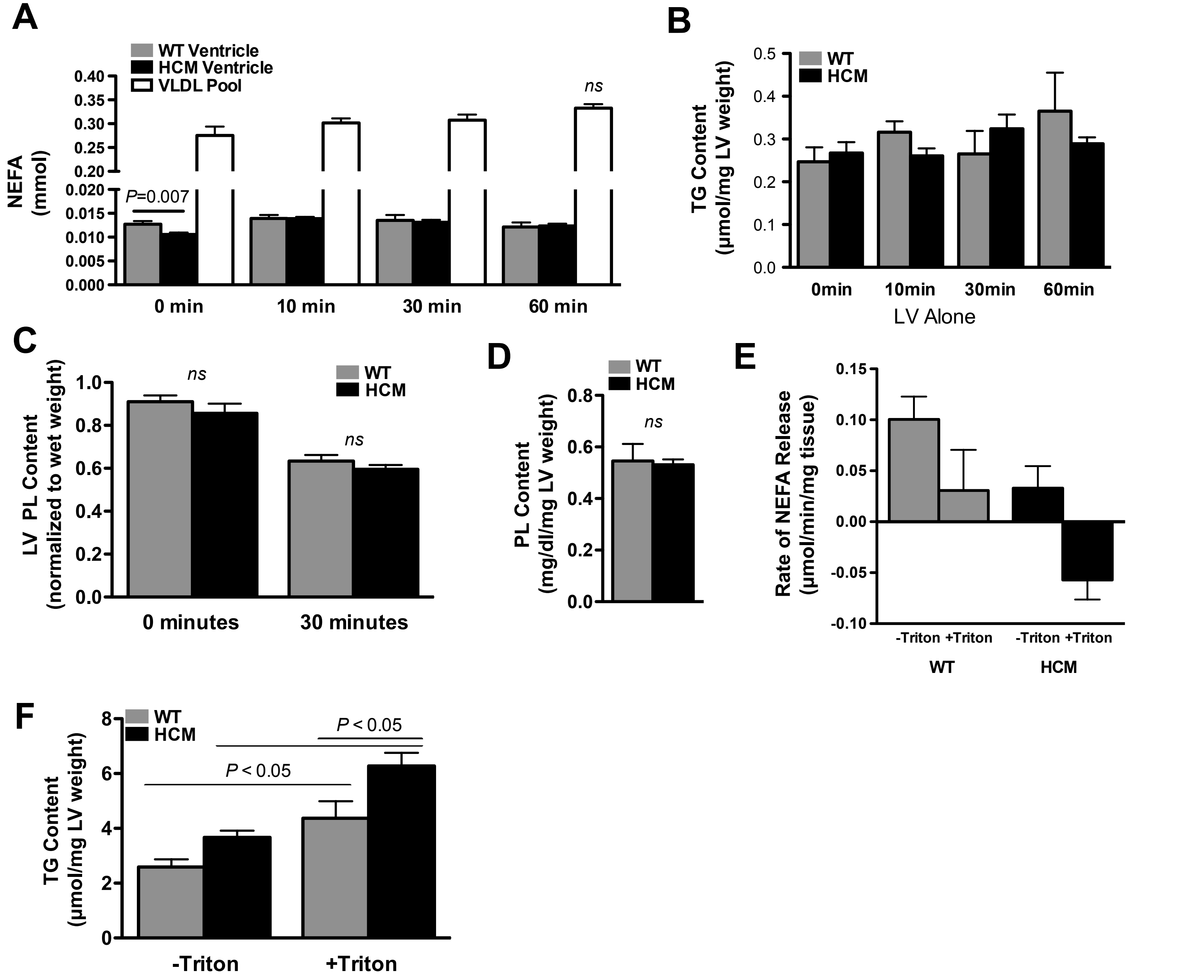

Supplemental Figure 2: VLDL TG hydrolysis. (A) Enzymatic determination of non-esterified fatty acid (NEFA) content in ventricular lysates and VLDL pools incubated separately for 0-60 minutes. Left ventricular NEFA content normalized to wet tissue weight. The differences between 0-60 minute NEFA concentrations are not significant (*ns*). Mean±SEM; ANOVA; $n = 2$-6. (B) Enzymatic determination of triglyceride (TG) content in ventricular lysates incubated in the absence of VLDL for 0-60 minutes. Mean±SEM; ANOVA; $n = 4$-5. (C) Enzymatic determination of phospholipid (PL) content in ventricular lysates incubated in the absence of VLDL for 0 or 30 minutes. Mean±SEM; *t*-test; $n = 4$-5.(D) Enzymatic determination of phospholipid (PL) content in ventricular lysates incubated with VLDL for 30 minutes. (E) Rate of non-esterified fatty acid (NEFA) release from VLDL when incubated with ventricular lysates for 30 minutes in the presence or absence of Triton WR 1339. Mean±SD; ANOVA; $n = 5$. $P = 0.0061$. (F) Enzymatic determination of VLDL TG content when incubated with ventricular lysates for 60 minutes in the presence or absence of Triton WR 1339. Normalized to ventricular TG content. Mean±SEM; ANOVA; $n = 6$.
