# Supplementary material for: Metabolic crosstalk between the heart and liver impacts familial hypertrophic cardiomyopathy
Source: EMBO Mol Med. 2014 Feb 24;6(4):482–95. doi: 10.1002/emmm.201302852 (PMC3992075; doi:10.1002/emmm.201302852)
Supplement: Supplementary file 4 [file emmm0006-0482-sd4.pdf]

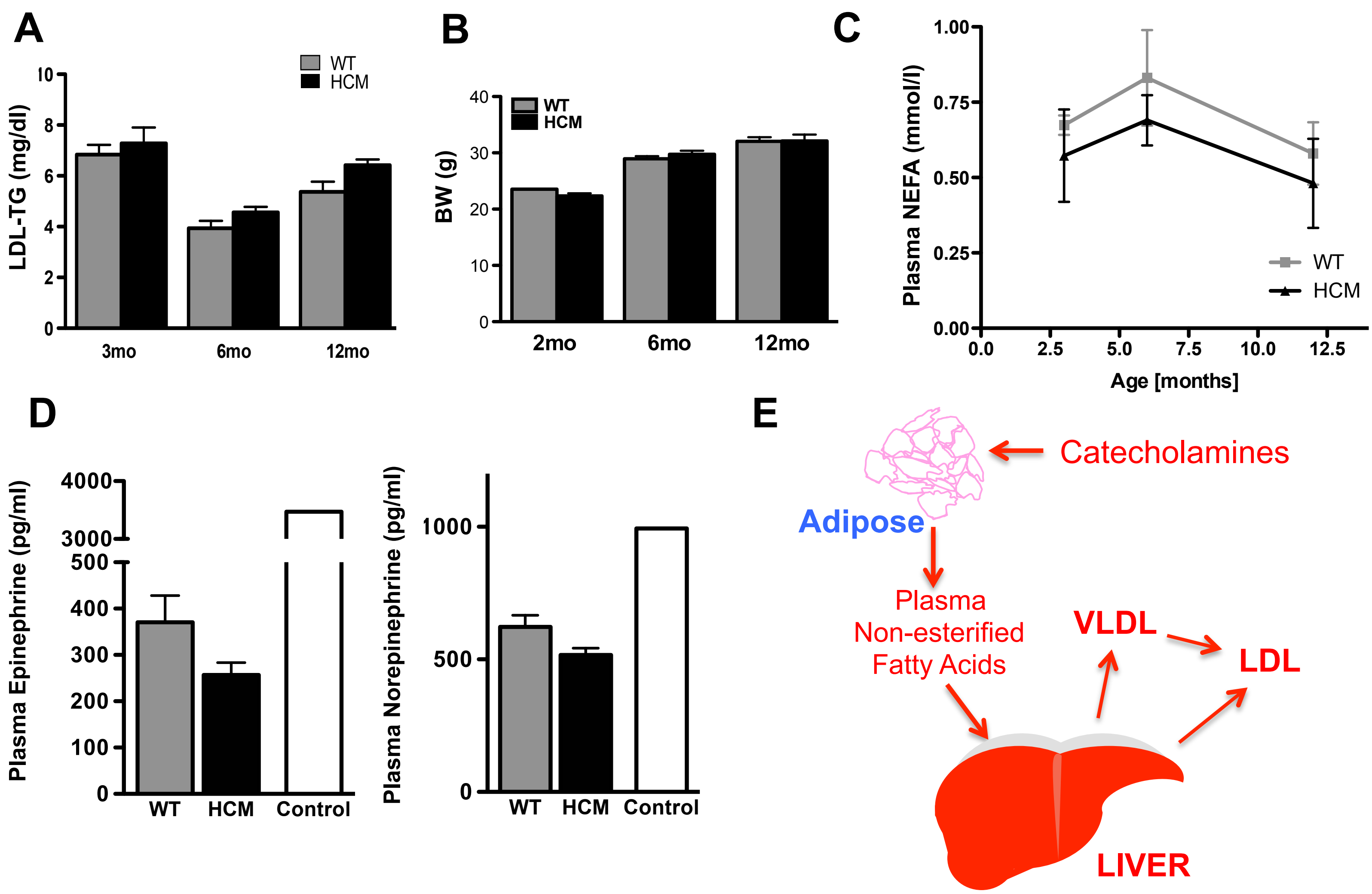

Supplemental Figure 3: Hyperadrenergic stress is absent in the male HCM mouse. (A) Timeline of fasting plasma LDL-TG in males. Mean±SEM; *t*-test; *n* = 4-9. (B) Timeline of male body weight. Mean±SEM; *t*-test; *n* = 6-17. (C) Enzymatic measurement of fasting plasma non-esterified fatty acid (NEFA) levels. Mean±SD; *t*-test; *n*=4-9. (D) Fasted plasma catecholamines in 12 month-old males. Control was provided by the assay manufacturer. Mean±SEM; *t*-test; *n* = 4. (E) Diagrammatic description of catecholamine-induced lipolysis, plasma fatty acid surge, hepatic clearance and lipoprotein synthesis/secretion.
