# Supplementary material for: Metabolic crosstalk between the heart and liver impacts familial hypertrophic cardiomyopathy
Source: EMBO Mol Med. 2014 Feb 24;6(4):482–95. doi: 10.1002/emmm.201302852 (PMC3992075; doi:10.1002/emmm.201302852)
Supplement: Supplementary file 5 [file emmm0006-0482-sd5.pdf]

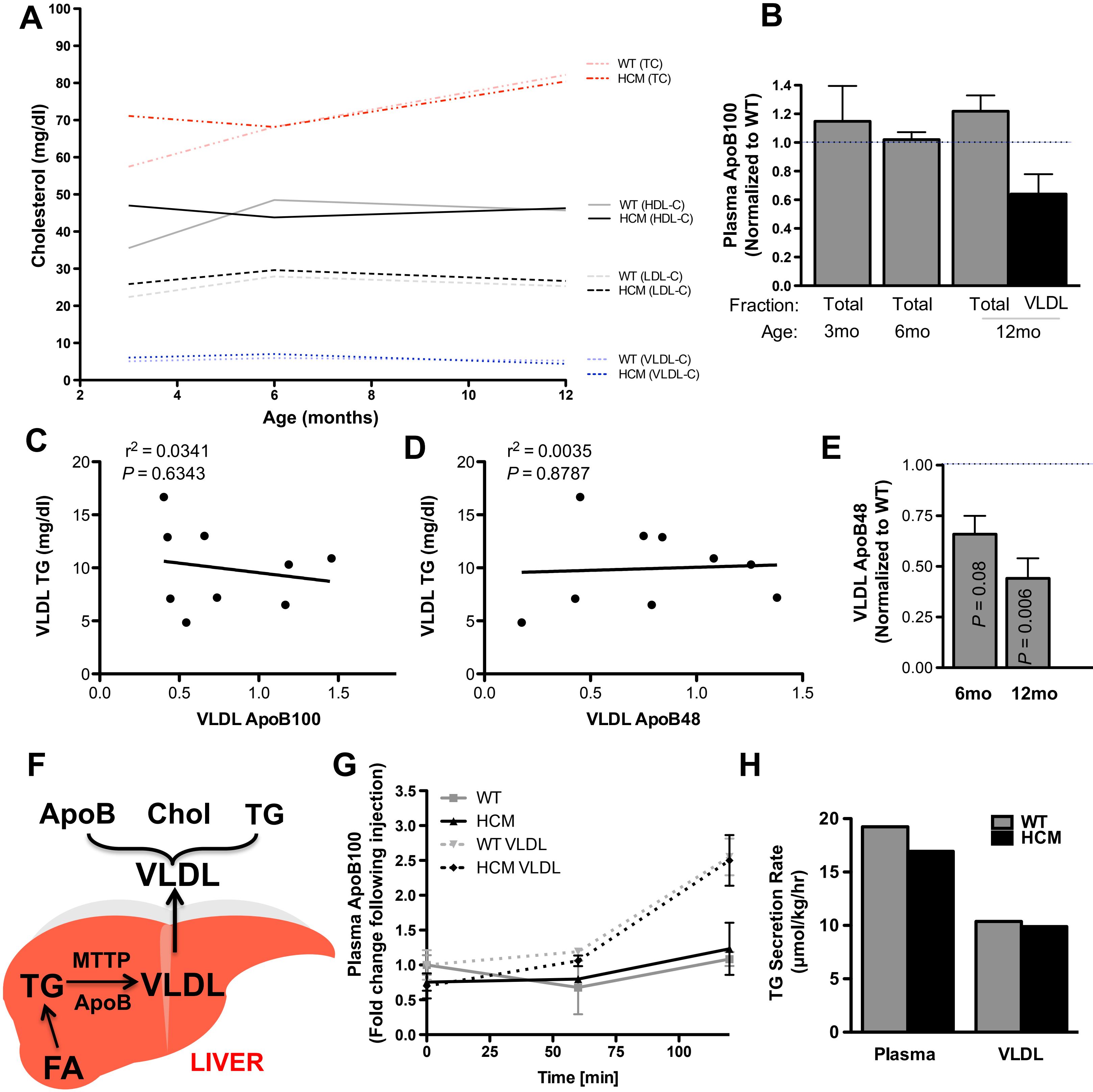

Supplemental Figure 4: VLDL secretion in the male HCM mouse is unaltered. (A) Timeline of plasma and lipoprotein cholesterol levels; total (TC), HDL, LDL, and VLDL in WT and HCM male mice. Mean; *t*-test; *n* = 4-7. (B) Timeline of plasma and VLDL ApoB100 protein levels; determined by immunoblot. Values represent fold change in male HCM mice relative to wildtype controls. Mean±SEM; *t*-test; *n* = 2-6. (C-D) Regression analyses of triglyceride (TG) and apolipoproteinB (ApoB)-100 or ApoB-48 content in the VLDL fraction at 12 months. (E) 6 and 12 month-old VLDL ApoB48 protein levels; determined by immunoblot. Values represent fold change in male HCM mice relative to wildtype controls. Mean±SEM; *t*-test; *n* = 4-6. (F) Diagram of hepatic VLDL synthesis/ secretion, resulting in proportional increases in TG, ApolipoproteinB and cholesterol. (G) Secretion of ApoB100 protein into plasma and VLDL fractions following inhibition of peripheral lipase activity with Triton WR1339. Mean ±SD; *t*-test; *n* = 3. (H) Secretion rates of plasma and VLDL TG between 60-200 minutes after Triton WR1339 injection. *n* = 5.
