# Supplementary material for: Metabolic crosstalk between the heart and liver impacts familial hypertrophic cardiomyopathy
Source: EMBO Mol Med. 2014 Feb 24;6(4):482–95. doi: 10.1002/emmm.201302852 (PMC3992075; doi:10.1002/emmm.201302852)
Supplement: Supplementary file 6 [file emmm0006-0482-sd6.pdf]

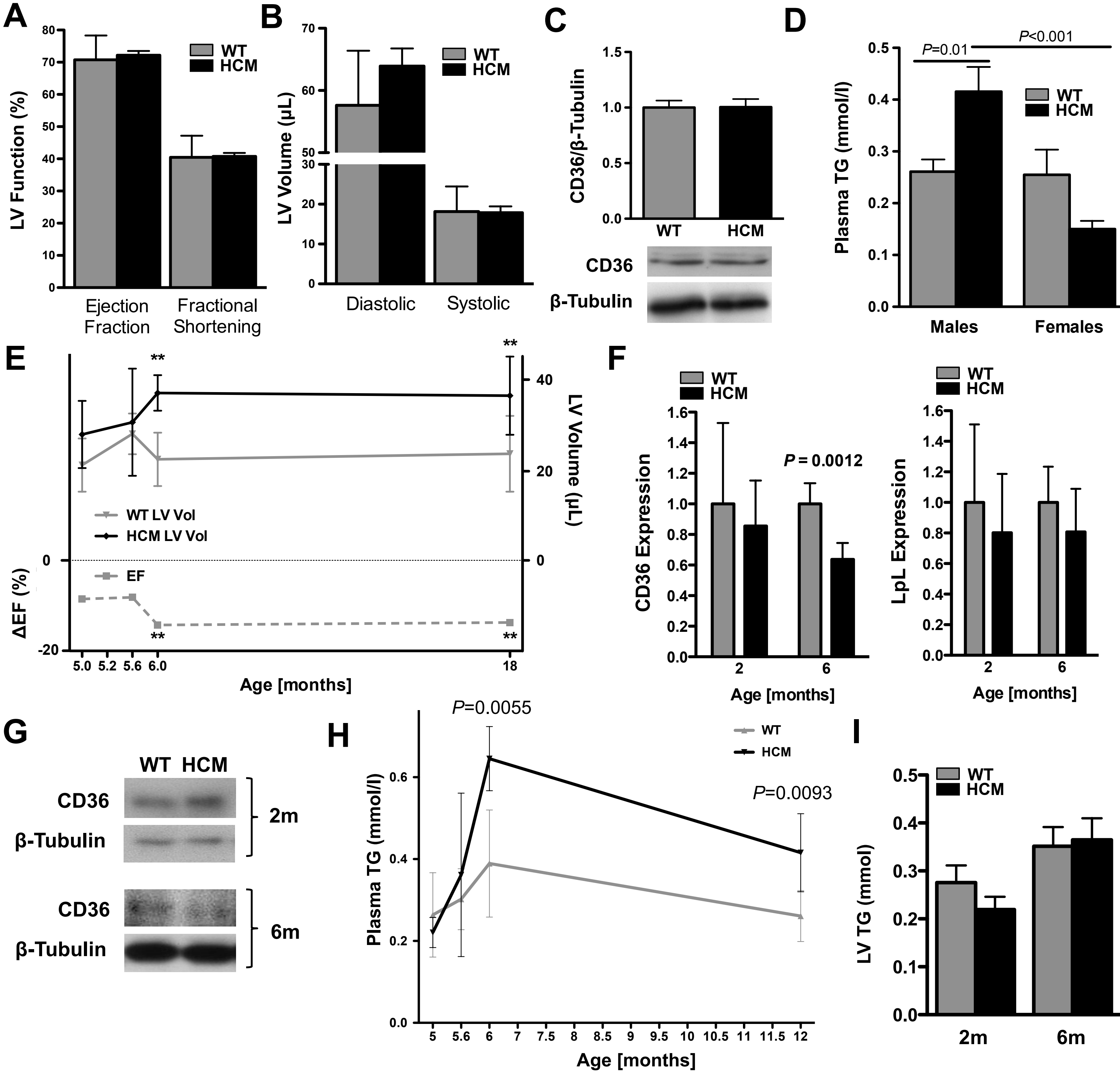

Supplemental Figure 5: The loss of ventricular CD36 expression and function associated with HCM is specific to males. (A-B) Echocardiographic determination of (A) ejection fraction and fractional shortening, and (B) chamber volume in 12 month old WT/HCM females. Mean±SEM; *t*-test; *n* = 3-4. (C) Western blot analysis of cardiac CD36 (normalized to β-tubulin) in 12 month old WT/HCM females. Mean±SEM; *t*-test; *n* = 3. (D) Fasting plasma TG levels (determined by colorimetric assay) in 12 month old male and female mice. Mean±SEM; *ANOVA*; *n*=3-7. (E) Echocardiographic demonstration of the onset of aberrant cardiac architecture and function in HCM males at 6 months. Left ventricular ejection fraction (EF, grey dashed line) in HCM males; presented as percent change when normalized to WT. Increased LV volume (solid lines); presented as mean±SD. *n*=4-8. (F) Left ventricular CD36 and lipoprotein lipase (LpL) transcript levels (determined by qPCR) in males at 2 and 6 months of age. Mean±SEM;*t-test; n*=5-8. (G) Representative western blots of left ventricular CD36 protein expression (and β-tubulin loading control) in 2 or 6 month old males. *n*=4-6. (H) Fasting plasma TG levels (determined by colorimetric assay) in 5-12 month old male mice. Mean±SEM; *t-test*; *n*=3-7. (I) Left ventricular TG content (determined by colorimetric assay and normalized to protein) in 2 and 6 month old male mice. Mean ±SEM; *t-test*; *n*=4-6. **Significantly different (P < 0.01) from wildtype control.
