# Supplementary material for: Metabolic crosstalk between the heart and liver impacts familial hypertrophic cardiomyopathy
Source: EMBO Mol Med. 2014 Feb 24;6(4):482–95. doi: 10.1002/emmm.201302852 (PMC3992075; doi:10.1002/emmm.201302852)
Supplement: Supplementary file 7 [file emmm0006-0482-sd7.pdf]

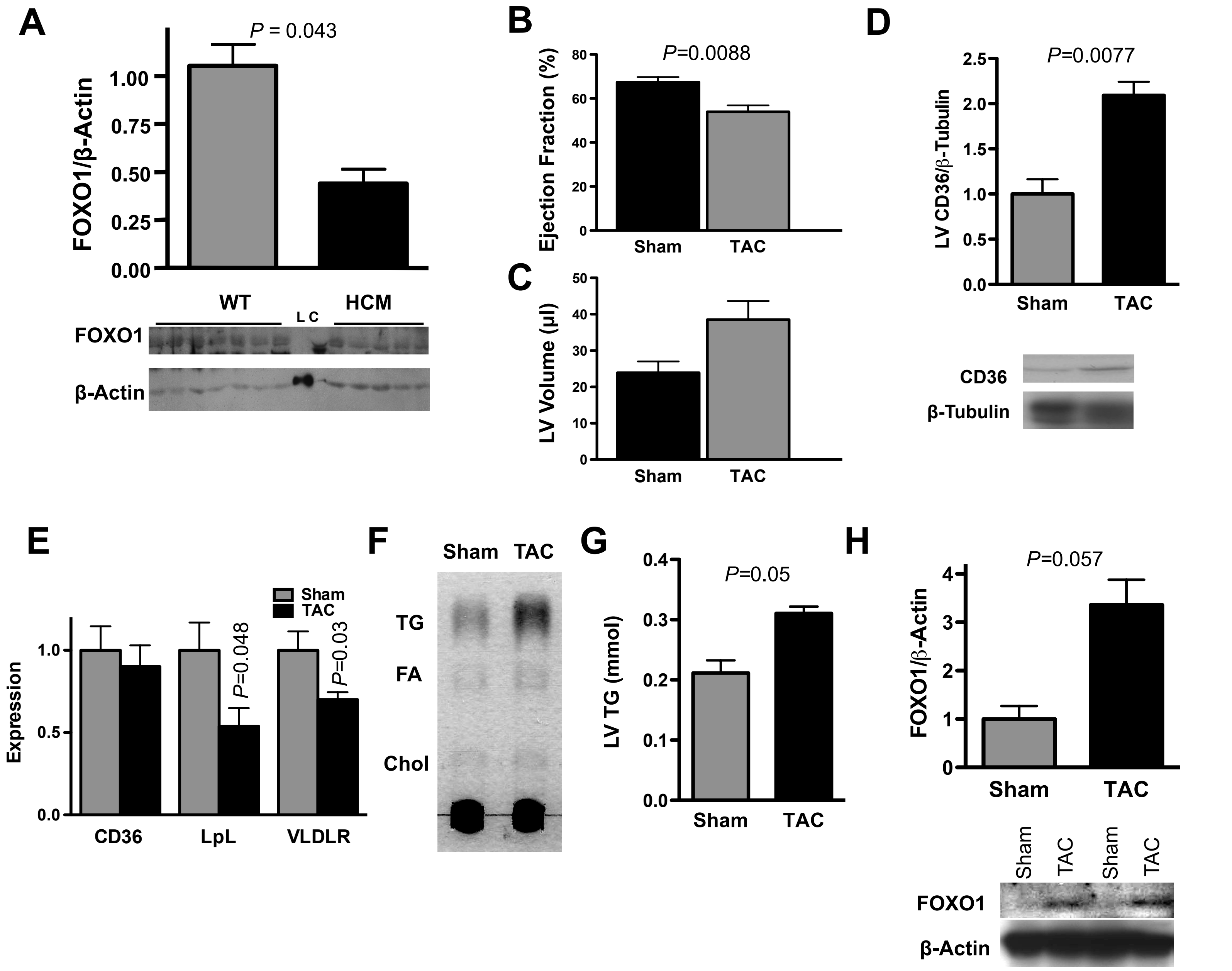

Supplemental Figure 6: Increased cardiac CD36 expression and lipid content associated with pressure-overload. (A) Western blot analysis of ventricular FOXO1(normalized to β-actin) content in 12 month WT/HCM males. Ladder, L; Control, C lanes. Mean±SEM; *t*-test; *n* = 5-7. (B-C) Echocardiographic determination of ejection fraction and systolic chamber volume in 6 month old WT males 8-10 weeks after sham surgery or aortic constriction (TAC). Mean±SEM; *t*-test; *n* = 5-9. (D) Western blot analysis of ventricular Cd36 (normalized to β-tubulin) content in WT males 8-10 weeks after sham surgery or aortic constriction (TAC). Mean±SEM; *t*-test; *n* = 3. (E) qPCR analysis of ventricular CD36, lipoprotein lipase (LpL), and VLDL receptor (VLDLR) in the TAC or sham operated mice. Mean±SEM; *t*-test; *n* = 3. (F) Thin-layer chromatography of ventricular lipid extracts from sham and TAC mice. (G) Colorimetric determination of ventricular TG content (normalized to protein) in TAC and sham operated mice. Mean±SEM; *t*-test; *n* = 3. (H) Western blot analysis of ventricular FOXO1(normalized to β-actin) content in WT males 8-10 weeks after sham surgery or aortic constriction (TAC). Mean±SEM; *t*-test; *n* = 3.
