# Supplementary material for: Metabolic crosstalk between the heart and liver impacts familial hypertrophic cardiomyopathy
Source: EMBO Mol Med. 2014 Feb 24;6(4):482–95. doi: 10.1002/emmm.201302852 (PMC3992075; doi:10.1002/emmm.201302852)
Supplement: Supplementary file 8 [file emmm0006-0482-sd8.pdf]

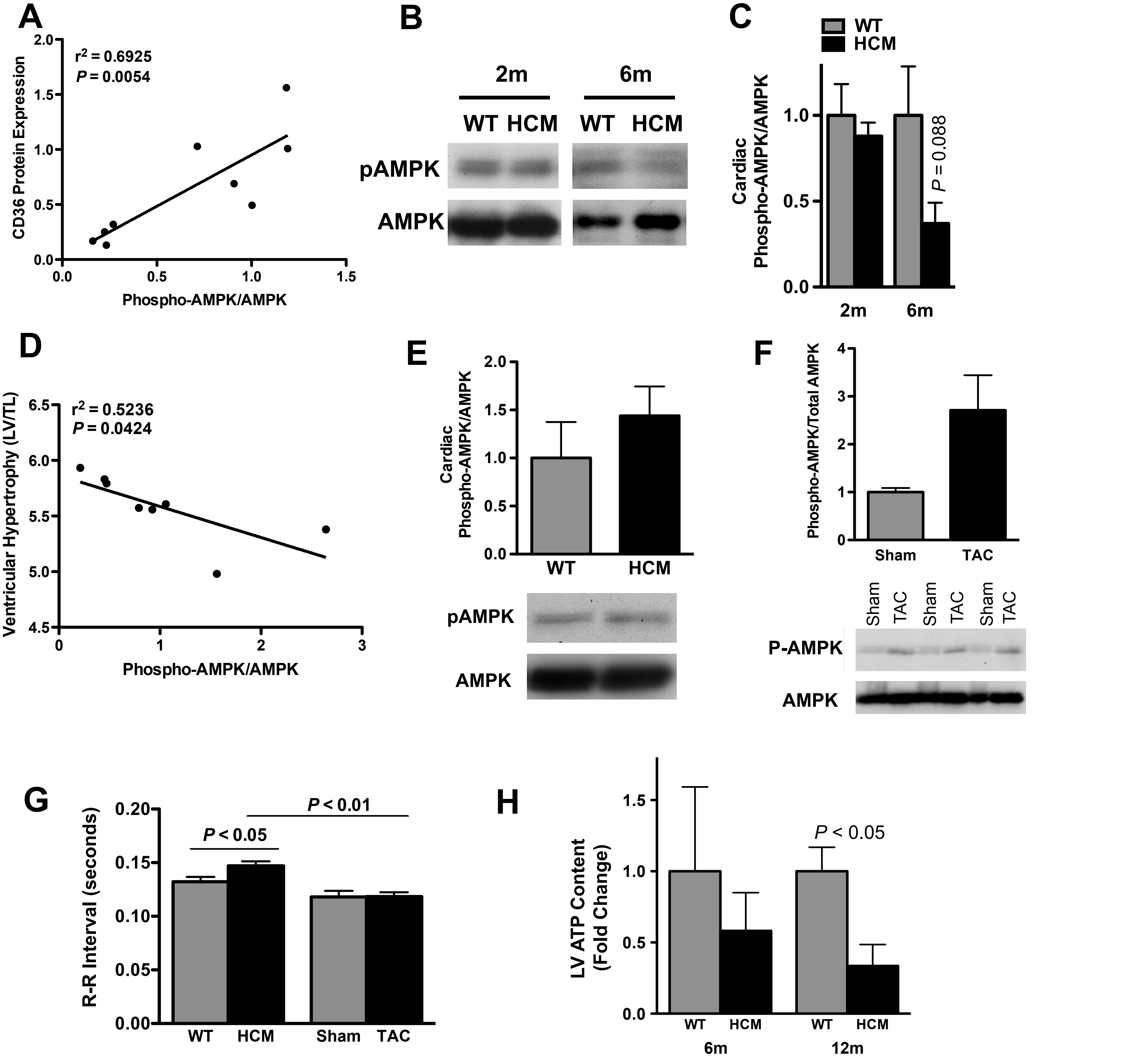

Supplemental Figure 7: Progressive loss of ventricular AMPK activity and CD36 expression in HCM males. (A) Regression analysis of ventricular CD36 protein (normalized to β-actin) content and phosphorylated AMPK (normalized to total AMPK) in 12 month-old male WT and HCM mice. (B-C) Western blot analysis of ventricular phosphorylated AMPK (normalized to total AMPK) in 2 and 6 month-old male WT and HCM mice. Mean±SEM; *t*-test; *n* = 4. (D) Regression analysis of phospho-AMPK (normalized to total AMPK) and ventricular hypertrophy (mg/mm tibia length) in 6 month-old males. (E) Western blot analysis of ventricular phosphorylated AMPK (normalized to total AMPK) in 12 month-old female WT and HCM mice. Mean±SEM; *t-test*; *n* = 3. (F) Western blot analysis of ventricular phosphorylated AMPK (normalized to total AMPK) in TAC or sham operated mice. Mean±SEM; *t-test*; *n* = 3. (G) R-R Interval of 6 month old WT/HCM males and 6 month old males 8 weeks after arotic constriction or sham surgery. Determined by echocardiography. Mean±SEM; *ANOVA* ; *n* = 5-8. (H) Ventricular ATP content in 6 and 12 month old male mice. Mean±SEM; *t-test*; *n* = 3-8.
