# Supplementary material for: Metabolic crosstalk between the heart and liver impacts familial hypertrophic cardiomyopathy
Source: EMBO Mol Med. 2014 Feb 24;6(4):482–95. doi: 10.1002/emmm.201302852 (PMC3992075; doi:10.1002/emmm.201302852)
Supplement: Supplementary file 9 [file emmm0006-0482-sd9.pdf]

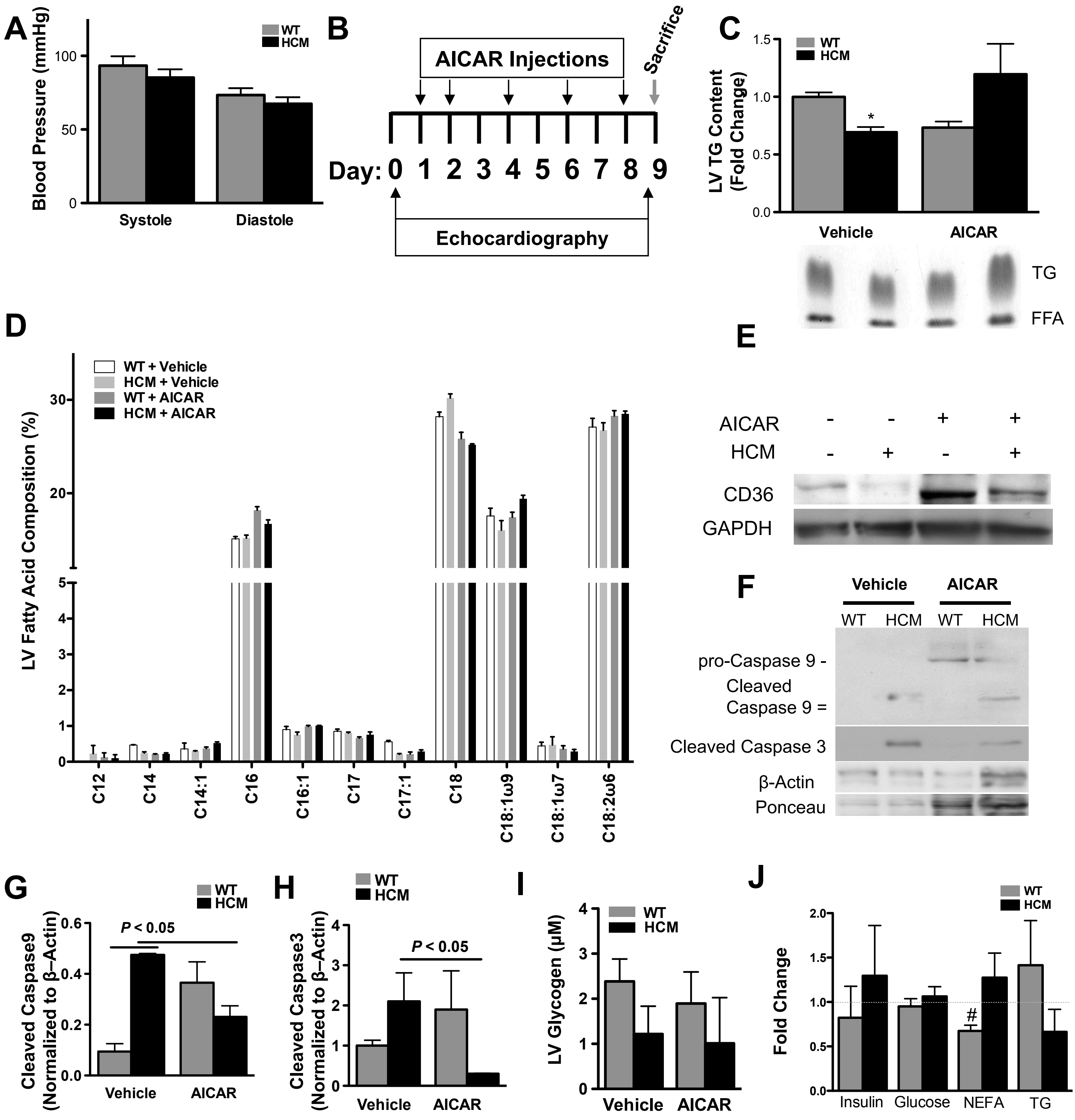

Supplemental Figure 8: The cardioprotective effects of AICAR administration. (A) Blood pressure in 12 month old males. Mean±SEM; *t*-test; *n* = 5-6. (B) Experimental timeline of AICAR injections, measurements, and terminal procedure. (C) Thin-layer chromatography of cardiac lipid extracts from mice administered vehicle or AICAR. *n* = 3. (D) Left ventricular fatty acid profile in WT/HCM mice ±AICAR. Mean±SEM; *ANOVA*; *n* = 3. (E) Immunoblot analysis of CD36 (normalized to GAPDH) in pooled cardiac lysates from mice administered vehicle or AICAR. (F-H) Cardiac caspases3/9 and β-actin immunoblot. Mean±SEM; *ANOVA*; *n*=3. (I) Effect of AICAR on ventricular glycogen levels (normalized to wet tissue weight). Mean±SEM; *ANOVA*; *n*=3-5. (J) Effect of AICAR on post-prandial circulating insulin, glucose, NEFA (non-esterified fatty acid) and TG levels. Values were normalized to pre-injection baseline levels. Mean±SEM; *ANOVA*; *n*=4-9. *Significantly different ($P \leq 0.05$) from WT control and AICAR-treated HCM. [#]Significantly different ($P \leq 0.05$) from WT before AICAR-treatment.
