# Supplementary material for: Metabolic crosstalk between the heart and liver impacts familial hypertrophic cardiomyopathy
Source: EMBO Mol Med. 2014 Feb 24;6(4):482–95. doi: 10.1002/emmm.201302852 (PMC3992075; doi:10.1002/emmm.201302852)
Supplement: Supplementary file 10 [file emmm0006-0482-sd10.pdf]

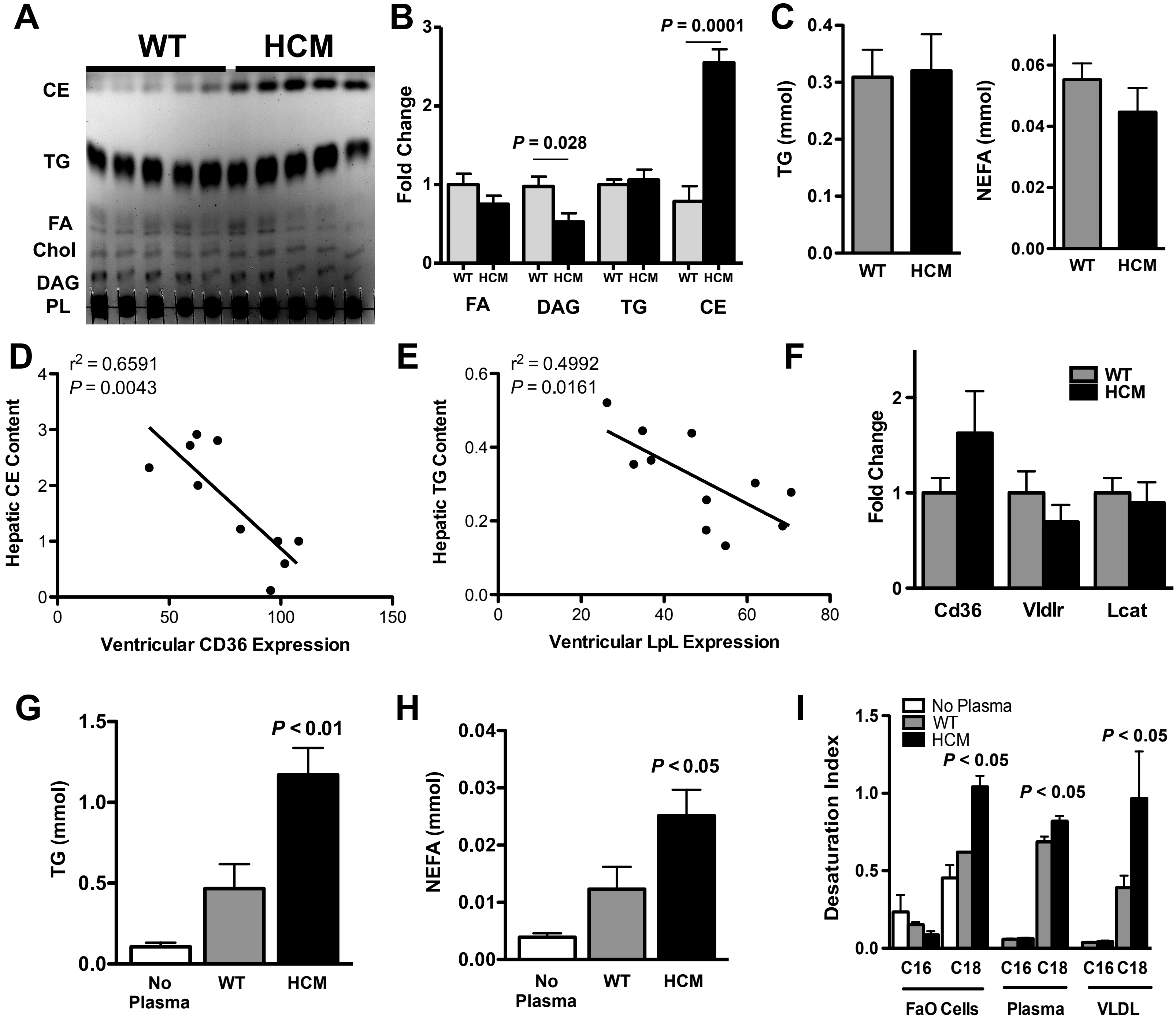

Supplemental Figure 9: Lipid accumulation in the 6 month liver and hepatocytes cultured with plasma from 12 month old HCM males. (A-B) Thin-layer chromatography of hepatic lipid extracts from 6 month old male mice. Mean±SEM; *t-test*; *n*=5. (C) Colorimetric analysis of hepatic TG and non-esterified fatty acid (NEFA) content (normalized to protein) in 6 month old male WT/HCM mice. Mean±SEM; *t-test*; *n*=5-6. (D) Regression analysis of ventricular CD36 transcript levels and hepatic cholesteryl ester (CE) content (measured by TLC as fold change) in 6 month old male WT/HCM mice. (E) Regression analysis of ventricular lipoprotein lipase transcript levels and hepatic TG content (measured by enzymatic assay as mmol/l and normalized to protein) in 6 month old male WT/HCM mice. (F) qPCR measurement of Cd36, Vldlr, and lecithin:cholesterol acyltransferase (Lcat) transcript levels in the 6 month liver. (G-H) Colorimetric analyses of TG and non-esterified fatty acid (NEFA) content in FaO cells cultured with no plasma (n = 2), WT plasma (n = 5) or HCM plasma (n = 5) from 12 month old males. Mean±SEM; *ANOVA.* (I) Desaturation indices for plasma treated cells, and plasma or VLDL at 12 months (in males). Mean±SEM; *ANOVA*; *n*=2-5.
