# Supplementary material for: Metabolic crosstalk between the heart and liver impacts familial hypertrophic cardiomyopathy
Source: EMBO Mol Med. 2014 Feb 24;6(4):482–95. doi: 10.1002/emmm.201302852 (PMC3992075; doi:10.1002/emmm.201302852)
Supplement: Supplementary file 11 [file emmm0006-0482-sd11.pdf]

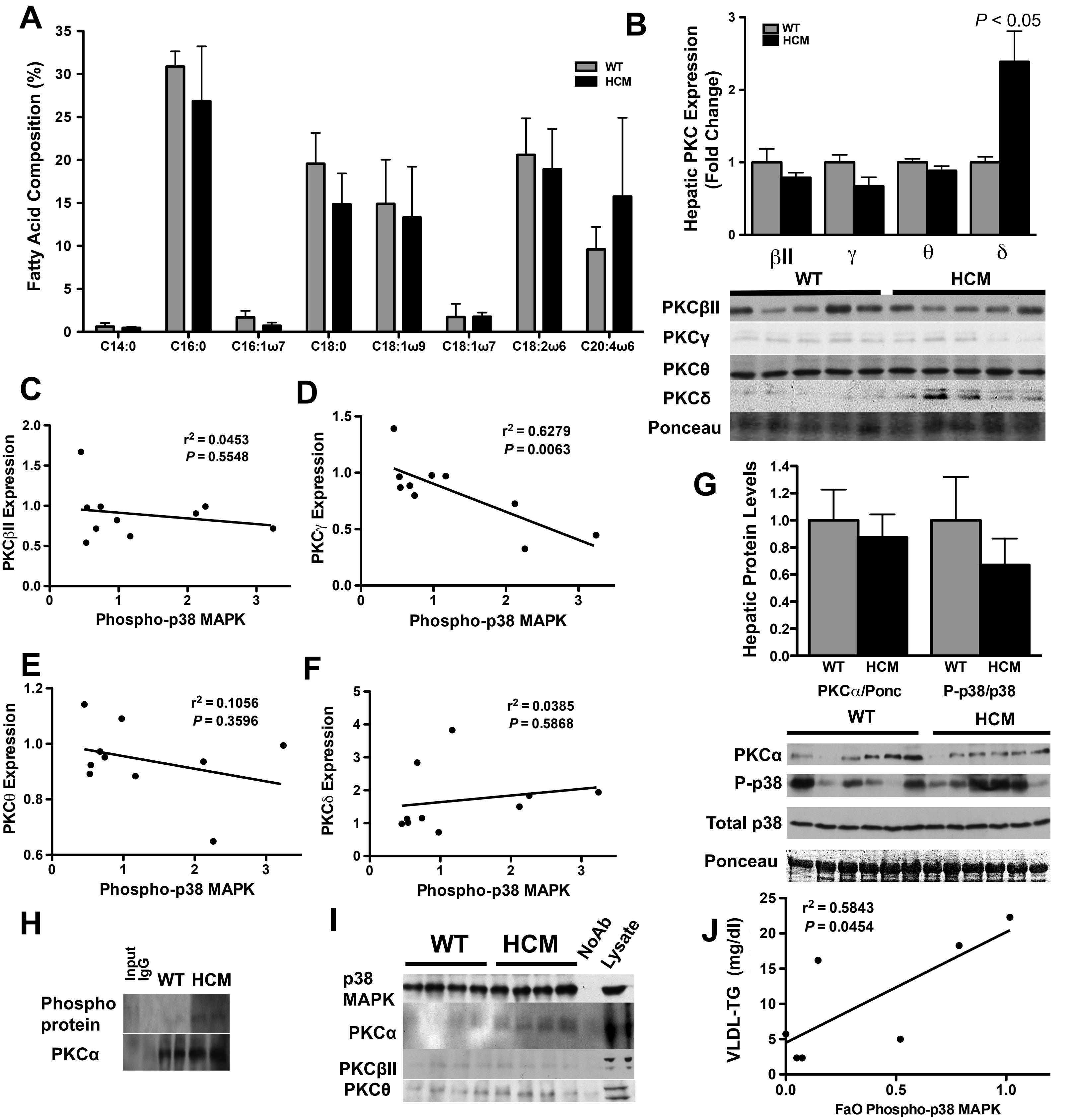

Supplemental Figure 10: Hepatic lipid-sensitive PKC isoforms and HCM. (A) Hepatic fatty acid composition of compensated 2-3 month-old male mice, determined by gas chromatography. Mean±SEM; *t-test*; n = 3-5. (B) Western blot analysis of protein kinase C isoforms in the 12 month liver of males. Mean±SEM; *t-test*; n = 5-6. (C-F) Regression analyses of p38 MAPK phosphorylation and hepatic content of PKCβII/γ/θ/δ at 12 months of age. (G) Western blot analysis of hepatic PKCα (normalized to ponceau staining), phosphorylated and total p38 MAPK protein levels in 6 month males. Mean±SEM; *t-test*; n = 6. (H) Anti-phospho-protein and anti-PKCα western blots of PKCα immunoprecipitated from pooled 12 month liver lysates; whole liver lysate (input) and IgG IP controls. n = 3. (I) Anti-PKCα/βII/θ and anti-p38 western blots of p38 MAPK immunoprecipitated from liver lysates. Mean±SEM; *t-test*; n=4. (J) Regression analysis of cellular (FaO) p38 MAPK phosphorylation and concentration of VLDL triglyceride in plasma added to the culture medium.
