# Supplementary material for: Metabolic crosstalk between the heart and liver impacts familial hypertrophic cardiomyopathy
Source: EMBO Mol Med. 2014 Feb 24;6(4):482–95. doi: 10.1002/emmm.201302852 (PMC3992075; doi:10.1002/emmm.201302852)
Supplement: Supplementary file 12 [file emmm0006-0482-sd12.pdf]

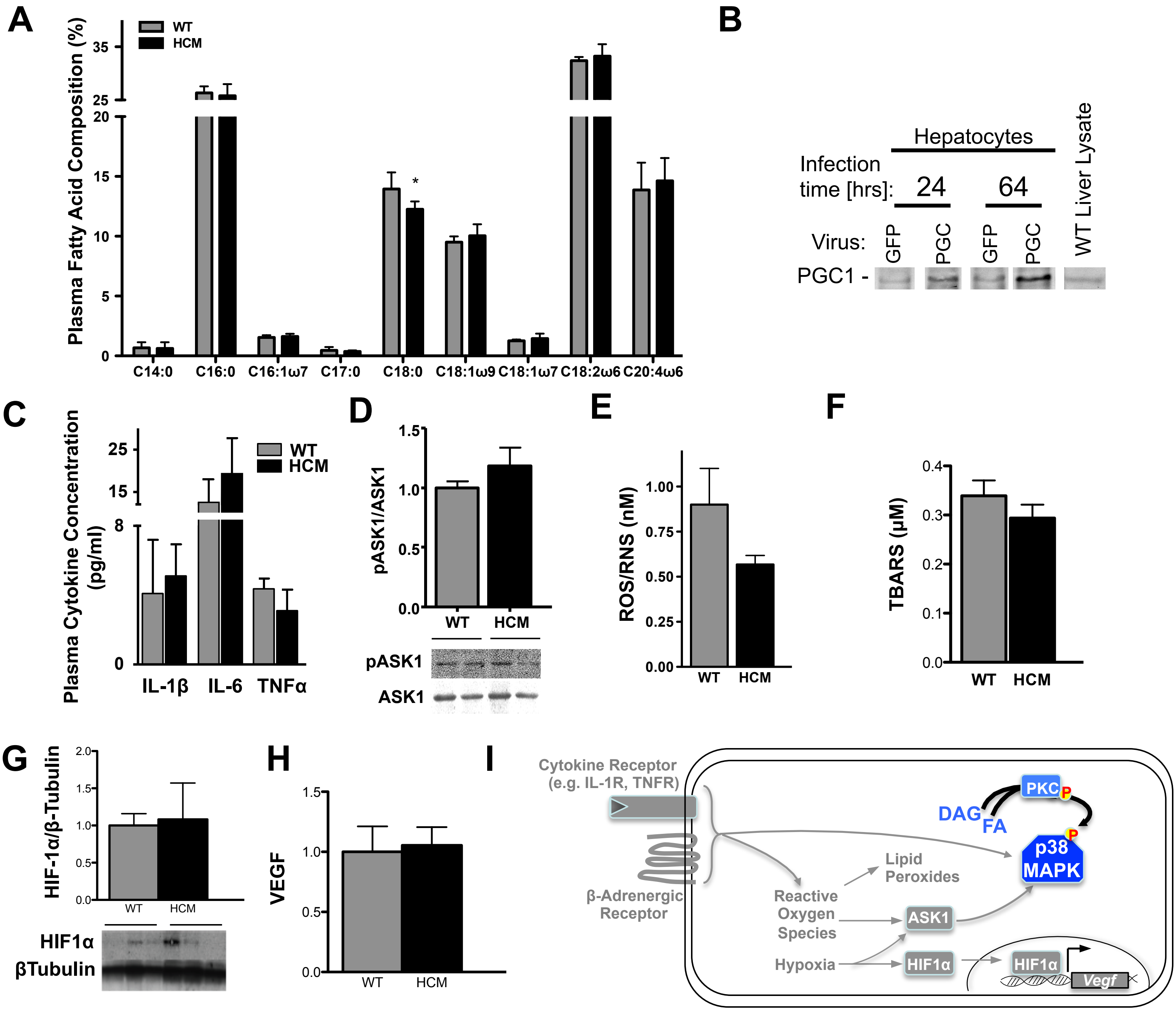

Supplemental Figure 11: Hepatic p38 MAPK activity associated with HCM is not due to inflammatory, oxidative or hypoxic stress. (A) Fatty acid composition of whole plasma from fasted 12 month old males. Mean±SEM; *t-test*; *n* = 5. (B) PGC1 immunoblot of FaO cell lysates (and liver control) following (24 or 64 hour) infection by GFP or PGC1-expressing adenovirus. (C) ELISA determination of circulating inflammatory cytokines; interleukin 1β (IL-1β), interleukin 6 (IL-6) and tumor necrosis factor α (TNFα). Mean±SEM; *t-test*; *n*=4. (D) Immunoblot analysis of phosphorylated and total apoptosis signaling kinase-1 (ASK-1) in the 12 month liver. Mean±SEM; *t-test*; *n*=5-7. (E) Measurement of hepatic reactive oxygen species (ROS) in 12 month old males. Normalized to tissue protein content. Mean±SEM; *t-test*; *n* = 3-4. (F) Measurement of plasma lipid peroxides (TBARS, thiobarbituric acid reactive substances) in 12 month wildtype and HCM mice. Mean±SEM; *t-test*; *n* = 4. (G) Immunoblot analysis of steady-state hypoxia inducible factor 1α (HIF1α) levels in the 12 month livers of wildtype and HCM males. Loading was normalized to β-tubulin. Mean±SEM; *t-test*; *n* = 5. (H) qPCR of HIF1α target vascular endothelial growth factor (Vegf) in the 12 month livers of wildtype and HCM males. Mean±SEM; *t-test*; *n* = 5. (I) Diagram of possible routes to PKC and p38 MAPK activation; Blue represents observed increases, Grey represents unaltered hepatic components. * $p < 0.05$, significantly different from wildtype.
