# Supplementary material for: Metabolic crosstalk between the heart and liver impacts familial hypertrophic cardiomyopathy
Source: EMBO Mol Med. 2014 Feb 24;6(4):482–95. doi: 10.1002/emmm.201302852 (PMC3992075; doi:10.1002/emmm.201302852)
Supplement: Supplementary file 13 [file emmm0006-0482-sd13.pdf]

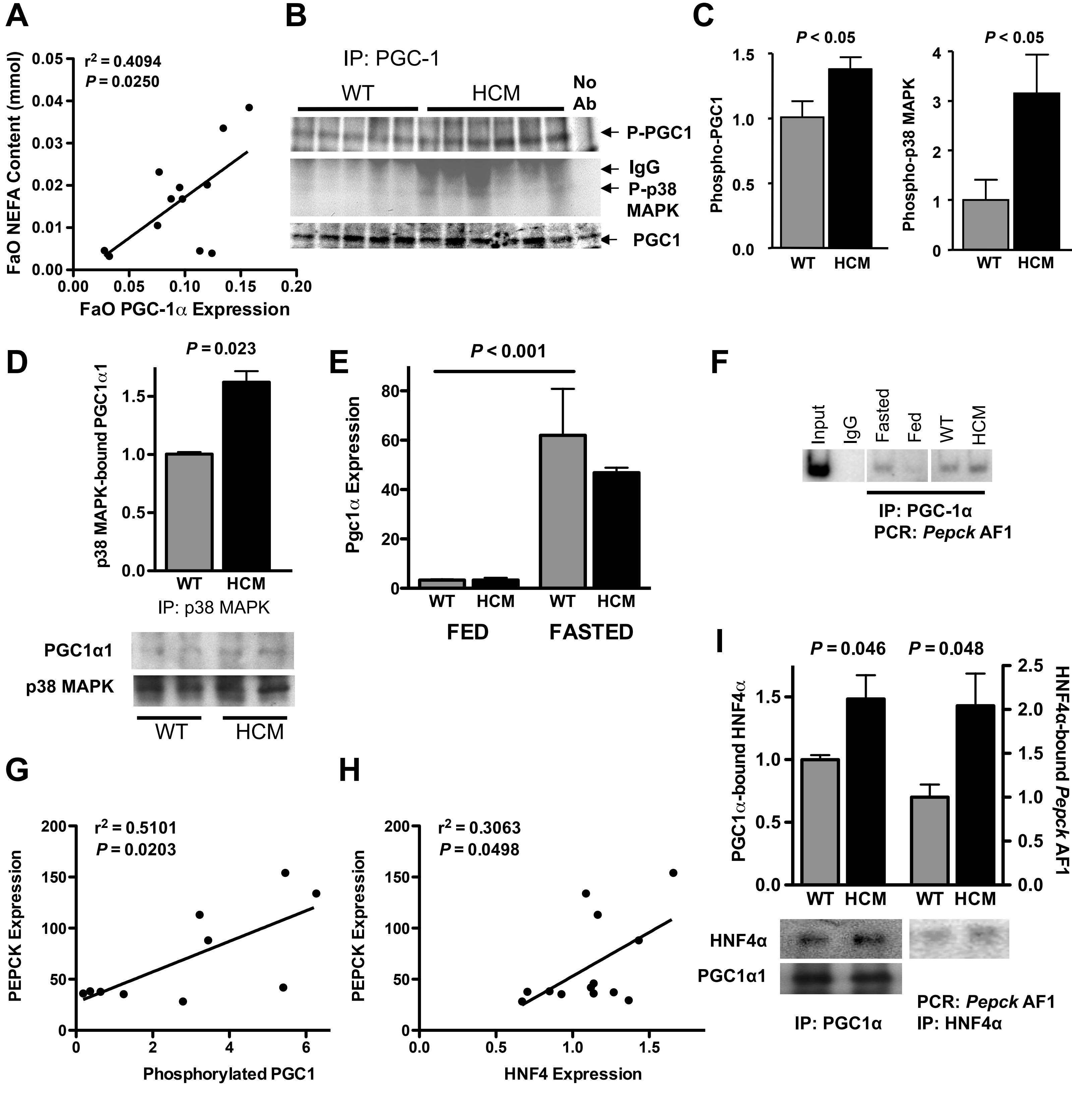

Supplemental Figure 12: PGC-1α activity in the HCM liver. (A) Regression analysis of plasma-induced intracellular non-esterified fatty acid (NEFA) accumulation and PGC-1α protein expression in FaO hepatocytes. (B-C) Western blot analysis of PGC-1 co-immunoprecipitation in the 12 month liver of males; pan-phosphoprotein, phospho-p38 MAPK, PGC-1. Mean±SEM; *t-test*; *n* = 6-8. (D) Western blot analysis of p38 MAPK co-immunoprecipitation in the 12 month liver of males; p38 MAPK and PGC-1. Mean±SEM; *t-test*; *n* = 4. (E) qPCR of PGC-1α expression in the fed or fasted 12 month male liver. Mean±SEM; *t-test*; *n*=4. (F) Anti-PGC1 chromatin immunoprecipitation of the *Pepck* promoter. n = 5. (G-H) Regression analyses of PEPCK expression versus (G) phosphorylated PGC-1α protein or (H) HNF4α expression in the 12 month male liver. (I) Co-immunoprecipitation of PGC-1α/HNF-4α and anti-HNF4α chromatin immunoprecipitation of the *Pepck* promoter. Mean±SEM; *t-test*; *n*=4-5.
