# Supplementary material for: Metabolic crosstalk between the heart and liver impacts familial hypertrophic cardiomyopathy
Source: EMBO Mol Med. 2014 Feb 24;6(4):482–95. doi: 10.1002/emmm.201302852 (PMC3992075; doi:10.1002/emmm.201302852)
Supplement: Supplementary file 14 [file emmm0006-0482-sd14.pdf]

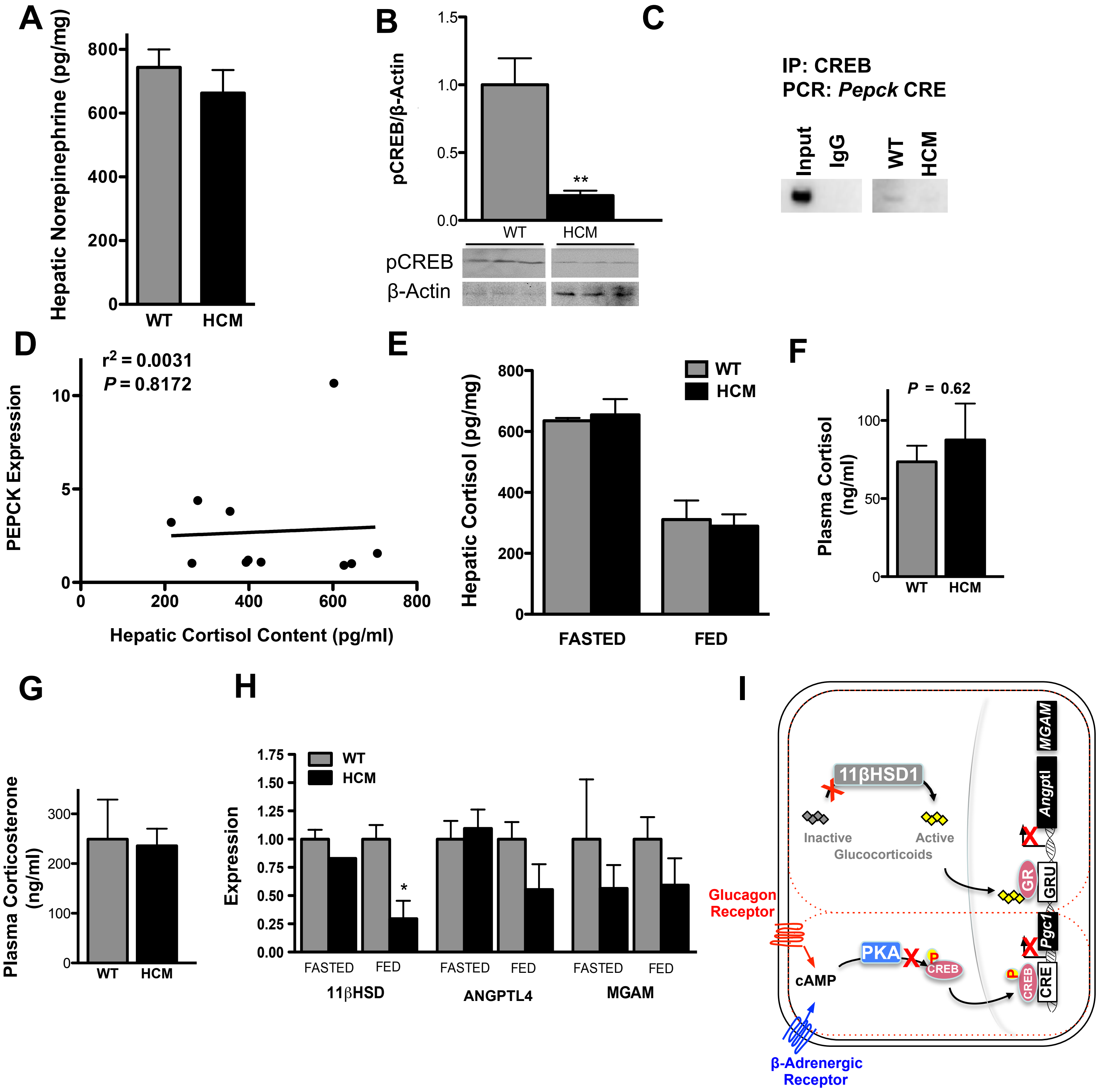

Supplemental Figure 13: Absence of adrenergic, glucagon, and glucocorticoid receptor activity in 12 month-old males. (A) Hepatic norepinephrine levels (normalized to tissue weight). Mean±SEM; *t-test*; *n* = 4-6. (B) Western blot of hepatic phospho-cAMP-responsive element binding protein (CREB). Normalized to β-actin. Mean±SEM; *t-test*; *n* = 6. (C) Chromatin immunoprecipitation of hepatic CREB-bound cAMP-responsive element (CRE) of the *Pepck* promoter. *n* = 5. (D) Regression analysis of hepatic PEPCK expression and cortisol content. (E) Hepatic cortisol content. Mean±SEM; *t-test*; *n* = 2 (fast) or 4 (fed). (F-G) Plasma cortisol and corticosterone levels. Mean±SEM; *t-test*; *n* = 6-7. (H) qPCR of local glucocorticoid regulator 11-β hydroxysteroid dehydrogenase 1 (11βHSD1), and primary glucocorticoid-responsive genes angiopoietin-related protein 4 (ANGPTL4) and maltase-glucoamylase (MGAM) in the fasted or fed liver. Mean±SEM; *t-test*; *n* = 5-6. (I) Diagram detailing the activation of the cAMP-responsive element (CRE) and glucocorticoid-responsive unit (GRU) by adrenergic/glucagon receptors or glucocorticoid receptor, respectively. *p < 0.05, **Significantly different ($P \leq 0.01$) from WT control.
