# Supplementary material for: Metabolic crosstalk between the heart and liver impacts familial hypertrophic cardiomyopathy
Source: EMBO Mol Med. 2014 Feb 24;6(4):482–95. doi: 10.1002/emmm.201302852 (PMC3992075; doi:10.1002/emmm.201302852)
Supplement: Supplementary file 15 [file emmm0006-0482-sd15.pdf]

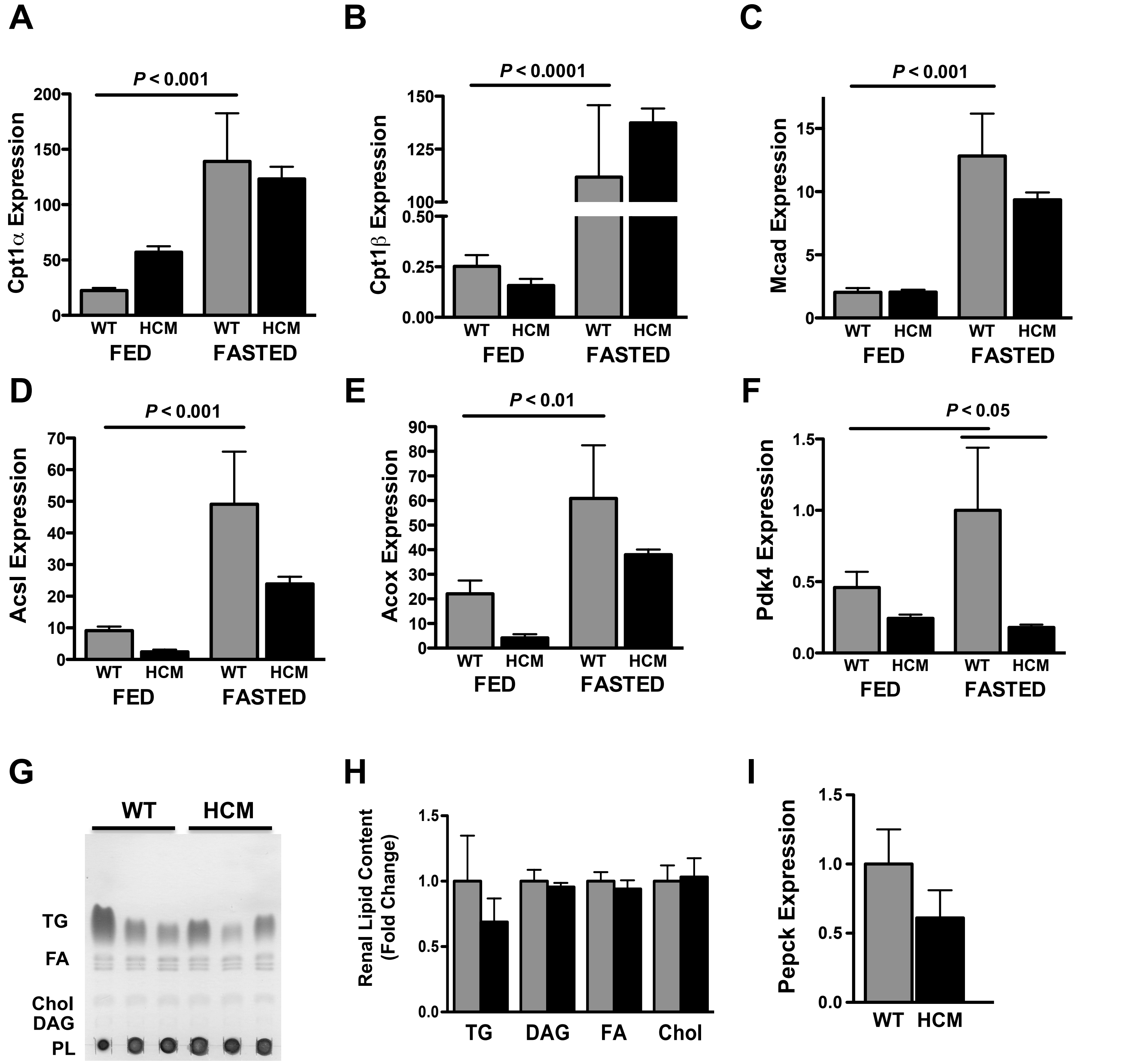

Supplemental Figure 14: Absence of hepatic peroxisome proliferator-activated receptor-α target activation or renal lipid accumulation and PEPCK upregulation in 12 month-old males. (A-F) Expression analysis of canonical PPARα targets in the liver; (A) Liver and (B) muscle-type carnitine palmitoyl transferase-1, (C) Medium-chain acyl CoA dehydrogenase, (D) Long-chain acyl CoA synthase, (E) Acyl CoA oxidase, (F) Pyruvate dehydrogenase kinase-4. Determined by qPCR. Mean±SEM; *ANOVA*; *n* = 4-8. (G-H) TLC of renal lipid extracts. Mean±SEM; *t-test*; *n* = 3. (I) Renal PEPCK expression. Mean±SEM; *t-test*; *n* = 5.
