# Supplementary material for: Metabolic crosstalk between the heart and liver impacts familial hypertrophic cardiomyopathy
Source: EMBO Mol Med. 2014 Feb 24;6(4):482–95. doi: 10.1002/emmm.201302852 (PMC3992075; doi:10.1002/emmm.201302852)
Supplement: Supplementary file 16 [file emmm0006-0482-sd16.pdf]

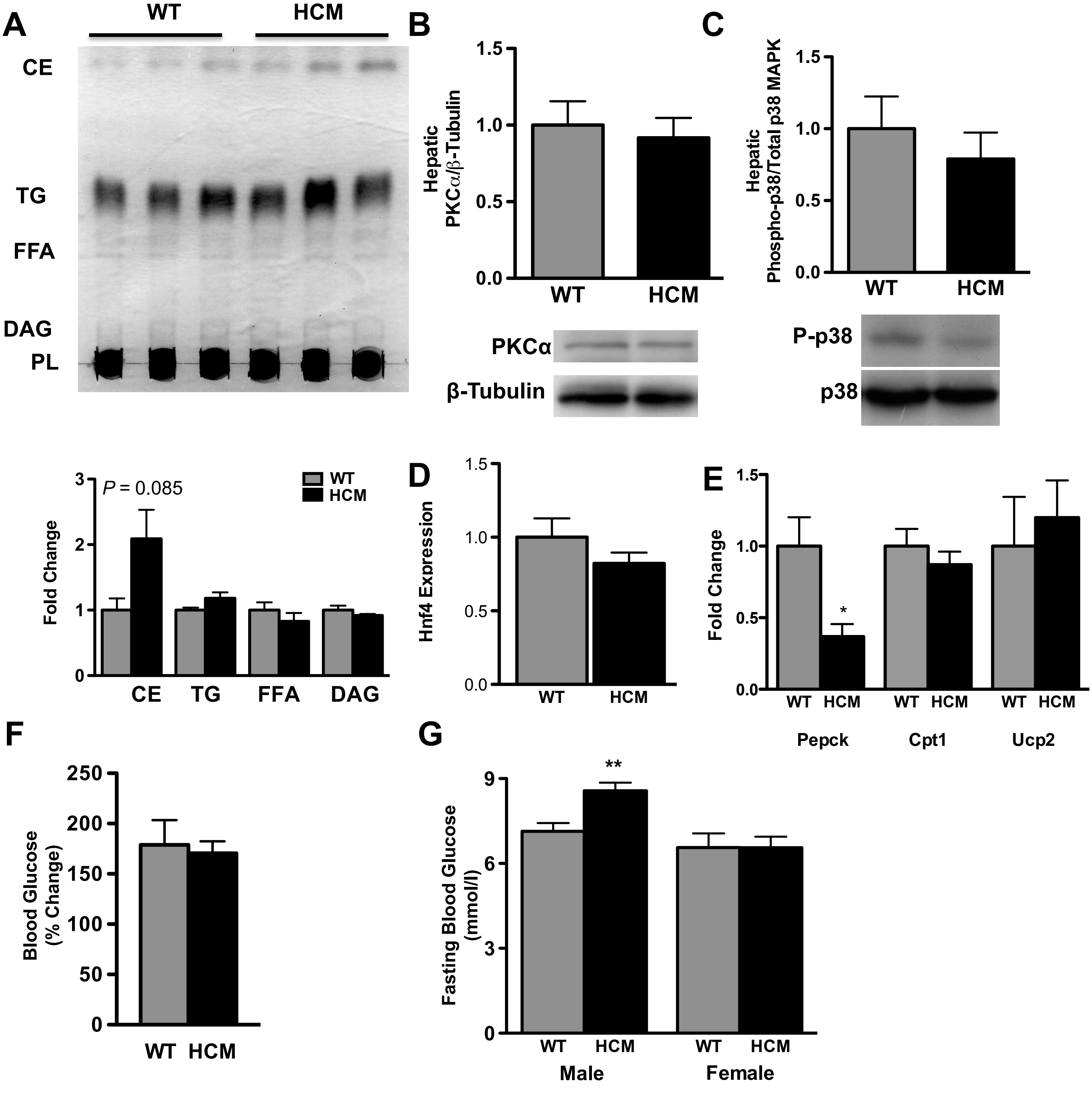

Supplemental Figure 15: Hepatic lipid accumulation, p38 MAPK activation and PEPCK upregulation is absent in age-matched HCM females. (A) Thin-layer chromatography of hepatic lipid extracts from 12 month old females. Mean±SEM; *t-test*; *n* = 3. (B) Immunoblot analysis of hepatic PKCα expression (normalized to β-tubulin) in 12 month old females. Mean±SEM; *t-test*; *n* = 3. (C) Immunoblot analysis of hepatic p38 MAPK phosphorylation in 12 month old females. Mean±SEM; *t-test*; *n* = 3. (D) qPCR of Hnf-4α transcript levels in the livers of 12 month old females. Mean±SEM; *t-test*; *n* = 5. (E) qPCR of PGC-1α target levels in the livers of 12 month old females. Mean±SEM; *t-test*; *n* = 5. (F) Percent increase in blood glucose levels in 12 month old females 15 minutes after pyruvate injection Mean±SEM; *t-test*; *n* = 4. (G) Male and female fasting blood glucose levels. Mean±SEM; *ANOVA* ; *n*=7-16. * p < 0.05, significantly different from wildtype ; ** p < 0.05, significantly different from wildtype males and HCM females.
