# Supplementary material for: Metabolic crosstalk between the heart and liver impacts familial hypertrophic cardiomyopathy
Source: EMBO Mol Med. 2014 Feb 24;6(4):482–95. doi: 10.1002/emmm.201302852 (PMC3992075; doi:10.1002/emmm.201302852)
Supplement: Supplementary file 17 [file emmm0006-0482-sd17.pdf]

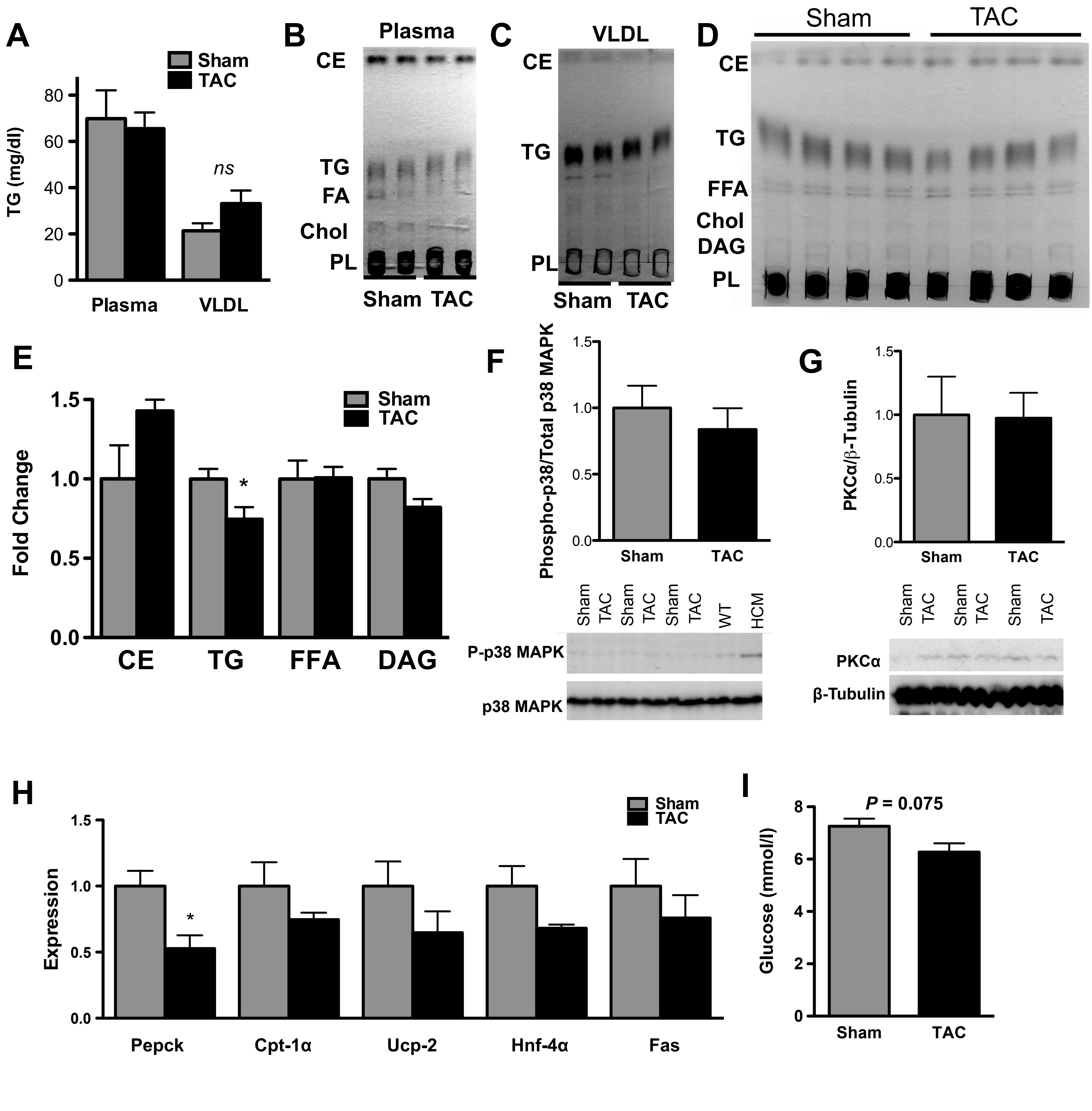

Supplemental Figure 16: Hepatic metabolism 8-10 weeks after sham surgery or transverse aortic constriction (TAC). (A) Colorimetric determination of plasma and VLDL TG from sham and TAC operated mice. Mean±SEM; *t*-test; *n* = 4-9. (B-C) Thin-layer chromatography of plasma and VLDL lipid extracts from sham and TAC mice. *n* = 4. (D-E) TLC of hepatic lipid extracts. Mean±SEM; *t*-test; *n*=5. (F) Western blot analysis of hepatic p38 MAPK phosphorylation (normalized to total p38 MAPK). Mean±SEM; *t*-test; *n* = 3. (G) Western blot analysis of hepatic PKCα levels (normalized to β-tubulin). Mean±SEM; *t*-test; *n* = 3. (H) qPCR analysis of Pepck, liver-type carnitine palmitoyltransferase-1 (Cpt-1α), uncoupling protein-2 (Ucp-2), hepatocyte nuclear factor-4 (Hnf-4α), and fatty acid synthase (Fas) expression. Mean±SEM; *t*-test; *n*=4. (I) Circulating blood glucose levels. Mean±SEM; *t*-test; *n*=5-9. *Significantly different ($P \leq 0.05$) from sham control.
