# Supplementary material for: Metabolic crosstalk between the heart and liver impacts familial hypertrophic cardiomyopathy
Source: EMBO Mol Med. 2014 Feb 24;6(4):482–95. doi: 10.1002/emmm.201302852 (PMC3992075; doi:10.1002/emmm.201302852)
Supplement: Supplementary file 18 [file emmm0006-0482-sd18.pdf]

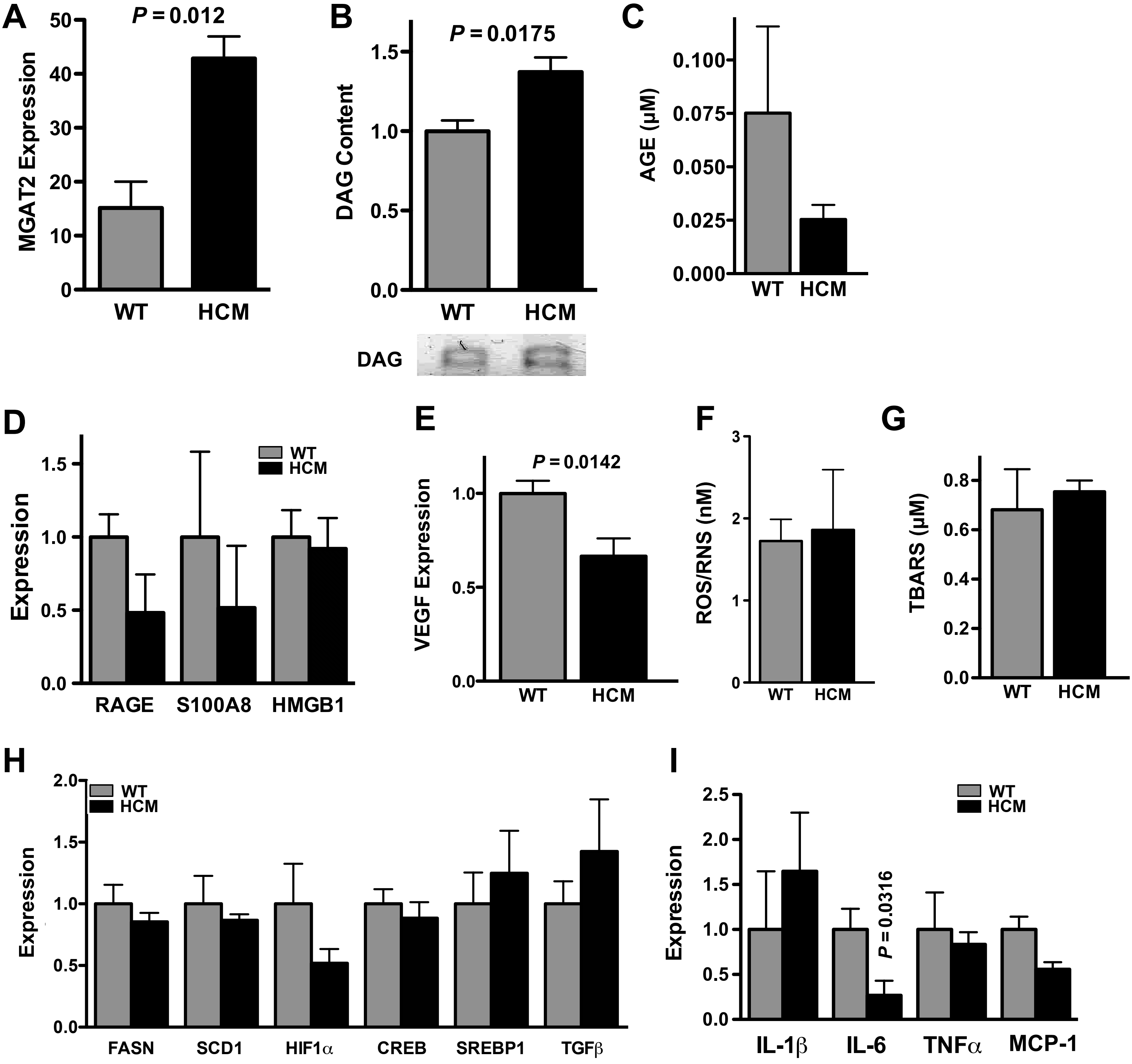

Supplemental Figure 17: Markers of glucotoxicity in the left ventricle of 12-15 month old HCM males. (A) qPCR of MGAT2 expression. Mean±SEM; *t-test*; *n* = 3. (B) Diacylglycerol (DAG) content measured by thin-layer chromatography (normalized to tissue weight). Mean±SEM; *t-test*; *n* = 3. (C) Advanced glycated endproduct (AGE) content (normalized to protein). Mean±SEM; *t-test*; *n* = 4-5. (D) qPCR of receptor for advanced glycated endproduct (RAGE), calprotectin (S100A8) and high mobility group box-1 (HMGB1). Mean ±SEM; *t-test*; *n* = 5. (E) qPCR of the hypoxia-inducible factor-1 target vascular endothelial growth factor (VEGF). Mean±SEM; *t-test*; *n* = 3. (F) Measurement of tissue reactive oxygen/nitrogen species (ROS/RNS) (normalized to protein). Mean±SEM; *t-test*; *n* = 3. (G) Measurement of tissue lipid peroxides (thiobarbituric acid reactive substances, TBARS) (normalized to protein). Mean±SEM; *t-test*; *n* = 3. (H-I) Expression of glucose-regulated genes; interleukins (IL-1β and IL-6), tumor necrosis factor-α (TNFα) and monocyte chemoattractant protein-1 (MCP-1). Measured by qPCR. Mean±SEM; *t-test*; *n* = 3-5.
