# Supplementary material for: Metabolic crosstalk between the heart and liver impacts familial hypertrophic cardiomyopathy
Source: EMBO Mol Med. 2014 Feb 24;6(4):482–95. doi: 10.1002/emmm.201302852 (PMC3992075; doi:10.1002/emmm.201302852)
Supplement: Supplementary file 19 [file emmm0006-0482-sd19.pdf]

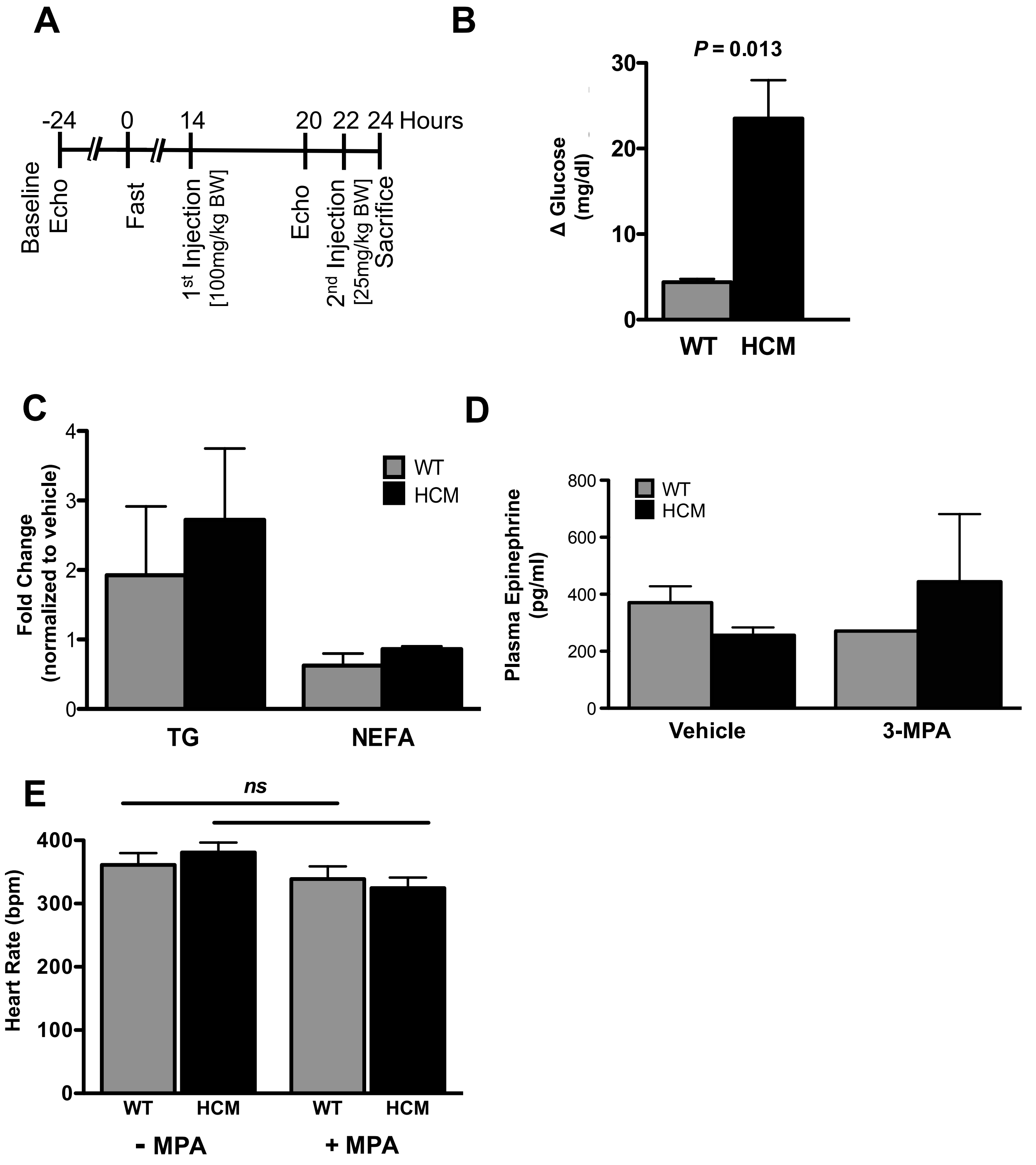

Supplemental Figure 18: Inhibition of gluconeogenesis in HCM mice. (A) Timeline of 3-MPA injections and measurements. (B) Change in blood glucose levels with 3-MPA. Normalized to levels preceding 3-MPA injection. Mean±SEM; *t-test; n*=3. (C) Enzymatic determination of plasma TG and non-esterified fatty acids (NEFA) following 3-MPA administration (normalized to values from mice injected with vehicle only). Mean±SEM; *t-test; n*=3. (D) Plasma epinephrine after vehicle or 3-MPA treatment; determined by ELISA. Mean±SEM; *ANOVA; n*=3. (E) Heart rate after vehicle or 3-MPA administration; determined by echocardiography. Mean±SEM; *ANOVA; n*=8-12.
