# Supplementary material for: Metabolic crosstalk between the heart and liver impacts familial hypertrophic cardiomyopathy
Source: EMBO Mol Med. 2014 Feb 24;6(4):482–95. doi: 10.1002/emmm.201302852 (PMC3992075; doi:10.1002/emmm.201302852)
Supplement: Supplementary file 20 [file emmm0006-0482-sd20.pdf]

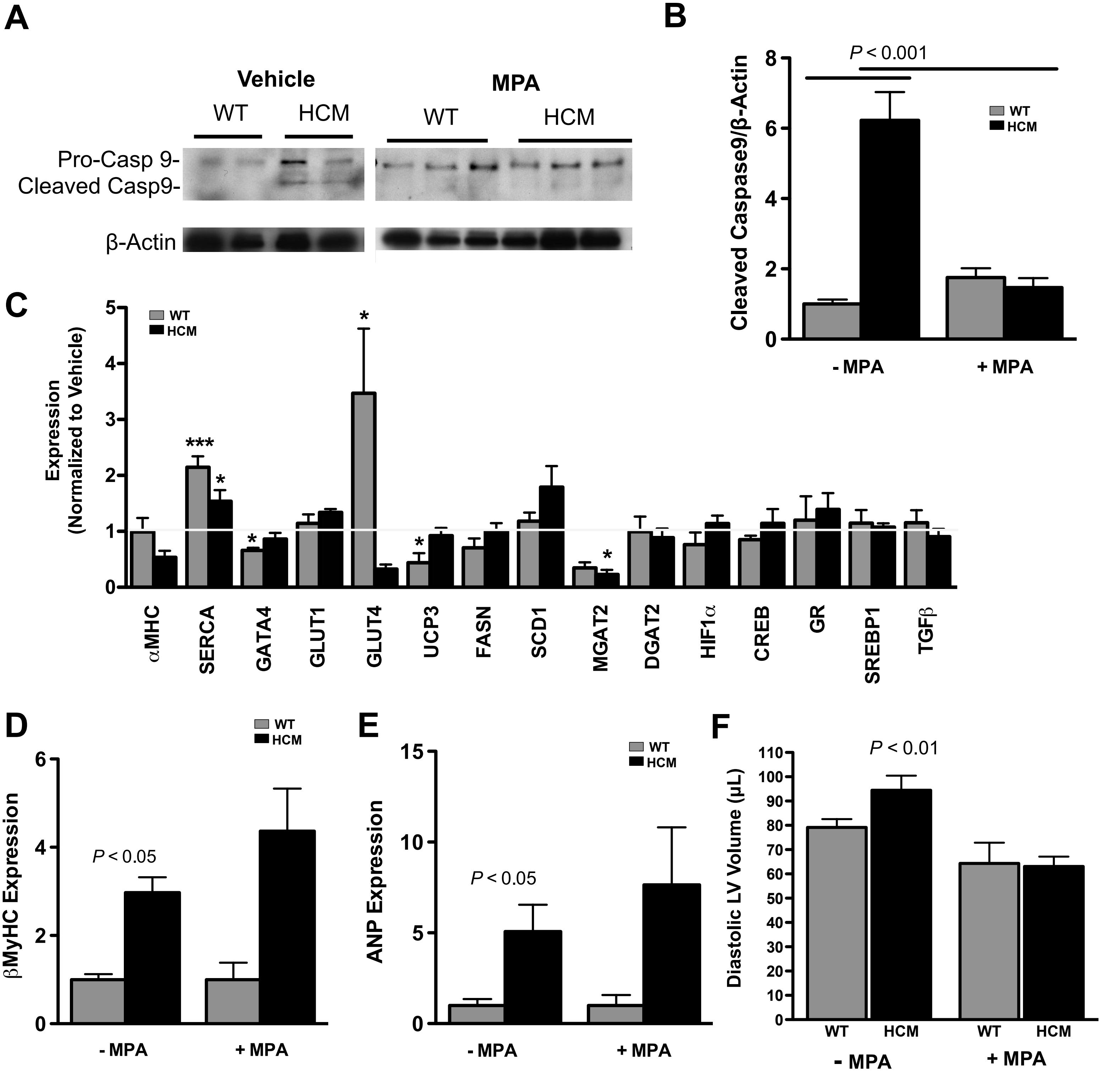

Supplemental Figure 19: Inhibition of gluconeogenesis allows for partial rescue of cardiac disease phenotype. (A-B) Western blot analysis of left ventricular caspase9 levels following 3-MPA or vehicle administration. Normalized to β-actin. Mean±SEM; *t*-test; *n*=3. (C) qPCR expression panel of glucose-regulated genes in ventricles following 3-MPA or vehicle administration. α-Myosin heavy chain (MHC), sarcoplasmic reticulum calcium ATPase (SERCA), glucose transporter (GLUT-1/4), uncoupling protein (UCP-3), fatty acid synthase (FASN), stearoyl CoA desaturase (SCD-1), monoacylglycerol acyltransferase (MGAT-2), diacylglycerol acyltransferase (DGAT-2), hypoxia inducible factor (HIF-1α), cAMP response element binding protein (CREB), glucagon receptor (GR), sterol response element binding protein (SREBP-1), transforming growth factor (TGFβ). Mean±SEM; *ANOVA*; *n*=3. (D-E) Left ventricular β-myosin and ANP transcript levels (determined by qPCR). Mean±SEM; *ANOVA*; *n*=3-5. (F) Echocardiographic determination of diastolic left ventricular volume. Mean ±SEM; *ANOVA*; *n*=8-12. *Statistically significant ($P < 0.05$), ***($P < 0.005$) relative to vehicle-injected mice.
