# Supplementary material for: Metabolic crosstalk between the heart and liver impacts familial hypertrophic cardiomyopathy
Source: EMBO Mol Med. 2014 Feb 24;6(4):482–95. doi: 10.1002/emmm.201302852 (PMC3992075; doi:10.1002/emmm.201302852)
Supplement: Supplementary file 21 [file emmm0006-0482-sd21.pdf]

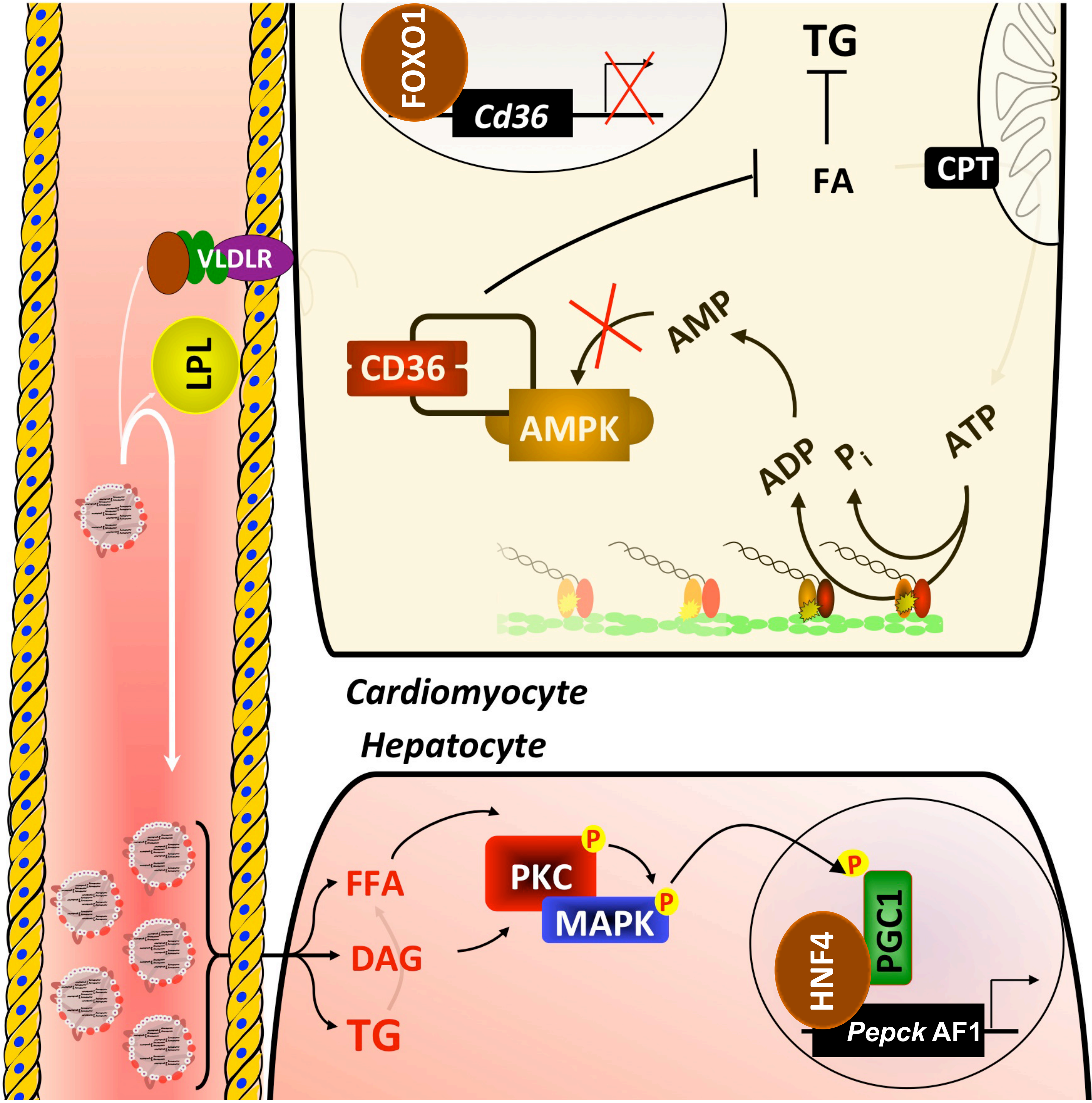

Supplemental Figure 20: Hepatic response to genetic heart disease modulates contractile function. The progression of genetic heart disease is accompanied by a concerted inhibition of lipid utilization by the heart. Normalization of lipid utilization by activating AMPK rescues the HCM heart from cardiac dysfunction. Deprived of sufficient AMPK or CD36 activity, VLDL triglycerides are not cleared by the diseased heart. Triglyceride, diacylglycerol and oleic acid subsequently accumulate in the livers of HCM mice, where lipid-sensitive intracellular signaling pathways – PKC and MAPK – are activated and upregulate Pepck. Accordingly, HCM mice exhibit elevated fasting glucose levels, and are rescued from cardiac dysfunction upon inhibition of PEPCK by 3-MPA.
