# Supplementary material for: Metabolic crosstalk between the heart and liver impacts familial hypertrophic cardiomyopathy
Source: EMBO Mol Med. 2014 Feb 24;6(4):482–95. doi: 10.1002/emmm.201302852 (PMC3992075; doi:10.1002/emmm.201302852)
Supplement: Supplementary file 22 [file emmm0006-0482-sd22.pdf]

Supporting Information Table 1A

Table 1A: Pathological Cardiac Metabolism

		Hypertrophic Cardiomyopathy Familial		Hypertrophic Cardiomyopathy Idiopathic (Human)	Pressure/Volume-Overload or Hypertension	Tachy-cardia (Pacing)	Myocardial Infarction and Ischemia	General Heart Failure (Human)	References
		Human	R403Q Males						
Myocardium	TG Content		↓	↓	↓	↑	↑	↑	2-5,9,10
	ATP Content		↓	↓	↓	↓		=	11,12,16,26
	ADP Content		↑		↑				13,16
	CD36 Activity/Content		↓	↓	=/↑				14
	AMPK Activity		↓		↑	↑	↑		15-19
	Inflammation		=		↑			↑	5,67
	ROS Content		=		↑	↑	↑	↑	50,67
Plasma	TG		↑		=/↑				5,8
	NEFA		=		↑	↑		↑	5,10,31
	Cholesterol		=		↑			↑	8,22
	Glucose		↑	↑	=/↑↓	↑	=	↑	8,10,23,24,33
	Catecholamines		=	=	↑	↑		↑	5-7,10,27,31
	Inflam. Cytokines		=	↑	↑		↑	↑	8,20,21,25,32
Liver	TG Content		↑		=/↑				5,8
	Kinase Activity		↑		=				
	FASN Expression		=		=/↑				8
	PEPCK Expression		↑		↓				8
	Body Weight or BMI		=		↓			↑	8,22

Key: Triglyceride (TG), non-esterified fatty acids (NEFA), fatty acid synthase (FASN), phosphoenolpyruvate carboxykinase (PEPCK)
