# Supplementary material for: Metabolic crosstalk between the heart and liver impacts familial hypertrophic cardiomyopathy
Source: EMBO Mol Med. 2014 Feb 24;6(4):482–95. doi: 10.1002/emmm.201302852 (PMC3992075; doi:10.1002/emmm.201302852)
Supplement: Supplementary file 23 [file emmm0006-0482-sd23.pdf]

Supporting Information Table 1B

Table 1B: Sexually Dimorphic Metabolic Changes

		Hypertrophic Cardiomyopathy	
		Males	Females
Myocardium	Function (%ES)	↓	=
	LV Volume	↓	=
	TG Content	↓	↓
	CD36 Expression	↓	=
	AMPK Activity	↓	=
Plasma	TG	↑	= ↓
	Glucose	↑	=
Liver	TG Content	↑	=
	Kinase Activity	↑	=
	PEPCK Expression	↑	↓
	Glucose Production	↑	=
