# Supplementary figures and images for: Metabolic crosstalk between the heart and liver impacts familial hypertrophic cardiomyopathy
Source: EMBO Mol Med. 2014 Feb 24;6(4):482–95. doi: 10.1002/emmm.201302852 (PMC3992075; doi:10.1002/emmm.201302852)

Supporting Information Table 1C

Table 1C: Time-course of Metabolic Changes in HCM Males

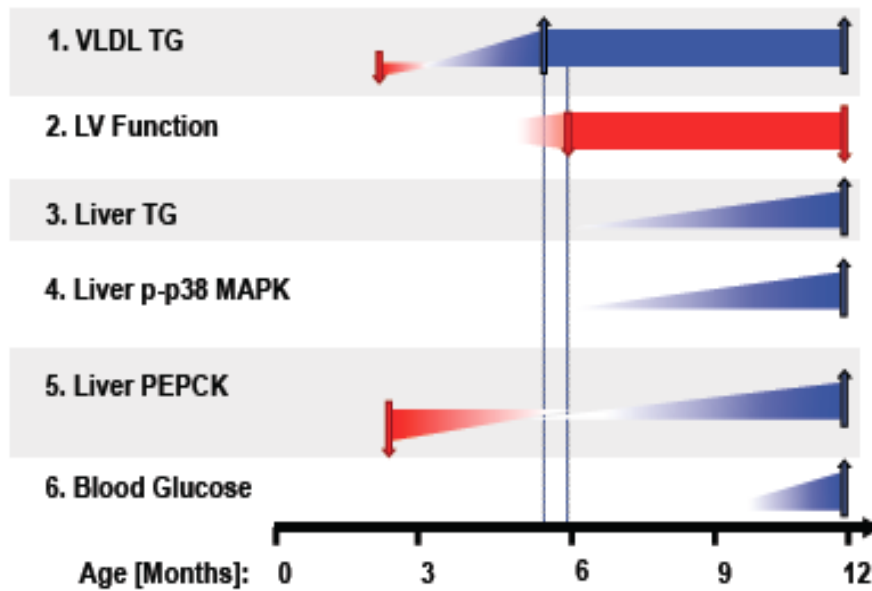

Supplement: Supplementary file 24 [file emmm0006-0482-sd24.pdf]
