# Supplementary material for: Metabolic crosstalk between the heart and liver impacts familial hypertrophic cardiomyopathy
Source: EMBO Mol Med. 2014 Feb 24;6(4):482–95. doi: 10.1002/emmm.201302852 (PMC3992075; doi:10.1002/emmm.201302852)
Supplement: Supplementary file 25 [file emmm0006-0482-sd25.pdf]

Supporting Information Table 2

Table 2: Pharmacological Interventions Targeting AMPK

Model	AMPK Activity	Drug	Benefits	Notes	Ref.	Additional Targets
Ischemia/Reperfusion and Myocardial Infarction (Coronary occlusion)	↑	AICAR Metformin	Reduced infarct size and rescued dysfunction	-AMPK already activated by I/R<br>-No improvement of ventricular dysfunction or dilation<br>-Reduced failure-induced hyperinsulinemia, TNF exp.	15,18,23 28,37-40	-Adenosine receptor (M/A)<br>-Na⁺/H⁺ Exchanger (A)<br>-AKT, ERK<br>-Hyperinsulinemia (M/A)<br>-Inflammation (M)<br>-Nitric oxide synthase (M)<br>-Elevated plasma fatty acids (M/A)<br>-ETC Complex I (M)<br>-Endothelial ROS production (M)<br>-TGFβ signaling in fibroblasts (M)<br>-Body weight (M)<br>-Gluconeogenesis (M)<br>-mTOR activity (M)
Pressure-overload (Transverse aortic constriction)	↑	AICAR Metformin	Normalized blood pressure, fractional shortening and dilation	-AMPK already activated by banding alone<br>-Reduced failure-induced increases in ANP and inflammation	29,36	
Tachycardia-induced Dilated cardiomyopathy (Pacing)	↑	AICAR Metformin	Normalized cardiac output, vascular resistance, hyperinsulinemia, and body fat	-AMPK already activated by pacing alone<br>-Decrease of elevated catecholamines<br>-Increased depressed physical activity	19,30	
Volume-overload (Aortocaval fistula)	=	Metformin	None	-Decreased glucose oxidation<br>-No effect on AMPK activity	34	
Familial Hypertrophic Cardiomyopathy (R403Q αMHC)	↓	AICAR	-Restored LV function, architecture, lipid content<br>-Reduced pathological gene expression	-Restored deficient AMPK activity<br>-No effect on circulating lipids, glucose, ketones		

Key: Metformin (M), 5-Amino-4-imidizole-1-β-D-carboxamide ribofuranoside/AICAR (A), electron transport chain (ETC)
