# Supplementary material for: Metabolic crosstalk between the heart and liver impacts familial hypertrophic cardiomyopathy
Source: EMBO Mol Med. 2014 Feb 24;6(4):482–95. doi: 10.1002/emmm.201302852 (PMC3992075; doi:10.1002/emmm.201302852)
Supplement: Supplementary file 26 [file emmm0006-0482-sd26.pdf]

Supporting Information Table 3

Table 3: Pharmacological Interventions Suppressing Downregulated Substrate Utilization

Drug	Inhibits	Disease/Model	Target Activity	Benefits	Notes	Additional Targets	Ref.
**Perhexiline**	FAO: CPT-1 CPT-2	-Ischemic and Idiopathic Cardiomyopathy	↓	-Improved contractile function and pCr:ATP ratio<br>-Reduced plasma FA,glucose, and insulin	-Efficacy may depend on use with other drugs (e.g. β-blocker)<br>-Coronary vasodilator	-NAD(P)H oxidase<br>-Inflammation<br>-Nitric oxide production<br>-Ca^{2+} channel	31, 41-45
**Trimetazidine**	FAO: 3-keto-thiolase	-Pressure-overload<br>-Ischemic and Idiopathic Cardiomyopathy	↓	-Improved contractile function<br>-Increased HDL and reduced glucose/insulin levels<br>-Reduced fibrosis and ROS	-Functional benefits derived from rescuing nitric oxide synthase activity, rather than directly inhibiting cardiac FAO<br>-FAO already depressed by disease<br>-May require synergy with β-blocker	-Nitric oxide synthase<br>-Membrane fluidity<br>-cAMP content<br>-Coronary flow	31, 45-58
**Ranolazine**	FAO: 3-keto-thiolase	-Pressure-overload<br>-Ischemic Cardiomyopathy	↓	-Improved contractile function in RV hypertrophy model<br>-Reduced fibrosis		-Pyruvate dehydrogenase<br>-Late Na^{+} current	31, 45,59
**Etomoxir**	FAO: CPT-1	-Pressure-overload<br>-Ischemic Cardiomyopathy	↓	-Improved contractile function<br>-Increased active Ca^{2+} pumps	-Increased glucose utilization<br>-Decreased oxygen consumption	-Hyperglycemia and gluconeogenesis	31, 60-62
**Voglibose Acarbose**	**Glucose absorption:** α-Glucosidase	-Pressure-overload and hypertension		-Reduced risk of infarct, cardiovascular events, and hypertension<br>-Reduced oxidative stress<br>-Improved contractile function	-Effects rely on presence of impaired glucose metabolism<br>-Results in elevated fasting plasma glucose and insulin levels<br>-Blunted insulin response to feeding	-Reduced adipose weight and lipogenesis<br>-Reduced body weight at higher doses	63-65
**Streptozotocin**	**Insulin secretion:** β-Cells	-Pressure-overload	↑	-Reduced cardiac weight, dysfunction and dilation<br>-Increased vascular density<br>-Reduced apoptosis	-Reduced pathology and improved function by super-imposing diabetic cardiomyopathy on pressure-overload-induced cardiomyopathy		66

Key: FAO (fatty acid oxidation), carnitine palmitoyl transferase (CPT), phosphocreatine (pCR), fatty acid (FA), right ventricle (RV)
