# Supplementary material for: Metabolic crosstalk between the heart and liver impacts familial hypertrophic cardiomyopathy
Source: EMBO Mol Med. 2014 Feb 24;6(4):482–95. doi: 10.1002/emmm.201302852 (PMC3992075; doi:10.1002/emmm.201302852)
Supplement: Supplementary file 27 [file emmm0006-0482-sd27.pdf]

Supporting Information Table 4

Table 4: qPCR Primer Sequences

Gene		Primer Sequence	Gene		Primer Sequence
11βHsd-1	Fwd	ggA gCA ATT TAT TgT CAA ggC g	Hmgb-1	Fwd	ggC gAg CAT CCT ggC TTA TC
11βHsd-1	Rev	CAT gAC CAC gTA gCT gAg gAA	Hmgb-1	Rev	ggC TgC TTg TCA TCT gCT g
18s	Fwd	CTT TCg CTC Tgg TCC gTC TT	Hnf-4α	Fwd	gAA AAT gTg CAg gTg TTg ACC A
18s	Rev	gCC gCT AgA ggT gAA ATT CTT	Hnf-4α	Rev	AgC TCg Agg CTC CgT AgT gTT t
Angptl-4	Fwd	ggA CTg ggA Tgg CAA TgC	L-Cpt1	Fwd	gTC AAg CCA gAC gAA gAA CA
Angptl-4	Rev	CCT CAC CCC CCA AAT gg	L-Cpt1	Rev	CgA gAA gAC CTT gAC CAT Ag
Anp	Fwd	Agg AgA AgA TgC Cgg TAg AAg A	LpL	Fwd	TgT Tag AgA AgT AgT TCC Ag
Anp	Rev	gCT TCC TCA gTC TgC TCA CTC A	LpL	Rev	gAT gCC ggT gAC AAA TTA TAC
ApoB	Fwd	CTg AAC ATC AAg Agg ggC ATC	Mcp-1	Fwd	TTC CTC CAC CAC CAT gCA g
ApoB	Rev	ggT AAC CTg AgT TgA gCA gTT T	Mcp-1	Rev	CCA gCC ggC AAC TgT gA
β-Myhc	Fwd	CAg gAC ACC AgC gCC CA	M-Cpt1	Fwd	TTC Cgg gAC AAA ggC AAg T
β-Myhc	Rev	CCC TTg gAg CTg ggT AgC AC	M-Cpt1	Rev	gCg gTA CAT gTT TTg gTg CTT
Bnp	Fwd	AAg gTg CTg TCC CAg ATg	Mgam	Fwd	CTT gCC AAT CgA gAT gAC TgT
Bnp	Rev	TTg gTC CTT CAA gAg CTg TC	Mgam	Rev	gTC TgC CTg AAg CCg TgA TAC
Cd36	Fwd	gAT gTg gAA CCC ATA ACT ggA TTC AC	Mgat-2	Fwd	Cgg gCT TTA CCT CgC TTT TC
Cd36	Rev	ggT CCC AgT CTC ATT TAg CCA CAg T	Mgat-2	Rev	CCC AgA CAT gAT gTA ATC TCg gA
Cpt-2	Fwd	ACC CTg CCA gAA gTg ACA C	Mttp	Fwd	ATg ATC CTC TTg gCA gTg CTT
Cpt-2	Rev	ACg AgT TgA ATT gAA AAg CCg AA	Mttp	Rev	TgA gAg gCC AgT TgT gTg AC
Creb	Fwd	CAg ggg TCg CAA ggA TTg Aag	Pepck	Fwd	CAT gAC TCg gAT ggg CAT ATC
Creb	Rev	ATC gCC TgA ggC AgT gTA CT	Pepck	Rev	CAT ATC CgC TTA CAA Agg AgA T
Dgat-1	Fwd	TTC CgC CTC Tgg gCA TT	Pgc-1α	Fwd	TTT gTT TTT CTC TCT CAC CC
Dgat-1	Rev	AgA ATC ggC CCA CAA TCC A	Pgc-1α	Rev	AgC AAg CAT TCg ACA ggA C
Fasn	Fwd	gCT gCg gAA ACT TCA ggA AAT	Rage	Fwd	CTT gCT CTA Tgg ggA gCT gTA
Fasn	Rev	AgA gAC gTg TCA CTC CTG GAC TT	Rage	Rev	ggA ggA TTT gAg CCA CgC T
Fatp-1	Fwd	ACA gCC AgT Tgg ACC CTA ACT CAA	S100a8	Fwd	AAA TCA CCA TgC CCT CTA CAA g
Fatp-1	Rev	Tgg ATC TTg AAG gTg CCT gTg gTA	S100a8	Rev	CCC ACT TTT ATC ACC ATC gCA A
Gata-4	Fwd	CCC TAC CCA gCC TAC ATg g	Serca	Fwd	TgT AAg Tgg CCA gATT gCT C
Gata-4	Rev	ACA TAT CgA gAT Tgg ggT gTC T	Serca	Rev	CCT AAA CAA CTg AAg TTA gg
GlcgnR	Fwd	TTg gCg ATg ACC TCA gTg Tg	Scd-1	Fwd	Tgg gTT ggC TgC TTg Tg
GlcgnR	Rev	CCA gCA ATA gTT ggg TAT gAT gC	Scd-1	Rev	gCg Tgg gCA ggA TgA Ag
Glut-1	Fwd	CCA TCC ACC ACA CTC ACC AC	Scd-2	Fwd	gCA TTT ggg AgC CTT gTA g
Glut-1	Rev	gCC CAg gAT CAg CAT CTC AA	Scd-2	Rev	AgC CgT gCC gTg CCT gTA TgT Tg
Glut-4	Fwd	TCg TCA TTg gCA TTC Tgg TTg	Sk. Actin	Fwd	CgA CAT Cag gAA ggA CCT gTA TgC C
Glut-4	Rev	AgC TCg TTC TAC TAA gAg CAC	Sk. Actin	Rev	AgC CTC gTC gTA CTC CTg CTT gg
GR	Fwd	AgC TCC CCC Tgg TAg AgA C	Tnfa	Fwd	Agg gTC Tgg gCC ATA gAA CT
GR	Rev	ggT gAA gAC gCA gAA ACC TTg	Tnfa	Rev	CCA CCA CgC TCT TCTgTC AC
H-Fabp	Fwd	AAC ggg CAg gAg ACA ACA CTA ACT	Ucp-2	Fwd	ACC AgT TCT ACA CCA Agg gC
H-Fabp	Rev	TCA TAA gTC CgA gTg CTC ACC ACA	Ucp-2	Rev	AgC ATg gTA Agg gCA CAg Tg
IL-1β	Fwd	ggT CAA Agg TTT ggA AgC ggT Ag	Ucp-3	Fwd	TgC TgA gAT ggT gAC CTA Cg
IL-1β	Rev	TgT gAA ATg CCA CCT TTT gA	Ucp-3	Rev	gCg TTC ATg TAT Cgg gTC TT
IL-6	Fwd	ACC AgA ggA AAT TTT CAA Tag gC	Vldlr	Fwd	ggC AgC Agg CAA TgC AAT g
IL-6	Rev	TgA TgC ACT TgC AgA AAA CA	Vldlr	Rev	CCC Agg CAC TgA TTg ACg TTT
*Pepck*_AF1	Fwd	Agg TAA CAC ACC CCA gCT AAC	*Pepck*_CRE	Fwd	CTg ACA ATT AAg gCA AgA gCC
*Pepck*_AF1	Rev	CTg ACA ATT AAg gCA AgA gCC	*Pepck*_CRE	Rev	CCT CTg gga ACA CAC CCT
