# Supplementary material for: Metabolic crosstalk between the heart and liver impacts familial hypertrophic cardiomyopathy
Source: EMBO Mol Med. 2014 Feb 24;6(4):482–95. doi: 10.1002/emmm.201302852 (PMC3992075; doi:10.1002/emmm.201302852)
Supplement: Supplementary file 28 [file emmm0006-0482-sd28.pdf]

Supplemental References

1. Waspe LE, Ordahl CP, Simpson PC. The cardiac beta-myosin heavy chain isogene is induced selectively in alpha 1-adrenergic receptor-stimulated hypertrophy of cultured rat heart myocytes. *The Journal of clinical investigation*. 1990;85:1206-1214
2. Nielsen LB, Veniant M, Boren J, Raabe M, Wong JS, Tam C, Flynn L, Vanni-Reyes T, Gunn MD, Goldberg IJ, Hamilton RL, Young SG. Genes for apolipoprotein b and microsomal triglyceride transfer protein are expressed in the heart: Evidence that the heart has the capacity to synthesize and secrete lipoproteins. *Circulation*. 1998;98:13-16
3. Sharma S, Adrogue JV, Golfman L, Uray I, Lemm J, Youker K, Noon GP, Frazier OH, Taegtmeyer H. Intramyocardial lipid accumulation in the failing human heart resembles the lipotoxic rat heart. *The FASEB journal : official publication of the Federation of American Societies for Experimental Biology*. 2004;18:1692-1700
4. Nakae I, Mitsunami K, Yoshino T, Omura T, Tsutamoto T, Matsumoto T, Morikawa S, Inubushi T, Horie M. Clinical features of myocardial triglyceride in different types of cardiomyopathy assessed by proton magnetic resonance spectroscopy: Comparison with myocardial creatine. *Journal of cardiac failure*. 2010;16:812-822
5. Melenovsky V, Benes J, Skaroupkova P, Sedmera D, Strnad H, Kolar M, Vlcek C, Petrak J, Benes J, Jr., Papousek F, Oliyarnyk O, Kazdova L, Cervenka L. Metabolic characterization of volume overload heart failure due to aorto-caval fistula in rats. *Molecular and cellular biochemistry*. 2011;354:83-96
6. Abassi Z, Goltsman I, Karram T, Winaver J, Hoffman A. Aortocaval fistula in rat: A unique model of volume-overload congestive heart failure and cardiac hypertrophy. *Journal of biomedicine & biotechnology*. 2011;2011:729497
7. Petrak J, Pospisilova J, Sedinova M, Jedelsky P, Lorkova L, Vit O, Kolar M, Strnad H, Benes J, Sedmera D, Cervenka L, Melenovsky V. Proteomic and transcriptomic analysis of heart failure due to volume overload in a rat aorto-caval fistula model provides support for new potential therapeutic targets - monoamine oxidase a and transglutaminase 2. *Proteome science*. 2011;9:69
8. Kato T, Niizuma S, Inuzuka Y, Kawashima T, Okuda J, Kawamoto A, Tamaki Y, Iwanaga Y, Soga T, Kita T, Kimura T, Shioi T. Analysis of liver metabolism in a rat model of heart failure. *International journal of cardiology*. 2011
9. Saddik M, Lopaschuk GD. Myocardial triglyceride turnover during reperfusion of isolated rat hearts subjected to a transient period of global ischemia. *The Journal of biological chemistry*. 1992;267:3825-3831
10. Nikolaidis LA, Sturzu A, Stolarski C, Elahi D, Shen YT, Shannon RP. The development of myocardial insulin resistance in conscious dogs with advanced dilated cardiomyopathy. *Cardiovascular research*. 2004;61:297-306

11. Shen W, Asai K, Uechi M, Mathier MA, Shannon RP, Vatner SF, Ingwall JS. Progressive loss of myocardial atp due to a loss of total purines during the development of heart failure in dogs: A compensatory role for the parallel loss of creatine. *Circulation*. 1999;100:2113-2118
12. Weiss RG, Gerstenblith G, Bottomley PA. Atp flux through creatine kinase in the normal, stressed, and failing human heart. *Proceedings of the National Academy of Sciences of the United States of America*. 2005;102:808-813
13. Spindler M, Saupe KW, Christe ME, Sweeney HL, Seidman CE, Seidman JG, Ingwall JS. Diastolic dysfunction and altered energetics in the alphamhc403/+ mouse model of familial hypertrophic cardiomyopathy. *The Journal of clinical investigation*. 1998;101:1775-1783
14. Tanaka T, Sohmiya K, Kawamura K. Is cd36 deficiency an etiology of hereditary hypertrophic cardiomyopathy? *Journal of molecular and cellular cardiology*. 1997;29:121-127
15. Paiva MA, Goncalves LM, Providencia LA, Davidson SM, Yellon DM, Mocanu MM. Transitory activation of ampk at reperfusion protects the ischaemic-reperfused rat myocardium against infarction. *Cardiovascular drugs and therapy / sponsored by the International Society of Cardiovascular Pharmacotherapy*. 2010;24:25-32
16. Tian R, Musi N, D'Agostino J, Hirshman MF, Goodyear LJ. Increased adenosine monophosphate-activated protein kinase activity in rat hearts with pressure-overload hypertrophy. *Circulation*. 2001;104:1664-1669
17. Shimano M, Ouchi N, Shibata R, Ohashi K, Pimentel DR, Murohara T, Walsh K. Adiponectin deficiency exacerbates cardiac dysfunction following pressure overload through disruption of an ampk-dependent angiogenic response. *Journal of molecular and cellular cardiology*. 2010;49:210-220
18. Baron SJ, Li J, Russell RR, 3rd, Neumann D, Miller EJ, Tuerk R, Wallimann T, Hurley RL, Witters LA, Young LH. Dual mechanisms regulating ampk kinase action in the ischemic heart. *Circulation research*. 2005;96:337-345
19. Sasaki H, Asanuma H, Fujita M, Takahama H, Wakeno M, Ito S, Ogai A, Asakura M, Kim J, Minamino T, Takashima S, Sanada S, Sugimachi M, Komamura K, Mochizuki N, Kitakaze M. Metformin prevents progression of heart failure in dogs: Role of amp-activated protein kinase. *Circulation*. 2009;119:2568-2577
20. Zen K, Irie H, Doue T, Takamiya M, Yamano T, Sawada T, Azuma A, Matsubara H. Analysis of circulating apoptosis mediators and proinflammatory cytokines in patients with idiopathic hypertrophic cardiomyopathy: Comparison between nonobstructive and dilated-phase hypertrophic cardiomyopathy. *International heart journal*. 2005;46:231-244
21. Torre-Amione G. Immune activation in chronic heart failure. *The American journal of cardiology*. 2005;95:3C-8C; discussion 38C-40C
22. Ingelsson E, Arnlov J, Sundstrom J, Zethelius B, Vessby B, Lind L. Novel metabolic risk factors for heart failure. *Journal of the American College of Cardiology*. 2005;46:2054-2060

23. Yin M, van der Horst IC, van Melle JP, Qian C, van Gilst WH, Sillje HH, de Boer RA. Metformin improves cardiac function in a nondiabetic rat model of post-mi heart failure. *American journal of physiology. Heart and circulatory physiology*. 2011;301:H459-468
24. Murakami K, Shigematsu Y, Hamada M, Higaki J. Insulin resistance in patients with hypertrophic cardiomyopathy. *Circulation journal : official journal of the Japanese Circulation Society*. 2004;68:650-655
25. Matsumori A, Yamada T, Suzuki H, Matoba Y, Sasayama S. Increased circulating cytokines in patients with myocarditis and cardiomyopathy. *British heart journal*. 1994;72:561-566
26. Okada M, Mitsunami K, Inubushi T, Kinoshita M. Influence of aging or left ventricular hypertrophy on the human heart: Contents of phosphorus metabolites measured by 31p mrs. *Magnetic resonance in medicine : official journal of the Society of Magnetic Resonance in Medicine / Society of Magnetic Resonance in Medicine*. 1998;39:772-782
27. Lefroy DC, de Silva R, Choudhury L, Uren NG, Crake T, Rhodes CG, Lammertsma AA, Boyd H, Patsalos PN, Nihoyannopoulos P, et al. Diffuse reduction of myocardial beta-adrenoceptors in hypertrophic cardiomyopathy: A study with positron emission tomography. *Journal of the American College of Cardiology*. 1993;22:1653-1660
28. Wang XF, Zhang JY, Li L, Zhao XY, Tao HL, Zhang L. Metformin improves cardiac function in rats via activation of amp-activated protein kinase. *Clinical and experimental pharmacology & physiology*. 2011;38:94-101
29. Li HL, Yin R, Chen D, Liu D, Wang D, Yang Q, Dong YG. Long-term activation of adenosine monophosphate-activated protein kinase attenuates pressure-overload-induced cardiac hypertrophy. *Journal of cellular biochemistry*. 2007;100:1086-1099
30. Armstrong PW, Stopps TP, Ford SE, de Bold AJ. Rapid ventricular pacing in the dog: Pathophysiologic studies of heart failure. *Circulation*. 1986;74:1075-1084
31. Lopaschuk GD, Ussher JR, Folmes CD, Jaswal JS, Stanley WC. Myocardial fatty acid metabolism in health and disease. *Physiological reviews*. 2010;90:207-258
32. Anker SD, von Haehling S. Inflammatory mediators in chronic heart failure: An overview. *Heart*. 2004;90:464-470
33. Suskin N, McKelvie RS, Burns RJ, Latini R, Pericak D, Probstfield J, Rouleau JL, Sigouin C, Solymoss CB, Tsuyuki R, White M, Yusuf S. Glucose and insulin abnormalities relate to functional capacity in patients with congestive heart failure. *European heart journal*. 2000;21:1368-1375
34. Benes J, Kazdova L, Drahota Z, Houstek J, Medrikova D, Kopecky J, Kovarova N, Vrbacky M, Sedmera D, Strnad H, Kolar M, Petrak J, Benada O, Skaroupkova P, Cervenka L, Melenovsky V. Effect of metformin therapy on cardiac function and survival in a volume-overload model of heart failure in rats. *Clinical science*. 2011;121:29-41

35. Mahrouf M, Ouslimani N, Peynet J, Djelidi R, Couturier M, Therond P, Legrand A, Beaudeux JL. Metformin reduces angiotensin-mediated intracellular production of reactive oxygen species in endothelial cells through the inhibition of protein kinase c. *Biochemical pharmacology*. 2006;72:176-183
36. Xiao H, Ma X, Feng W, Fu Y, Lu Z, Xu M, Shen Q, Zhu Y, Zhang Y. Metformin attenuates cardiac fibrosis by inhibiting the tgfbeta1-smad3 signalling pathway. *Cardiovascular research*. 2010;87:504-513
37. Kim M, Tian R. Targeting ampk for cardiac protection: Opportunities and challenges. *Journal of molecular and cellular cardiology*. 2011;51:548-553
38. Moopanar TR, Xiao XH, Jiang L, Chen ZP, Kemp BE, Allen DG. Aicar inhibits the na+/h+ exchanger in rat hearts--possible contribution to cardioprotection. *Pflugers Archiv : European journal of physiology*. 2006;453:147-156
39. Russell RR, 3rd, Bergeron R, Shulman GI, Young LH. Translocation of myocardial glut-4 and increased glucose uptake through activation of ampk by aicar. *The American journal of physiology*. 1999;277:H643-649
40. Paiva M, Riksen NP, Davidson SM, Hausenloy DJ, Monteiro P, Goncalves L, Providencia L, Rongen GA, Smits P, Mocanu MM, Yellon DM. Metformin prevents myocardial reperfusion injury by activating the adenosine receptor. *Journal of cardiovascular pharmacology*. 2009;53:373-378
41. Abozguia K, Clarke K, Lee L, Frenneaux M. Modification of myocardial substrate use as a therapy for heart failure. *Nature clinical practice. Cardiovascular medicine*. 2006;3:490-498
42. Abozguia K, Elliott P, McKenna W, Phan TT, Nallur-Shivu G, Ahmed I, Maher AR, Kaur K, Taylor J, Henning A, Ashrafian H, Watkins H, Frenneaux M. Metabolic modulator perhexiline corrects energy deficiency and improves exercise capacity in symptomatic hypertrophic cardiomyopathy. *Circulation*. 2010;122:1562-1569
43. Horowitz JD, Chirkov YY. Perhexiline and hypertrophic cardiomyopathy: A new horizon for metabolic modulation. *Circulation*. 2010;122:1547-1549
44. Lee L, Campbell R, Scheuermann-Freestone M, Taylor R, Gunaruwan P, Williams L, Ashrafian H, Horowitz J, Fraser AG, Clarke K, Frenneaux M. Metabolic modulation with perhexiline in chronic heart failure: A randomized, controlled trial of short-term use of a novel treatment. *Circulation*. 2005;112:3280-3288
45. Ormerod JO, Ashrafian H, Frenneaux MP. Impaired energetics in heart failure - a new therapeutic target. *Pharmacology & therapeutics*. 2008;119:264-274
46. Monti LD, Setola E, Fragasso G, Camisasca RP, Lucotti P, Galluccio E, Origgi A, Margonato A, Piatti P. Metabolic and endothelial effects of trimetazidine on forearm skeletal muscle in patients with type 2 diabetes and ischemic cardiomyopathy. *American journal of physiology. Endocrinology and metabolism*. 2006;290:E54-E59

47. Onay-Besikci A, Ozkan SA. Trimetazidine revisited: A comprehensive review of the pharmacological effects and analytical techniques for the determination of trimetazidine. *Cardiovascular therapeutics*. 2008;26:147-165
48. Tuunanen H, Engblom E, Naum A, Nagren K, Scheinin M, Hesse B, Juhani Airaksinen KE, Nuutila P, Iozzo P, Ukkonen H, Opie LH, Knuuti J. Trimetazidine, a metabolic modulator, has cardiac and extracardiac benefits in idiopathic dilated cardiomyopathy. *Circulation*. 2008;118:1250-1258
49. Zemljic G, Bunc M, Vrtovec B. Trimetazidine shortens qtc interval in patients with ischemic heart failure. *Journal of cardiovascular pharmacology and therapeutics*. 2010;15:31-36
50. Liu X, Gai Y, Liu F, Gao W, Zhang Y, Xu M, Li Z. Trimetazidine inhibits pressure overload-induced cardiac fibrosis through nadph oxidase-ros-ctgf pathway. *Cardiovascular research*. 2010;88:150-158
51. Di Napoli P, Chierchia S, Taccardi AA, Grilli A, Felaco M, De Caterina R, Barsotti A. Trimetazidine improves post-ischemic recovery by preserving endothelial nitric oxide synthase expression in isolated working rat hearts. *Nitric oxide : biology and chemistry / official journal of the Nitric Oxide Society*. 2007;16:228-236
52. Di Napoli P, Di Giovanni P, Gaeta MA, D'Apolito G, Barsotti A. Beneficial effects of trimetazidine treatment on exercise tolerance and b-type natriuretic peptide and troponin t plasma levels in patients with stable ischemic cardiomyopathy. *American heart journal*. 2007;154:602 e601-605
53. Di Napoli P, Di Giovanni P, Gaeta MA, Taccardi AA, Barsotti A. Trimetazidine and reduction in mortality and hospitalization in patients with ischemic dilated cardiomyopathy: A post hoc analysis of the villa pini d'abruzzo trimetazidine trial. *Journal of cardiovascular pharmacology*. 2007;50:585-589
54. Di Napoli P, Taccardi AA. Trimetazidine: The future of cardiac function? *Future cardiology*. 2009;5:421-424
55. Fragasso G, Montano C, Perseghin G, Palloshi A, Calori G, Lattuada G, Oggionni S, Bassanelli G, Locatelli M, Lopaschuk G, Margonato A. The anti-ischemic effect of trimetazidine in patients with postprandial myocardial ischemia is unrelated to meal composition. *American heart journal*. 2006;151:1238 e1231-1238
56. Fragasso G, Palloshi A, Puccetti P, Silipigni C, Rossodivita A, Pala M, Calori G, Alfieri O, Margonato A. A randomized clinical trial of trimetazidine, a partial free fatty acid oxidation inhibitor, in patients with heart failure. *Journal of the American College of Cardiology*. 2006;48:992-998
57. Fragasso G, Perseghin G, De Cobelli F, Esposito A, Palloshi A, Lattuada G, Scifo P, Calori G, Del Maschio A, Margonato A. Effects of metabolic modulation by trimetazidine on left ventricular function and

phosphocreatine/adenosine triphosphate ratio in patients with heart failure. *European heart journal*. 2006;27:942-948
58. Fragasso G, Piatti PM, Monti L, Palloshi A, Lu C, Valsecchi G, Setola E, Calori G, Pozza G, Margonato A, Chierchia S. Acute effects of heparin administration on the ischemic threshold of patients with coronary artery disease: Evaluation of the protective role of the metabolic modulator trimetazidine. *Journal of the American College of Cardiology*. 2002;39:413-419
59. Fang YH, Piao L, Hong Z, Toth PT, Marsboom G, Bache-Wiig P, Rehman J, Archer SL. Therapeutic inhibition of fatty acid oxidation in right ventricular hypertrophy: Exploiting randle's cycle. *Journal of molecular medicine*. 2012;90:31-43
60. Turcani M, Rupp H. Etomoxir improves left ventricular performance of pressure-overloaded rat heart. *Circulation*. 1997;96:3681-3686
61. Reaven GM, Chang H, Hoffman BB. Additive hypoglycemic effects of drugs that modify free-fatty acid metabolism by different mechanisms in rats with streptozocin-induced diabetes. *Diabetes*. 1988;37:28-32
62. Lopaschuk GD, Wall SR, Olley PM, Davies NJ. Etomoxir, a carnitine palmitoyltransferase i inhibitor, protects hearts from fatty acid-induced ischemic injury independent of changes in long chain acylcarnitine. *Circulation research*. 1988;63:1036-1043
63. Chiasson JL, Josse RG, Gomis R, Hanefeld M, Karasik A, Laakso M. Acarbose treatment and the risk of cardiovascular disease and hypertension in patients with impaired glucose tolerance: The stop-niddm trial. *JAMA : the journal of the American Medical Association*. 2003;290:486-494
64. Maury J, Issad T, Perdereau D, Gouhot B, Ferre P, Girard J. Effect of acarbose on glucose homeostasis, lipogenesis and lipogenic enzyme gene expression in adipose tissue of weaned rats. *Diabetologia*. 1993;36:503-509
65. Liao Y, Takashima S, Zhao H, Asano Y, Shintani Y, Minamino T, Kim J, Fujita M, Hori M, Kitakaze M. Control of plasma glucose with alpha-glucosidase inhibitor attenuates oxidative stress and slows the progression of heart failure in mice. *Cardiovascular research*. 2006;70:107-116
66. Shimizu I, Minamino T, Toko H, Okada S, Ikeda H, Yasuda N, Tateno K, Moriya J, Yokoyama M, Nojima A, Koh GY, Akazawa H, Shiojima I, Kahn CR, Abel ED, Komuro I. Excessive cardiac insulin signaling exacerbates systolic dysfunction induced by pressure overload in rodents. *The Journal of clinical investigation*. 2010;120:1506-1514
67. Sharma R, Coats AJ, Anker SD. The role of inflammatory mediators in chronic heart failure: Cytokines, nitric oxide, and endothelin-1. *International journal of cardiology*. 2000;72:175-186
