# Supplementary material for: Metabolic crosstalk between the heart and liver impacts familial hypertrophic cardiomyopathy
Source: EMBO Mol Med. 2014 Feb 24;6(4):482–95. doi: 10.1002/emmm.201302852 (PMC3992075; doi:10.1002/emmm.201302852)
Supplement: Supplementary file 29 [file emmm0006-0482-sd29.pdf]

Supplemental Materials and Methods

Western blotting antibodies

Western blotting was performed using phospho-AMPK (Thr172, #2531), total AMPK (#2532), phospho-ASK1 (Ser83, #3761), total ASK1 (#3762), phospho-p38 MAPK (Thr180/Tyr182, #9261), total p38 MAPK (#9212), phospho-SAPK/JNK (Thr183/Tyr185, #9255), total SAPK/JNK (#9252), phospho-PKCδ/θ (Ser643/676, #9376), total PKCδ/θ (#2058), phospho-ACC (#3661) antibodies from Cell Signaling Technology, pan phospho-protein antibody (#61-8399) from zymed, CD36 (#9154) PKCα (#8393), PKCβI (#209), PKCβII (#210), PKCγ (#211), PKCθ (#212), PGC-1 (#13067), HNF-4α (#6556), FOXO1 (#11350) from Santa Cruz Biotechnology, β-actin (#A5441) from Sigma, PPARα (#MA1-822) from Affinity Bioreagents, APOB (AB742) from Millipore. The lyn, phospho-protein, PKC, caspase 3 antibodies were kind gifts from the laboratories of Drs. J. Martin, G. Odorizzi, B. Olwin and J. Espinosa.

Immunoprecipitation

262.5µg (p38 MAPK and PKCα IP) or 500µg (PGC1 IP) of liver lysates were pre-cleared with nonspecific rabbit IgG (Sigma #A5420) and protein A/G-conjugated agarose beads (Santa Cruz #2003) for approximately one hour, washed, then centrifuged. The supernatants were transferred to new tubes containing nonspecific IgG, anti-p38 MAPK, PKC or PGC1 antibodies and rotated overnight. Protein A/G-conjugated agarose beads were added to the tubes and rotated overnight, then washed with PBS and RIPA buffer. The beads were then boiled with β-mercaptoethanol-containing loading buffer for SDS-PAGE.

Chromatin Immunoprecipitation (adapted from Shlomai et al., PNAS, 2006)

50mg liver was minced in 950µl 1% formaldehyde, then combined with 50µl 2.5M glycine and rotated 5 minutes at room temperature. Samples were pelleted (10k x g at 4°C), washed and resuspended with 1ml Buffer1 (10mM HEPES pH6.5, 10mM EDTA, 0.5mM EGTA, 0.25% tritonX-100), then pelleted and resuspended with Buffer2 (10mM HEPES, 1mM EDTA, 0.5mM EGTA, 200mM NaCl) and aggregates dispersed with 18g needle. Samples were pelleted and resuspended with lysis buffer (50mM Tris pH8.1, 10mM EDTA, 1% SDS, 0.8µg/ml pepstatin A, 0.6µg/ml leupeptin, 1mM PMSF) and sonicated. Extracts were clarified 10k x g spin (15minutes at 4°C). 900µl supernatant was added to 8.1ml dilution buffer (20mM Tris pH8.1, 150mM NaCl, 2mM EDTA, 1% tritonX-100). Sample protein content was quantified and diluted to 0.2µg/µl (in 500µl) dilution buffer. 5µl salmon sperm DNA, 10µl tRNA, and 100µl 10mg/ml BSA were added to each tube. Extracts were precleared (rotated for 30 minutes at 4°C) with 30µl washed protein A/G conjugated beads, then centrifuged. The supernatant was removed to a new tube containing 5µl antibody and rotated overnight at 4°C. Pellets were attained centrifugation and washed sequentially with TSE (20mM Tris, 2mM

EDTA, 1% tritonX-100) + 150mM NaCl, TSE+500mM NaCl, then buffer3 (10mM Tris, 250mM LiCl, 1mM EDTA, 1%NP-40, 1% deoxycholate, then three times in 10mM Tris/1mM EDTA. 4 volumes of eluent (1%SDS, 100mM Na bicarbonate, 20µg/ml glycogen) were collected and incubated at 65^oC for 4 hours. PCR was performed with 10µCi/reaction ^{32}P-dCTP for visualization on an 8% acrylamide gel and validated with Sybr green on the ABI7500 system.

Chromatography

For TLC, total lipids were extracted from (30 mg) ventricles using a modified Folch extraction with chloroform-methanol-formic acid (10:10:1 v/v). 35µL (liver) or 50µL (heart) of the lipid-containing organic phase from each sample was spotted onto silica gel G TLC plates (Sigma no. Z12,277-7), run using a hexane:diethyl ether:acetic acid (40:10:1) mobile phase and developed using iodine. For gas chromatography analysis of fatty acid composition, liver extracts were transesterified in 1% sulfuric acid in methanol at 85^oC for 1 hour. Fatty acid methyl esters were then extracted with hexane. Fatty acid methyl esters were separated and quantified by capillary gas chromatography (Agilent 6890N) equipped with a DB-23 column (30m x 250µm x 0.25µm) and a flame-ionization detector.

Transverse Aortic constriction

A left thoracotomy was performed through the second intercostal space, and the aorta isolated. A 6-0 silk suture was placed around the aorta between the innominate artery and the left carotid artery and tied against a 26G needle to reach a fixed diameter. The chest was closed in three layers. The intercostal muscle and ribcage was closed with interrupted 5-0 chromic gut and the pectoral muscle and skin layer with uninterrupted 5-0 silk suture. A chest tube (0.64mm silastic) was inserted in the chest at the time of closure and the pleural space evacuated to establish proper intrapleural pressure and remove any fluid. Mice were 3-4 months old at the time of surgery, and 6 months old at the time of sacrifice.

Blood Pressure Measurement

The mice were anesthetized with 3% Isoflurane and 2% O2, and maintained on at a surgical plane of isoflurane anesthesia (2% via nosecone). The right carotid artery was dissected and isolated. A heat pulled PE50 fluid filled catheter was placed in the carotid artery. Steady-state systemic hemodynamics was obtained using the I-Worx (model BP-100) fluid filled catheter system.

Plasma membrane fractionation

80 mg of frozen cardiac tissue was homogenized in 800µl cold buffer (10mM $HaHCO_3$, 250mM sucrose, 5mM NaN_3, 0.1mM PMSF, 5µg/mL leupeptin, 5µg/mL

aprotinin, and 1μg/mL pepstatin), pH7.4, and centrifuged for 10 minutes at 1000x g. The supernatant (S1) was saved to a new tube, while the pellet (P1) was resuspended in buffer, homogenized and centrifuged 10 minutes at 1000x g. The supernatant (S2) was added to S1 and centrifuged 10 minutes at 9000x g. The supernatant (S3) was then centrifuged 60 minutes at 190000x g and the resultant pellet (P4) was resuspended in 300μl buffer and applied to 25%, 30%, 35% discontinuous sucrose gradient and centrifuged for an additional 16 hours at 190000x g. PM and GSV fractions were collected from the top of the 25% and 35% sucrose steps, resuspended in 750μl buffer and centrifuged 60 minutes at 190000x g. The resultant pellets (P5) were resuspended in RIPA and loading buffer for SDS-PAGE.

ROS and AGE Assays

For quantification of tissue reactive oxygen species (ROS) and advanced glycated end-products (AGE), 35mg of wet tissue weight was homogenized and sonicated in cold PBS, spun to remove debris, and supernatant retained for assays. ROS and AGE were determined using the OxiSelect ROS/RNS Assay and AGE ELISA kits (Cell Biolabs, #STA-347 and STA-317, respectively). ROS and AGE quantities were normalized to protein content.

NRVM cell culture. Neonatal rat ventricular myocytes (NRVMs) were prepared according to the method described by Waspe et al. Cells were maintained in 12-well gelatinized culture plates at a density of 200,000 cells/ml. NRVMs were supplemented with H_2O_2 at a final concentration of 10μM.

Echocardiography

$$\text{LV Volume} = \frac{7}{2.4+(10\ \text{X systolic LVID})}\ \text{X}\ (10\ \text{X systolic LVID})^3$$

$$\text{Ejection Fraction} = 100\ \text{X}\ \frac{\text{diastolic LV Vol} - \text{systolic LV Vol}}{\text{diastolic LV Vol}}$$

$$\text{Fractional Shortening} = 100\ \text{X}\ \frac{(10\ \text{X diastolic LVID}) - (10\ \text{X systolic LVID})}{\text{diastolic LVID}}$$
